# Convergent syntheses of Le^X^ analogues

**DOI:** 10.3762/bjoc.6.17

**Published:** 2010-02-22

**Authors:** An Wang, Jenifer Hendel, France-Isabelle Auzanneau

**Affiliations:** 1 Department of Chemistry, University of Guelph, Guelph, Ontario, N1G 2W1, Canada

**Keywords:** Birch reduction, convergent synthesis, desulfurization, Lewis X

## Abstract

The synthesis of three Le^x^ derivatives from one common protected trisaccharide is reported. These analogues will be used respectively for competitive binding experiments, conjugation to carrier proteins and immobilization on gold. An *N*-acetylglucosamine monosaccharide acceptor was first glycosylated at O-4 with a galactosyl imidate. This coupling was performed at 40 °C under excess of BF_3_·OEt_2_ activation and proceeded best if the acceptor carried a 6-chlorohexyl rather than a 6-azidohexyl aglycon. The 6-chlorohexyl disaccharide was then converted to an acceptor and submitted to fucosylation yielding the corresponding protected 6-chlorohexyl Le^x^ trisaccharide. This protected trisaccharide was used as a precursor to the 6-azidohexyl, 6-acetylthiohexyl and 6-benzylthiohexyl trisaccharide analogues which were obtained in excellent yields (70–95%). In turn, we describe the deprotection of these intermediates in one single step using dissolving metal conditions. Under these conditions, the 6-chlorohexyl and 6-azidohexyl intermediates led respectively to the *n*-hexyl and 6-aminohexyl trisaccharide targets. Unexpectedly, the 6-acetylthiohexyl analogue underwent desulfurization and gave the *n*-hexyl glycoside product, whereas the 6-benzylthiohexyl analogue gave the desired disulfide trisaccharide dimer. This study constitutes a particularly efficient and convergent preparation of these three Le^x^ analogues.

## Introduction

**Figure 1:**
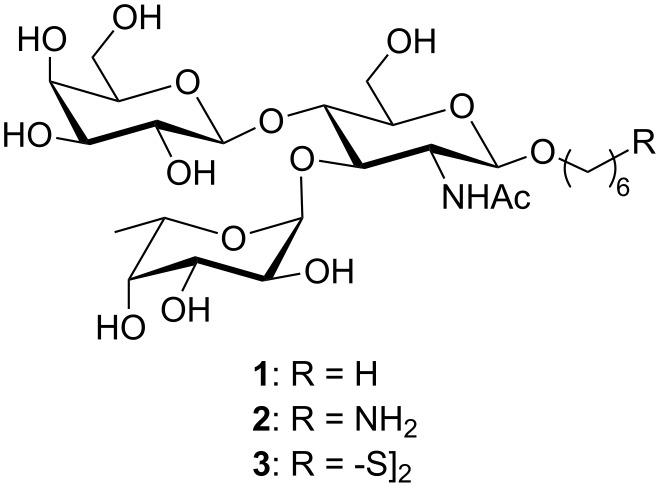
Structure of Le^x^ analogues **1**–**3**.

**Scheme 1:**
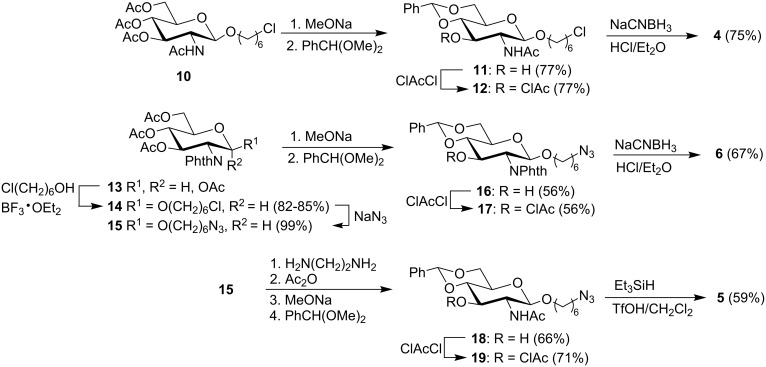
Synthesis of monosaccharide glycosyl acceptors **4**–**6**.

Our group is involved in the design of new anti-cancer vaccines based on the Tumor Associated Carbohydrate Antigen (TACA) dimeric Le^x^ (dimLe^x^) [1–6]. This tumor specific antigen consists of a hexasaccharide that displays the Le^x^ trisaccharide antigen linked to O-3″ of the galactose residue of another Le^x^ trisaccharide. Since it was first characterized [7–8], the Le^x^ antigenic determinant, β-D-Gal*p*(1,4)[α-LFuc*p*(1,3)]-D-GlcNAc*p*, has been found on numerous cells and tissues such as kidney tubules, gastrointestinal epithelial cells, and cells of the spleen and brain [9–11]. Thus, there are numerous reports in the literature that deal with the chemical [12–36] or chemoenzymatic [37–38] preparation of Le^x^ analogues as well as that of Le^x^ intermediate building blocks to be further converted into the Sialyl Le^x^ tetrasaccharide. The chemical syntheses usually follow one of three synthetic schemes: 1. a stepwise approach involving the successive galactosylation then fucosylation of a glucosamine acceptor [12–28]; 2. a stepwise approach in which the sequence of glycosylation of the glucosamine acceptor is reversed, i.e. the fucosylation is followed by the galactosylation [28–34]; 3. a block approach in which a lactosamine derivative prepared from lactose is subjected to fucosylation at O-3 [35–36]. Whereas these reports usually describe the preparation of one compound to be used in a specific experiment, we describe here the convergent synthesis of the three Le^x^ derivatives **1**–**3** (Figure 1) from one common protected trisaccharide intermediate. These three Le^x^ analogues (**1**–**3**) will be used respectively for competitive binding experiments (**1**), conjugation to carrier proteins (**2**) and immobilization to a gold plate (**3**).

## Results and Discussion

**Figure 2:**
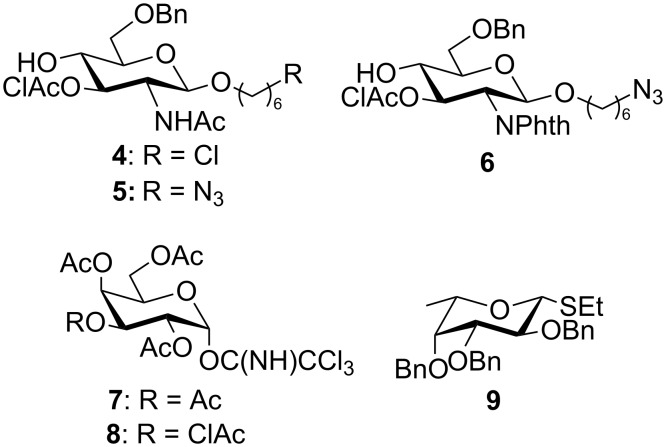
Monosaccharide glycosyl acceptors (**4**–**6**) and donors (**7**–**9**) used in this study.

Our synthetic approach to prepare these Le^x^ derivatives began with the galactosylation at O-4 of glycosyl acceptor **4** with the known [39–41] galactosyl donor **7** followed by deprotection at O-3 of the glucosamine residue and fucosylation of the resulting disaccharide with the known [42] ethylthioglycoside **9**. Since in addition to the Le^x^ trisaccharide we are also interested in preparing fragments of the dimLe^x^ antigen, we examined the glycosylation at O-4 of glucosamine glycosyl acceptors with galactosyl donor **8**, which is chloroacetylated rather than acetylated at O-3. Finally, we also investigated the reactivity towards glycosylation of the *N*-acetylated and phthalimido acceptors **5** and **6**, respectively, that both carry a 6-azidohexyl aglycon (Figure 2).

**Synthesis of monosaccharide building blocks.** The 6-chlorohexyl acceptor **4** was prepared in four steps from the known [43] chlorohexyl glucoside **10** (Scheme 1). Thus, peracetate **10** was deacetylated (NaOMe/MeOH) and converted to the benzylidene acetal **11** by reaction with benzaldehyde dimethyl acetal under camphorsulfonic acid (CSA) catalysis. Chloroacetylation of alcohol **11** gave the intermediate **12** which was converted to acceptor **4** via the reductive opening of the benzylidene acetal using NaCNBH_3_ and HCl·Et_2_O in anhydrous THF at 0 °C.

Both 6-azidohexyl acceptors **6** and **5** were prepared from the anomeric mixture of the known tetraacetate **13** [44]. Thus, tetraacetate **13** was reacted with 6-chlorohexanol (4 equiv) in the presence of BF_3_·OEt_2_ (5 equiv). To promote coupling, the reaction mixture was either stirred for 1 h at 50 °C in an oil bath (Supporting Information File 1, Method A) or submitted to microwave irradiation for 5 min at 50 °C (Supporting Information File 1, Method B). After acetylation of the excess chlorohexanol to ease its removal, pure glycoside **14** was isolated in excellent yield whether method A or B was followed. Thus, these syntheses of glycoside **14** constitute efficient alternatives to that reported by Nitz et al. in which the starting material was the corresponding anomeric bromide [45]. Nucleophilic displacement of the chlorine atom in glycoside **14** (NaN_3_, DMF, 80 °C) gave the known [46] 6-azidohexyl glycoside **15** quantitatively. Zemplén deacetylation of triacetate **15** followed by conversion of the triol to the 4,6-benzylidene acetal (**16**) and then chloroacetylation at O-3 gave intermediate **17** that was submitted to reductive opening of the benzylidene group (NaCNBH_3_, HCl·Et_2_O) to yield acceptor **6**.

The triacetate **15** was also converted in seven steps to acceptor **5**. The phthalimido group was first removed (ethylenediamine, EtOH) and the free amine acetylated. Zemplén deacetylation was followed by conversion of the triol to the 4,6-benzylidene acetal **18** which was chloroacetylated at O-3 to give the fully protected intermediate **19**. Finally, the benzylidene acetal in compound **19** was reductively opened with Et_3_SiH and TfOH in CH_2_Cl_2_ at −30 °C to give acceptor **5**.

**Scheme 2:**
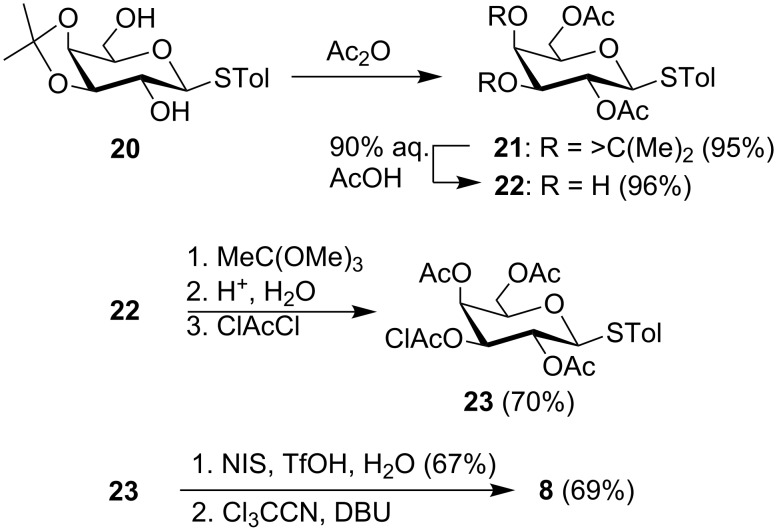
Synthesis of the galactosyl donor **8**.

The trichloroacetimidate glycosyl donor **8** was prepared from the *p*-thiotolyl glycoside **20** [47] (Scheme 2). Diol **20** was first acetylated to the diacetate **21** which was then treated with 90% AcOH at 70 °C to remove the isopropylidene group affording diol **22**. The diol **22** was selectively acetylated at O-4 by converting it to the corresponding cyclic methylorthoacetate and opening the orthoacetate in situ by adding water to the reaction mixture. The resulting triacetate was chloroacetylated at O-3 and the resulting fully protected thioglycoside **23** was converted to the corresponding hemiacetal that was, in turn, treated with trichloroacetonitrile and DBU to give the α-trichloroacetimidate galactosyl donor **8**.

**Glycosylation at O-4 of glucosamine acceptors.** It is well known that the hydroxyl group at C-4 of *N*-acetylglucosamine is a poor nucleophile and has reduced reactivity towards glycosylation when compared to other acceptors [48–50]. However, we have recently reported the successful O-4 glucosylation of an *N*-acetylglucosamine monosaccharide acceptor using a peracetylated glucopyranose α-trichloroacetimidate donor under activation with 2 equiv of BF_3_·OEt_2_ at room temperature [51]. We applied similar conditions: 2 equiv BF_3_·OEt_2_, 5 equiv of donor, 1 h at 40 °C for the coupling of donors **7** and **8** with the acceptors **4**-**6** (Table 1). As can be seen in Table 1 the 6-chlorohexyl glycoside acceptor **4** was easily glycosylated with either donors **7** or **8**, affording the desired disaccharides **24** and **25** in about 70% yield for both reactions (entries 1 and 2).

**Table 1:** Glycosylation at O-4 of glucosamine acceptors **4**–**6**^a^.

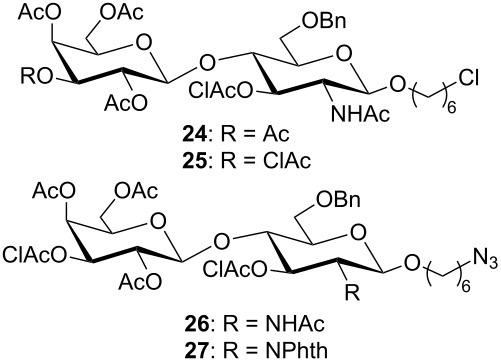			
Entry	Donor	Acceptor	Product (%)

1	**7**	**4**	**24** (69%)
2	**8**	**4**	**25** (72%)
3	**8**	**5**	**26** (27%)^b^
4	**8**	**6**	**27** (11%)

^a^Reagents and conditions: BF_3_·OEt_2_ (2 equiv), donor (5 equiv), CH_2_Cl_2_, 40 °C, 1 h. ^b^Contaminated with degraded acceptor.

In contrast, the coupling of donor **8** with the 6-azidohexyl glycoside acceptor **5** did not proceed well (entry 3). Monitoring of the reaction by TLC showed degradation of the acceptor, and isolation of the desired disaccharide required both silica gel chromatography and RP-HPLC. Indeed, despite our efforts, and even though its structure was confirmed by NMR and HR-ESI mass spectrometry, disaccharide **26** could not be isolated free of degraded acceptor and/or disaccharide. To further test if the *N*-acetyl group was impacting negatively the glycosylation of acceptor **5**, we attempted to couple trichloroacetimidate **8** with the phthalimido acceptor **6**. However as can be seen in Table 1, entry 4, this glycosylation also gave disappointing results: TLC showed a considerable amount of degraded products and the isolation of the desired disaccharide from the reaction mixture required both silica gel chromatography and RP-HPLC. In this case, the disaccharide **27** could be obtained pure albeit in very low yield. These last two reactions suggest that the presence of the azido group on the hexyl aglycon carried by acceptors **5** and **6** is not compatible with the glycosylation conditions that we have established previously [51] for the glycosylation at O-4 of glucosamine acceptors. The disaccharide **24** was further used in the preparation of the Le^x^ analogues **1**–**3**.

**Scheme 3:**
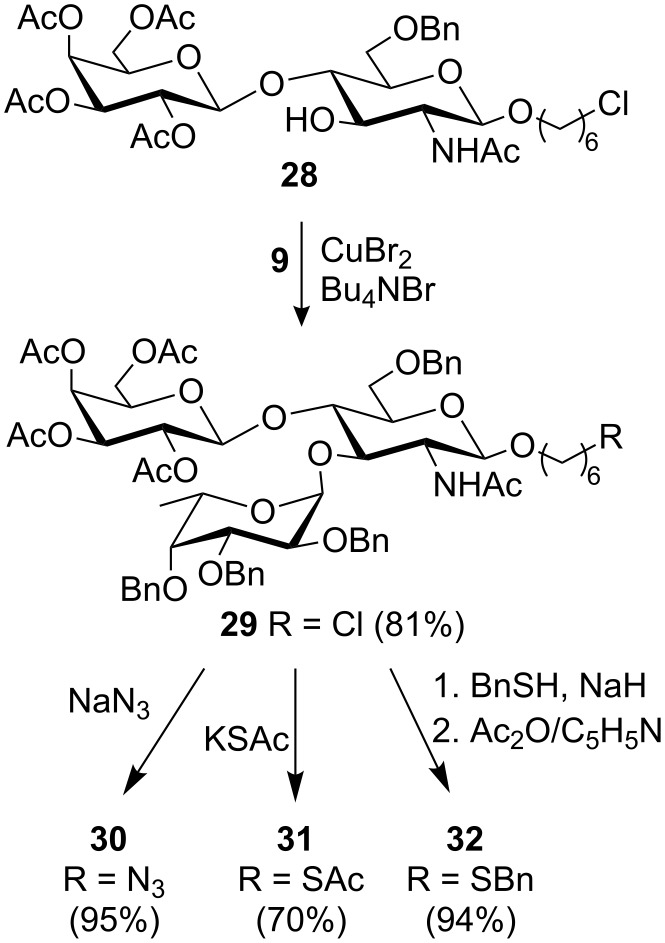
Convergent synthesis of trisaccharides **29**–**32**.

**Preparation of protected Le^x^ analogues.** The chloroacetate in disaccharide **24** was removed with thiourea (C_5_H_5_N/EtOH, 70 °C) to give the acceptor disaccharide **28** (61%), which was then fucosylated with the thioethyl glycoside **9** under copper (II) bromide–tetrabutylammonium bromide activation (Scheme 3). The desired Le^x^ trisaccharide **29** was obtained in excellent yield and the α-configuration of the newly formed fucosidic bond was confirmed by ^1^H NMR (*J*_H-1′,H-2′_ = 3.7 Hz). The 6-chlorohexyl trisaccharide glycoside **29** was in turn used as a precursor to the 6-azidohexyl, 6-acetylthiohexyl and 6-thiobenzylhexyl trisaccharides **30**–**32** (Scheme 3).

Thus, nucleophilic displacement of the chloride with sodium azide or potassium thioacetate was carried out in DMF at 80 °C and provided the 6-azidohexyl and 6-acetylthiohexyl trisaccharides **30** and **31**, respectively. The introduction of the azido or thioacetyl groups into trisaccharides **30** and **31** was confirmed by HR-ESI mass spectrometry and by NMR. Indeed, the signals assigned to the methylene *CH_2_*Cl in trisaccharide **29** (^1^H NMR δ 3.50 ppm, ^13^C NMR δ 44.9 ppm) were no longer observed in trisaccharides **30** and **31**. The methylene *CH_2_*N_3_ in trisaccharide **30** gave signals at 3.20 and 54.3 ppm in the ^1^H and ^13^C NMR spectra, respectively, whereas the methylene *CH_2_*SAc in trisaccharide **31** gave signals at 2.81 and around 28.5 ppm, in the ^1^H and ^13^C NMR spectra, respectively. In addition, signals corresponding to the thioacetyl group in trisaccharide **31** were identified at 2.29 ppm and 30.6 ppm in the ^1^H and ^13^C NMR spectra, respectively. Since, as will be described below, the deprotection of trisaccharide **31** under dissolving metal conditions did not provide the desired trisaccharide **3**, the 6-benzylthiohexyl glycoside **32** was also prepared from the 6-chlorohexyl glycoside **29**. Thus, the chloride **29** was allowed to react for 16 h with excess benzylthiol (15 equiv) and sodium hydride (15 equiv) in DMF at 80 °C. These reaction conditions led to the displacement of the chloride as well as to some deacetylation of the galactose residue. Thus, after acetylation of the crude product, the desired 6-benzylthiohexyl trisaccharide **32** was isolated in excellent yield (Scheme 3). It is important to point out that the 6-chlorohexyl glycoside **29** and the 6-benzylthiohexyl glycoside **32** co-eluted on silica gel and that only a very careful analysis of the NMR data recorded for the product could confirm the absence of unreacted starting material. Indeed, the large excess of benzylthiolate used to displace the chloride in trisaccharide **29** was essential for its complete conversion to the desired 6-benzylthiohexyl glycoside **32**. The structure of trisaccharide **32** was confirmed by HR-ESI MS as well as by NMR. The methylene *CH_2_*SBn gave signals at 2.36 and 31.3 ppm, in the ^1^H and ^13^C NMR spectra, respectively whereas the *S*-benzyl group gave additional signals in the aromatic regions as well as signals corresponding to the S*CH_2_*Ph methylene around 3.70 and 36.3 ppm in the ^1^H and ^13^C NMR spectra, respectively.

**Deprotection of trisaccharides 29**–**32 under dissolving metal conditions.** As reported by Seeberger et al. [52], the removal of *O*- and *S*-benzyl groups as well as that of *O*-acetyl groups can be accomplished in one step and concurrently with the reduction of azido groups to the corresponding amines, using Birch reduction conditions. Thus we embarked on the one step deprotection of trisaccharides **29**–**32** with sodium in ammonia (Table 2).

**Table 2:** One step deprotection of trisaccharides **29**–**32**^a^.

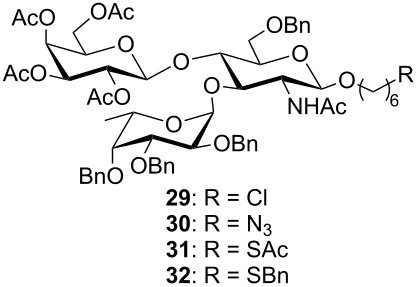			
Entry	Trisaccharide	Product	Yield (%)

1	**29**	**1**	82
2	**30**	**2**	59
3	**31**	**1**	73
4	**32**	**3**	70

^a^Reagents and conditions: Na/NH_3(_*_l_*_)_, −78 °C, 50 min.

**Scheme 4:**
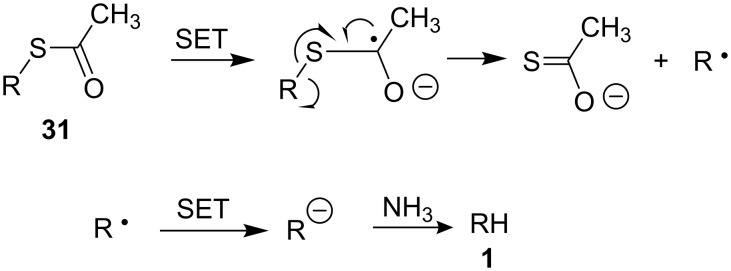
Proposed mechanism for the desulfurization of thioacetate **31** under dissolving metal conditions.

Treatment of trisaccharides **29** and **30** with sodium in liquid ammonia at −78 °C followed by neutralization of the reaction mixtures with AcOH gave the desired trisaccharides **1** and **2** (entries 1 and 2) that were isolated pure after chromatography on a Biogel P2 column eluted with water for compound **1**, and 0.05 M ammonium acetate for the 6-aminohexyl compound **2**. Whereas the structure of trisaccharide **1** was confirmed by HR-ESI mass spectrometry and NMR, the structure of the 6-aminohexyl glycoside **2** was confirmed by comparing its analytical data to that previously reported [31]. To our surprise, treatment of the 6-acetylthiohexyl trisaccharide **31** under Birch reduction conditions did not lead to the desired corresponding thiol or disulfide product but produced the hexyl glycoside **1**. The mechanism proposed to explain this reductive desulfurization is shown in Scheme 4. It involves first a single electron transfer to the thioacetyl group that is followed by the cleavage of the carbon sulfur bond giving a thioacetate salt and an alkyl radical. The alkyl radical is then converted to the corresponding anion by a second electron transfer and the resulting anion is protonated by ammonia giving trisaccharide **1**.

In contrast to the thioacetate **31**, treatment of the 6-benzylthiohexyl glycoside **32** under Birch reduction conditions did not lead to desulfurization and gave the disulfide trisaccharide dimer **3**. Under these reductive conditions, and based on the work by Seeberger et al. [52], we did not expect the formation of the disulfide dimer as the major product but rather that of the corresponding thiol. However, the structure and homogeneity of disulfide dimer **3** was unequivocally confirmed by HR-ESI mass spectrometry and NMR. Interestingly this dimer gave a well resolved ^1^H NMR spectrum in D_2_O that did not support the formation of intramolecular Le^x^–Le^x^ interactions such as those reported by de la Fuente and Penadés for a similar analogue [33]. Following published procedures, the disulfide dimer **3** will be reduced immediately prior to its conjugation to proteins [53] or immobilization on gold surface or gold nanoparticules [34].

In conclusion, we have reported above the efficient and convergent preparation of three Le^x^ derivatives (**1**–**3**) from one common protected trisaccharide (**29**). Our results seem to indicate that glycosylation at O-4 of a glucosamine monosaccharide acceptor under excess BF_3_·OEt_2_ activation at 40 °C is compatible with a chlorinated aglycon but not with an aglycon carrying an azido group. We have also established that the fully protected precursors could be deprotected in one single step to give the final target compounds using dissolving metal conditions. However, we observed that a thioacetylated derivative will undergo an undesired reductive desulfurization. This study constitutes a particularly efficient convergent preparation of analogues that can each be used for a specific biochemical application.

## Experimental

**General Methods:** ^1^H (600.14, 400.13 or 300.13 MHz) and ^13^C NMR (150.9, 100.6 or 75.5 MHz) spectra were recorded for compounds solubilized in CDCl_3_ (internal standard, for ^1^H: residual CHCl_3_ δ 7.24; for ^13^C: CDCl_3_ δ 77.0) or D_2_O [external standard 3-(trimethylsilyl)-propionic acid-*d*_4_, sodium salt (TSP) for ^1^H δ 0.00, for ^13^C δ 0.00]. Chemical shifts and coupling constants were obtained from a first-order analysis of one-dimensional spectra. Assignments of proton and carbon resonances were based on COSY and ^13^C–^1^H heteronuclear correlated experiments. Mass spectra were obtained under electron spray ionization (ESI) on a high resolution mass spectrometer. TLC were performed on precoated aluminum plates with Silica Gel 60 F254 and detected with UV light and/or charred with a solution of 10% H_2_SO_4_ in EtOH. Compounds were purified by flash chromatography with Silica Gel 60 (230–400 mesh) unless otherwise stated. Solvents were distilled and dried according to standard procedures [54], and organic solutions were dried over Na_2_SO_4_ and concentrated under reduced pressure below 40 °C. HPLC purifications were run with HPLC grade solvents.

***n*-Hexyl 2-acetamido-2-deoxy-3-*O*-(α-L-fucopyranosyl)-4-*O*-(β-D-galactopyranosyl)-β-D-glucopyranoside (1).** Trisaccharide **29** (20 mg, 0.017 mmol) or trisaccharide **31** (19 mg, 0.016 mmol) were dissolved in THF (5 mL) and liquid ammonia (20 mL) was condensed into the solution at −78 °C. Na (74 mg, 3.2 mmol) was added and the mixture was stirred for 50 min at −78 °C. The reaction was quenched with MeOH (5 mL) and the ammonia was allowed to evaporate at room temp. The remaining solution was neutralized with acetic acid (203 μL, 3.5 mmol), the solvent was evaporated and the residue was passed twice through a Biogel P2 column (100 × 1 cm) eluted with Milli-Q water to give the trisaccharide **1** (8.5 mg, 82% from **29**; 7.0 mg, 73% from **31**) as a white amorphous powder after lyophilization. [α]_D_ = −47 (*c* 0.5, MeOH), ^1^H NMR (400 MHz, D_2_O): δ 5.12 (d, 1H, *J* = 4.5 Hz, H-1′); 4.83 (m, 1H, H-5′); 4.53 (d, 1H, *J* = 7.5 Hz, H-1); 4.46 (d, 1H, *J* = 7.5 Hz, H-1″); 4.00 (dd, 1H, *J* = 12.0, 1.0 Hz, H-6a); 3.83–3.95 (m, 7H, H-2, H-3, H-4, H-6b, H-3′, H-4″, OC*H*HCH_2_); 3.78 (d, 1H, *J* = 3.0 Hz, H-4′); 3.73 (m, 2H, H-6a″, H-6b″); 3.70 (m, 1H, H-2′); 3.66 (m, 1H, H-3″); 3.60 (m, 3H, H-5, H-5″, OCH*H*CH_2_,); 3.59 (m, 1H, H-2″); 2.03 (s, 3H, CH_3_CO); 1.55 (m, 2H, OCH_2_C*H*_2_); 1.24–1.37 (m, 6H, OCH_2_CH_2_C*H*_2_C*H*_2_C*H*_2_); 1.17 (d, 3H, *J* = 6.0 Hz, H-6′); 0.88 (t, 3H, *J* = 6.6 Hz, CH_2_C*H*_3_). ^13^C-NMR (100 MHz, D_2_O): 174.17 (C=O); 101.81 (C-1″); 100.91 (C-1); 98.61 (C-1′); 75.32 (C-5); 74.94, 74.88 (C-3, C-5″); 73.36 (C-4); 72.44 (C-3″); 71.89 (C-4′); 71.02 (C-2″); 70.66 (O*C*H_2_CH_2_); 69.19 (C-3′); 68.32 (C-4″); 67.68 (C-2′); 66.68 (C-5′); 61.47 (C-6″); 59.76 (C-6); 55.84 (C-2); 30.67, 28.53, 24.77, 22.00 (OCH_2_*C*H_2_*C*H_2_*C*H_2_*C*H_2_); 22.23 (*C*H_3_CO); 15.27 (C-6′); 13.30 (CH_2_*C*H_3_). HRESIMS Calcd for C_26_H_48_NO_15_ [M+H]^+^ 614.3024, found 614.3035.

**6-Aminohexyl 2-acetamido-2-deoxy-3-*O*-(α-L-fucopyranosyl)-4-*O*-(β-D-galactopyranosyl)-β-D-glucopyranoside (2).** The azidotrisaccharide **30** (19 mg, 0.16 mmol) was deprotected in the same conditions as described above for the deprotection of trisaccharide **29**. After work up, the residue was passed twice through a Biogel P2 column (100 × 1 cm) eluted with 0.05 M ammonium acetate and after repeated lyophilization from Milli-Q water (3 × 10 mL) the known [31] trisaccharide **2** (6.5 mg, 59%) was obtained as the acetate salt in the form of a white amorphous powder. [α]_D_ = −54 (*c* 0.9, H_2_O), lit. [31]: [α]_D_ = −54.3 (*c* 1, H_2_O), ^1^H NMR (400 MHz, D_2_O): δ 5.12 (d, 1H, *J* = 4.5 Hz, H-1′); 4.83 (m, 1H, H-5′); 4.53 (d, 1H, *J* = 7.5 Hz, H-1); 4.46 (d, 1H, *J* = 7.5 Hz, H-1″); 4.00 (dd, 1H, *J* = 12.0, 1.0 Hz, H-6a); 3.83–3.95 (m, 7H, H-2, H-3, H-4, H-6b, H-3′, H-4″, OC*H*HCH_2_); 3.78 (d, 1H, *J* = 3.0 Hz, H-4′); 3.73 (m, 2H, H-6a″, H-6b″); 3.70 (m, 1H, H-2′); 3.66 (m, 1H, H-3″); 3.60 (m, 3H, H-5, H-5″, OCH*H*CH_2_); 3.59 (m, 1H, H-2″); 2.99 (t, 2H, *J* = 7.0 Hz, C*H*_2_NH_2_); 2.03, 2.01 (s, 6H, CH_3_CO); 1.57, 1.67 (m, 4H, OCH_2_C*H*_2_, C*H*_2_CH_2_NH_2_); 1.30–1.42 (m, 4H, OCH_2_CH_2_C*H*_2_C*H*_2_); 1.17 (d, 3H, *J* = 6.0 Hz, H-6′). ^13^C-NMR (100 MHz, D_2_O): 173.96 (C=O); 101.64 (C-1″); 100.81 (C-1); 98.45 (C-1′); 75.16 (C-5); 74.73 (C-3, C-5″); 73.17 (C-4); 72.28 (C-3″); 71.70 (C-4′); 70.85 (C-2″); 70.30 (O*C*H_2_CH_2_); 69.01 (C-3′); 68.15 (C-4″); 67.51 (C-2′); 66.53 (C-5′); 61.31 (C-6″); 59.57 (C-6); 55.65 (C-2); 39.21 (CH_2_NH_2_); 28.18, 26.46, 25.05, 24.45 [OCH_2_(*C*H_2_)_4_]; 22.05 (*C*H_3_CO); 15.10 (C-6′). HRESIMS calcd for C_26_H_48_N_2_O_15_ [M+H]^+^ 629.3133, found 629.3121.

**6,6′-Dithio-bis(hexan-1,6-diyl)-bis[2-acetamido-2-deoxy-3-*O*-α-L-fucopyranosyl-4-*O*-(β-D-galactopyranosyl)-β-D-glucopyranoside] (3).** The 6-benzylthiohexyl trisaccharide **32** (30 mg, 0.024 mmol) was deprotected in the same conditions as described above for the deprotection of trisaccharide **29**. After work up, the residue was passed through a Biogel P2 column eluted with water to give the trisaccharide **3** (10.6 mg, 70%) as white amorphous powder after lyophilization. [α]_D_ = −57 (*c* 0.7, MeOH), ^1^H NMR (600 MHz, D_2_O): δ 5.05 (d, 1H, *J* = 3.8 Hz, H-1′); 4.82–4.75 (m, 1H, H-5′); 4.47 (d, 1H, *J* = 7.7 Hz, H-1); 4.39 (d, 1H, *J* = 7.9 Hz, H-1″); 3.95 (d, 1H, *J* = 10.9 Hz, H-6a); 3.90–3.76 (m, 7H, H-2, H-3, H-4, H-6b, H-3′, H-4″, OC*H*HCH_2_); 3.75–3.71 (m, 1H, H-4′); 3.70–3.56 (m, 4H, H-2′H-3″, H-6a″, H-6b″); 3.55–3.49 (m, 3H, H-5, H-5″, OCH*H*CH_2_); 3.44 (t, 1H, *J* = 8.1 Hz, H-2″); 2.70 (t, 2H, *J* = 7.1 Hz, CH_2_S); 1.98 (s, 3H, CH_3_CO); 1.68–1.58 (m, 2H, SCH_2_C*H*_2_); 1.54–1.43 (m, 2H, OCH_2_C*H*_2_); 1.41–1.21 (m, 4H, OCH_2_CH_2_C*H*_2_C*H*_2_CH_2_CH_2_S); 1.12 (d, 3H, *J* = 6.6 Hz, H-6′. ^13^C-NMR (150 MHz, D_2_O): 174.09 (C=O); 101.83 (C-1″); 100.93 (C-1); 98.63 (C-1′); 75.35 (C-5); 74.96, 74.90 (C-3, C-5″); 73.41 (C-4); 72.48 (C-3″); 71.91 (C-4′); 71.05 (C-2″); 70.45 (O*C*H_2_CH_2_); 69.22 (C-3′); 68.34 (C-4″); 67.72 (C-2′); 66.71 (C-5′); 61.48 (C-6″); 59.81 (C-6); 55.86 (C-2); 38.16 (CH_2_S); 28.44, 28.30, 27.15, 24.67 (OCH_2_*C*H_2_*C*H_2_*C*H_2_*C*H_2_); 22.35 (*C*H_3_CO); 15.30 (C-6′). HRESIMS Calcd for C_59_H_92_N_2_O_30_S_2_Na [M+Na]^+^ 1311.5074, found 1311.5065.

**6-Chlorohexyl 2-acetamido-4-*O*-(2,3,4,6-tetra-*O*-acetyl-**β**-D-galactopyranosyl)-6-*O*-benzyl-3-*O*-(chloroacetyl)-2-deoxy-β-D-glucopyranoside (24).** BF_3_·Et_2_O (150 μL, 1.19 mmol, 2.0 equiv) was added to a solution of the acceptor **4** (300 mg, 0.59 mmol) and glycosyl donor **7** (1.46 g, 2.96 mmol, 5.0 equiv) [39–41] in anhyd CH_2_Cl_2_ (15 mL) at 40 °C. The reaction mixture was stirred for 1 h at 40 °C. The reaction was quenched with Et_3_N (170 μL, 1.22 mmol) and the solvent was evaporated. Flash chromatography of the residue (EtOAc–hexanes, 1:1 to 6:4) gave the disaccharide **24** (341 mg, 69%) as colorless oil. [α]_D_ = −5 (*c*1.0, CHCl_3_), ^1^H NMR (400 MHz, CDCl_3_): δ 7.40–7.26 (m, 5H, Ar); 5.72 (d, 1H, *J* = 9.2 Hz, NH); 5.24 (bd, 1H, *J* = 3.4 Hz, H-4′); 5.11 (dd, 1H, *J* = 10.0, 8.9 Hz, H-3); 4.95 (dd, 1H, *J* = 10.4, 8.0 Hz, H-2′); 4.78 (dd, 1H, *J* = 10.4, 3.5 Hz, H-3′); 4.72 (d, 1H, *J* = 12.0 Hz, PhC*H*H); 4.50–4.41 (m, 3H, H-1, PhCH*H*); 4.39 (d, 1H, *J* = 8.0 Hz, H-1′); 4.15–4.01 (m, 4H, H-6a′, H-6b′, ClCH_2_CO); 4.01–3.88 (m, 2H, H-2, H-4); 3.86–3.78 (m, 1H, OC*H*H); 3.73–3.65 (m, 2H, H-6a, H-6b); 3.62 (t, 1H, *J* = 6.5 Hz, H-5′); 3.53–3.38 (m, 4H, H-5, OCH*H*, CH_2_Cl); 2.10, 2.04, 1.93, 1.92 (4 s, 15H, CH_3_CO); 1.77–1.67 (m, 2H, C*H*_2_CH_2_Cl); 1.61–1.49 (m, 2H, OCH_2_C*H*_2_); 1.44–1.27 (m, 4H, OCH_2_CH_2_C*H*_2_C*H*_2_). ^13^C NMR (100 MHz, CDCl_3_): δ 170.30, 170.16, 169.96, 168.96, 167.34 (C=O); 137.64, 128.57, 128.07, 127.97 (Ar); 100.87 (C-1); 100.12 (C-1′); 74.48, 74.40, 74.25 (C-3, C-4, C-5); 73.62 (Ph*C*H_2_); 70.75, 70.60 (C-3′, C-5′); 69.27 (CH_2_O); 69.09 (C-2′); 67.35 (C-6); 66.81 (C-4′); 61.02 (C-6′); 53.45 (C-2); 44.97 (CH_2_Cl); 40.80 (Cl*C*H_2_CO); 32.40 (*C*H_2_CH_2_Cl); 29.21 (OCH_2_*C*H_2_); 26.44, 25.14 (OCH_2_CH_2_*C*H_2_*C*H_2_); 23.25, 20.64, 20.58, 20.48, 20.48 (*C*H_3_CO). HRESIMS Calcd for C_37_H_52_Cl_2_NO_16_ [M+H]^+^ 836.2663, found 836.2634.

**6-Chlorohexyl 2-acetamido-4-*O*-(2,3,4,6-tetra-*O*-acetyl-β-D-galactopyranosyl)-6-*O*-benzyl-2-deoxy-β-D-glucopyranoside (28).** Thiourea (162 mg, 2.13 mmol, 6.0 equiv) was added to a solution of the disaccharide **24** (298 mg, 0.356 mmol) in a mixture of pyridine and EtOH (2:1, 15 mL). The solution was stirred for 10 h at 70 °C, the solvents removed by evaporation and the residue co-concentrated with toluene (2 × 10 mL). The crude residue was dissolved in CH_2_Cl_2_ (20 mL) and washed sequentially with 2 M HCl (10 mL), saturated aq NaHCO_3_ (10 mL) and brine (10 mL). The aq phases were re-extracted with CH_2_Cl_2_ and the combined organic layers were dried and concentrated. Flash chromatography of the residue (EtOAc-hexanes, 6:4) gave the pure disaccharide **28** (165 mg, 61%) as a white amorphous powder. [α]_D_ = +1 (*c* 1.3, CHCl_3_), ^1^H NMR (400 MHz, CDCl_3_): δ 7.37–7.26 (m, 5H, Ar); 5.62 (d, 1H, *J* = 7.7 Hz, NH); 5.32 (bd, 1H, *J* = 3.4 Hz, H-4′); 5.13 (dd, 1H, *J* = 10.4, 8.0 Hz, H-2′); 4.90 (dd, 1H, *J* = 10.4, 3.4 Hz, H-3′); 4.74 (d, 1H, *J* = 8.2 Hz, H-1); 4.68 (d, 1H, *J* = 12.1 Hz, PhC*H*H); 4.47 (d, 1H, *J* = 12.1 Hz, PhCH*H*); 4.45 (d, 1H, *J* = 8.0 Hz, H-1′); 4.13–4.05 (m, 2H, H-6a′, H-6b′); 4.04–3.96 (m, 1H, H-3); 3.96–3.92 (bs, 1H, OH); 3.90–3.79 (m, 2H, H-5′, OC*H*H); 3.69–3.57 (m, 3H, H-4, H-6a, H-6b); 3.53–3.41 (m, 4H, H-5, OCH*H*, CH_2_Cl); 3.41–3.31 (m, 1H, H-2); 2.12, 2.03, 1.97, 1.95 (4 s, 15H, CH_3_CO); 1.78–1.69 (m, 2H, C*H*_2_CH_2_Cl); 1.62–1.50 (m, 2H, OCH_2_C*H*_2_); 1.46–1.29 (m, 4H, OCH_2_CH_2_C*H*_2_C*H*_2_). ^13^C NMR (100 MHz, CDCl_3_): δ 170.36, 170.07, 169.98, 169.91, 169.15 (C=O); 138.02, 128.48, 127.86, 127.78 (Ar); 101.13 (C-1′); 99.96 (C-1); 80.98 (C-4); 73.92 (C-5); 73.59 (Ph*C*H_2_); 71.34 (C-3); 71.08 (C-5′); 70.67 (C-3′); 69.31 (CH_2_O); 68.73 (C-2′); 68.05 (C-6); 66.81 (C-4′); 61.31 (C-6′); 57.05 (C-2); 44.99 (CH_2_Cl); 32.44 (*C*H_2_CH_2_Cl); 29.28 (OCH_2_*C*H_2_); 26.49, 25.19 (OCH_2_CH_2_*C*H_2_*C*H_2_); 23.58, 20.65, 20.56, 20.53, 20.47 (*C*H_3_CO). HRESIMS Calcd for C_35_H_51_ClNO_15_ [M+H]^+^ 760.2947, found 760.2928.

**6-Chlorohexyl 2-acetamido-4-*O*-(2,3,4,6-tetra-*O*-acetyl-β-D-galactopyranosyl)-6-*O*-benzyl-3-*O*-(2,3,4-tri-*O*-benzyl-α-L-fucopyranosyl)-2-deoxy-β-D-glucopyranoside (29).** A solution of the disaccharide acceptor **28** (100 mg, 0.132 mmol) and fucosyl donor **9** (189 mg, 0.395 mmol, 3.0 equiv) [42] in a mixture of CH_2_Cl_2_ and DMF (1:1, 8 mL) containing activated powdered MS 4Å (400 mg) was stirred at room temp for 30 min. Cu(II)Br_2_ (88 mg, 0.394 mmol, 3.0 equiv) and Bu_4_NBr (131 mg, 0.409 mmol, 3.1 equiv) were added and the reaction mixture was stirred for 20 h at room temp. The reaction mixture was filtered over Celite^®^ and the solids were washed with CH_2_Cl_2_ (5 mL). The filtrate was diluted with CH_2_Cl_2_ (60 mL) and washed sequentially with brine (50 mL) and saturated aq NaHCO_3_ (6 × 50 mL). The aq layers were re-extracted with CH_2_Cl_2_ (50 mL) and the combined organic layers were dried and concentrated. Flash chromatography of the residue (EtOAc–hexanes, 3:7) gave the trisaccharide **29** as colorless oil (126 mg, 81%). [α]_D_ = −19 (*c* 0.5, CHCl_3_), ^1^H NMR (600 MHz, CDCl_3_): δ 7.45–7.24 (m, 20H, Ar); 6.02 (bs, 1H, NH); 5.29 (d, 1H, *J* = 3.2 Hz, H-4″); 5.09 (d, 1H, *J* = 3.7 Hz, H-1′); 5.04 (dd, 1H, *J* = 10.4, 8.2 Hz, H-2″); 4.98 (d, 1H, *J* = 11.9 Hz, PhC*H*H); 4.92–4.85 (m, 2H, H-1, PhCH*H*); 4.85–4.79 (m, 3H, H-3″, PhC*H*_2_); 4.75 (d, 1H, *J* = 11.9 Hz, PhC*H*H); 4.71 (d, 1H, *J* = 11.8 Hz, PhCH*H*); 4.69 (d, 1H, *J* = 12.0 Hz, PhC*H*H); 4.58 (d, 1H, *J* = 8.2 Hz, H-1″); 4.45 (d, 1H, *J* = 12.0 Hz, PhCH*H*); 4.43–4.38 (m, 1H, H-5′); 4.19 (t, 1H, *J* = 7.6 Hz, H-3); 4.16–4.11 (m, 2H, H-2′, H-6a″), 4.02 (dd, 1H, *J* = 10.9, 5.9 Hz, H-6b″); 3.97–3.90 (m, 2H, H-4, H-3′); 3.85–3.74 (m, 3H, H-6a, H-6b, OC*H*HCH_2_); 3.68 (s, 1H, H-4′); 3.58 (t, 1H, *J* = 7.0 Hz, H-5″); 3.55–3.45 (m, 4H, H-2, H-5, CH_2_Cl); 3.44–3.38 (m, 1H, OCH*H*CH_2_); 2.03, 2.02, 1.98, 1.84, 1.78 (5 s, 15H, CH_3_CO); 1.76–1.69 (m, 2H, C*H*_2_CH_2_Cl); 1.58–1.47 (m, 2H, OCH_2_C*H*_2_); 1.44–1.27 (m, 4H, OCH_2_CH_2_C*H*_2_C*H*_2_); 1.18 (d, 3H, *J* = 6.5 Hz, H-6′). ^13^C NMR (150 MHz, CDCl_3_): δ 170.45, 170.00, 169.88, 169.85, 169.21 (C=O); 138.72, 138.68, 138.47, 137.78, 128.46, 128.40, 128.30, 128.24, 128.11, 127.90, 127.73, 127.69, 127.61, 127.49, 127.28, 127.00 (Ar); 99.38, 99.34 (C-1, C-1″); 97.33 (C-1′); 79.82 (C-3′); 76.79 (C-4′); 76.31 (C-2′); 74.23 (Ph*C*H_2_); 74.21 (C-5); 74.03 (C-4); 73.58, 73.35 (Ph*C*H_2_); 73.32 (C-3); 72.38 (Ph*C*H_2_); 70.52 (C-3″); 70.27 (C-5″); 69.23 (O*C*H_2_CH_2_); 68.73 (C-2″); 68.33 (C-6); 66.61 (C-4″); 66.39 (C-5′); 60.22 (C-6″); 56.20 (C-2); 44.92 (CH_2_Cl); 32.38 (*C*H_2_CH_2_Cl); 29.12 (OCH_2_*C*H_2_); 26.45, 25.08 (OCH_2_CH_2_*C*H_2_*C*H_2_); 22.95, 20.65, 20.52, 20.49, 20.44 (*C*H_3_CO). HRESIMS Calcd for C_62_H_79_ClNO_19_ [M+H]^+^ 1176.4935, found 1176.4933.

**6-Azidohexyl 2-acetamido-4-*O*-(2,3,4,6-tetra-*O*-acetyl-β-D-galactopyranosyl)-6-*O*-benzyl-3-*O*-(2,3,4-tri-*O*-benzyl-α-L-fucopyranosyl)-2-deoxy-β-D-glucopyranoside (30).** NaN_3_ (17 mg, 0.26 mmol, 8.2 equiv) was added to a solution of the trisaccharide **29** (38 mg, 0.032 mmol) in anhyd DMF (2.5 mL) and the reaction mixture was heated at 80 °C for 36 h. The solvent was evaporated, the residue was dissolved in CH_2_Cl_2_ (50 mL) and washed with water (2 × 10 mL). The aq phases were re-extracted with CH_2_Cl_2_ and the combined organic layers were dried and concentrated. Flash chromatography of the residue (EtOAc-hexanes, 6:4) afforded the trisaccharide **30** as a clear glass (36 mg, 95%). [α]_D_ = −47 (*c* 1.0, CH_2_Cl_2_), ^1^H NMR (400 MHz, CDCl_3_): δ 7.41–7.20 (m, 20H, Ar); 5.81 (d, 1H, *J* = 7.6 Hz, NH); 5.26 (d, 1H, *J* = 3.0 Hz, H-4″); 5.06 (d, 1H, *J* = 3.8 Hz, H-1′), 5.00 (dd, 1H, *J* = 10.4, 8.2 Hz, H-2″); 4.94 (d, 1H, *J* = 11.8 Hz, PhC*H*H); 4.91–4.83 (m, 2H, H-1, PhCH*H*); 4.82–4.74 (m, 3H, H-3″, PhC*H*_2_); 4.73–4.62 (m, 3H, PhC*H*_2_, PhC*H*H); 4.54 (d, 1H, *J* = 8.1 Hz, H-1″); 4.43–4.34 (m, 2H, H-5′, PhCH*H*); 4.20–4.05 (m, 3H, H-3, H-2′, H-6a″); 3.98 (dd, 1H, *J* = 10.8, 5.9 Hz, H-6b″); 3.95–3.87 (m, 2H, H-4, H-3′); 3.83–3.68 (m, 3H, H-6a, H-6b, OC*H*HCH_2_); 3.65 (d, 1H, *J* = 1.4 Hz, H-4′); 3.57–3.44 (m, 2H, H-5, H-5″); 3.43–3.31 (m, 2H, H-2, OCH*H*CH_2_); 3.20 (t, 2H, *J* = 6.9 Hz, CH_2_N_3_); 1.99, 1.98, 1.93, 1.89, 1.70 (5s, 15H, CH_3_CO); 1.58–1.43 (m, 4H, C*H*_2_CH_2_N_3_, OCH_2_C*H*_2_); 1.33–1.21 (m, 4H, OCH_2_CH_2_C*H*_2_C*H*_2_); 1.15 (d, 3H, *J* = 6.5 Hz, H-6′). ^13^C NMR (100 MHz, CDCl_3_): δ 170.09, 170.06, 169.96, 169.91, 169.20 (C=O); 138.88, 138.77, 138.57, 137.89, 128.53, 128.47, 128.38, 128.32, 128.19, 127.97, 127.77, 127.75, 127.66, 127.56, 127.36, 127.08 (Ar); 99.45 (C-1, C-1″); 97.44 (C-1′); 79.97 (C-3′); 76.90 (C-4′); 76.42 (C-2′); 74.19 (C-5, C-4); 73.70 (Ph*C*H_2_); 73.43 (C-3, Ph*C*H_2_); 72.46 (Ph*C*H_2_); 70.62 (C-3″); 70.35 (C-5″); 69.29 (O*C*H_2_CH_2_); 68.80 (C-2″); 68.43 (C-6); 66.69 (C-4″); 66.42 (C-5′); 60.28 (C-6″); 56.60 (C-2); 51.32 (CH_2_N_3_); 29.23, 28.73, 26.41, 25.43 (OCH_2_*C*H_2_*C*H_2_*C*H_2_*C*H_2_CH_2_N_3_); 23.16, 20.70, 20.59, 20.56, 20.51 (*C*H_3_CO); 16.71 (C-6′). HRESIMS Calcd for C_62_H_79_N_4_O_19_ [M+H]^+^ 1183.5339, found 1183.5325.

**6-Acetylthiohexyl 2-acetamido-4-*O*-(2,3,4,6-tetra-*O*-acetyl-β-D-galactopyranosyl)-6-*O*-benzyl-3-*O*-(2,3,4-tri-*O*-benzyl-α-L-fucopyranosyl)-2-deoxy-β-D-glucopyranoside (31).** KSC(O)CH_3_ (26 mg, 0.22 mmol, 10 equiv) was added to a solution of the trisaccharide **29** (27 mg, 0.023 mmol) in anhyd DMF (1.5 mL) and the reaction mixture was heated at 80 °C for 16 h. Work up and chromatography (EtOAc–hexanes, 6:4), as described above for compound **30** gave the trisaccharide **31** as colorless glass (19 mg, 70%). [α]_D_ = −43 (*c* 0.7, CH_2_Cl_2_), ^1^H NMR (400 MHz, CDCl_3_): δ 7.40–7.20 (m, 20H, Ar); 5.85 (d, 1H, *J* = 7.7 Hz, NH); 5.25 (d, 1H, *J* = 3.0 Hz, H-4″); 5.07 (d, 1H, *J* = 3.8 Hz, H-1′), 5.01 (dd, 1H, *J* = 10.4, 8.2 Hz, H-2″); 4.98 (d, 1H, *J* = 11.8 Hz, PhC*H*H); 4.90–4.83 (m, 2H, H-1, PhCH*H*); 4.82–4.74 (m, 3H, H-3″, PhC*H*_2_); 4.74–4.62 (m, 3H, PhC*H*_2_, PhC*H*H); 4.54 (d, 1H, *J* = 8.2 Hz, H-1″); 4.44–4.35 (m, 2H, H-5′, PhC*H*H); 4.18 (t, 1H, *J* = 7.7 Hz, H-3); 4.14–4.05 (m, 2H, H-2′, H-6a″); 3.97 (dd, 1H, *J* = 10.8, 5.9 Hz, H-6b″); 3.94–3.85 (m, 2H, H-4, H-3′); 3.83–3.68 (m, 3H, H-6a, H-6b, OC*H*HCH_2_); 3.65 (d, 1H, *J* = 2.7 Hz, H-4′); 3.53 (t, 1H, *J* = 6.8 Hz, H-5″); 3.50–3.44 (m, 1H, H-5); 3.43–3.30 (m, 2H, H-2, OCH*H*CH_2_); 2.81 (t, 2H, *J* = 7.2 Hz, CH_2_S); 2.29 (s, 3H, SCOCH_3_); 1.99, 1.97, 1.93, 1.89, 1.71 (5s, 15H, CH_3_CO); 1.59–1.41 (m, 4H, C*H*_2_CH_2_S, OCH_2_C*H*_2_); 1.33–1.20 (m, 4H, OCH_2_CH_2_C*H*_2_C*H*_2_); 1.15 (d, 3H, *J* = 6.5 Hz, H-6′). ^13^C NMR (100 MHz, CDCl_3_): δ 195.99, 170.16, 170.06, 169.97, 169.92, 169.19 (C=O); 138.86, 138.80, 138.59, 137.90, 128.53, 128.47, 128.37, 128.31, 128.19, 127.97, 127.80, 127.77, 127.67, 127.56, 127.34, 127.09 (Ar); 99.45, 99.42 (C-1, C-1″); 97.40 (C-1′); 79.94 (C-3′); 76.94 (C-4′); 76.40 (C-2′); 74.33 (C-5); 74.31 (Ph*C*H_2_); 74.21 (C-4); 73.66, 73.44 (Ph*C*H_2_); 73.36 (C-3); 72.49 (Ph*C*H_2_); 70.64 (C-3″); 70.34 (C-5″); 69.39 (O*C*H_2_CH_2_); 68.81 (C-2″); 68.40 (C-6); 66.71 (C-4″); 66.40 (C-5′); 60.29 (C-6″); 56.60 (C-2); 30.61 (SCO*C*H_3_); 29.40, 29.40, 29.20, 28.96, 28.43, 25.36 (O*C*H_2_*C*H_2_*C*H_2_*C*H_2_*C*H_2_*C*H_2_S); 23.18, 20.70, 20.59, 20.56, 20.51 (*C*H_3_CO); 16.71 (C-6′). HRESIMS Calcd for C_64_H_82_NO_20_S [M+H]^+^ 1216.5151, found 1216.5151.

**6-Benzylthiohexyl 2-acetamido-4-*O*-(2,3,4,6-tetra-*O*-acetyl-β-D-galactopyranosyl)-6-*O*-benzyl-3-*O*-(2,3,4-tri-*O*-benzyl-α-L-fucopyranosyl)-2-deoxy-β-D-glucopyranoside (32).** PhCH_2_SH (60 µL, 0.44 mmol, 15 equiv) and NaH (21 mg, 0.44 mmol, 15 equiv) were added to a solution of the trisaccharide **29** (36 mg, 0.030 mmol) in anhyd DMF (3.0 mL) at room temp. After 10 min the reaction mixture was heated to 80 °C for 16 h, the solvent was evaporated and the residue was dissolved in Ac_2_O and pyridine (5 ml, 1:1). After 18 h the reaction mixture was co-concentrated with toluene (3 × 20 ml), the residue was dissolved in CH_2_Cl_2_ (30 mL) and the solution was washed with water (2 × 10 mL). The aq phases were re-extracted with CH_2_Cl_2_ and the combined organic layers were dried and concentrated. Flash chromatography of the residue (EtOAc–hexanes, 1:1) gave the trisaccharide **32** (35.6 mg, 94%) as a white solid. [α]_D_ = −28 (*c* 1.0, CH_2_Cl_2_), ^1^H NMR (400 MHz, CDCl_3_): δ 7.44–7.13 (m, 25H, Ar); 5.79 (d, 1H, *J* = 7.6 Hz, NH); 5.25 (d, 1H, *J* = 3.0 Hz, H-4″); 5.05 (d, 1H, *J* = 3.8 Hz, H-1′), 5.00 (dd, 1H, *J* = 10.5, 8.2 Hz, H-2″); 4.94 (d, 1H, *J* = 11.8 Hz, PhC*H*H); 4.90–4.74 (m, 5H, H-1, H-3″, PhC*H*_2_, PhCH*H*); 4.73–4.64 (m, 3H, PhC*H*H, PhC*H*_2_); 4.54 (d, 1H, *J* = 8.2 Hz, H-1″); 4.43–4.36 (m, 2H, H-5′, PhC*H*H); 4.17 (t, 1H, *J* = 7.7 Hz, H-3); 4.14–4.06 (m, 2H, H-2′, H-6a″); 4.01–3.95 (m, 1H, H-6b″); 3.93–3.87 (m, 2H, H-4, H-3′); 3.79–3.64 (m, 6H, H-6a, H-6b, H-4′, SC*H*_2_Ph, OC*H*HCH_2_); 3.56–3.45 (m, 2H, H-5, H-5″); 3.41–3.31 (m, 2H, H-2, OCH*H*CH_2_); 2.36 (t, 2H, *J* = 7.3 Hz, C*H*_2_SBn); 1.99, 1.98, 1.94, 1.90, 1.70 (5s, 15H, CH_3_CO); 1.52–1.42 (m, 4H, C*H*_2_CH_2_S, OCH_2_C*H*_2_); 1.33–1.28 (m, 4H, OCH_2_CH_2_C*H*_2_C*H*_2_); 1.15 (d, 3H, *J* = 6.4 Hz, H-6′). ^13^C NMR (150 MHz, CDCl_3_): δ 170.14, 170.10, 170.02, 169.96, 169.20 (C=O); 140.06, 138.91, 138.81, 138.61, 137.93, 137.63, 130.14, 129.02, 128.97, 128.53, 128.49, 128.30, 128.00, 127.89, 127.73, 127.60, 127.39, 126.89 (Ar); 99.48, 99.41 (C-1, C-1″); 97.47 (C-1′); 80.05 (C-3′); 76.86 (C-4′); 76.42 (C-2′); 74.34 (C-5); 74.31 (Ph*C*H_2_); 74.25 (C-4); 73.77, 73.46 (Ph*C*H_2_); 73.35 (C-3); 72.47 (Ph*C*H_2_); 70.67 (C-3″); 70.33 (C-5″); 69.49 (O*C*H_2_CH_2_); 68.81 (C-2″); 68.39 (C-6); 66.70 (C-4″); 66.41 (C-5′); 60.28 (C-6″); 52.07 (C-2); 36.30 (S-*C*H_2_Ph); 31.30 (*C*H_2_SBn); 29.29, 29.13, 29.06, 25.53 (OCH_2_*C*H_2_*C*H_2_*C*H_2_*C*H_2_CH_2_S); 23.23, 20.75, 20.64, 20.61, 20.57 (*C*H_3_CO); 16.76 (C-6′). HRESIMS Calcd for C_69_H_86_NO_19_S [M+H]^+^ 1264.5515, found 1264.5509.

## Supporting Information

File 1 Experimental procedures and characteristics for compounds **4**–**6**, **8**, **11**, **12**, **14**–**19**, **21**–**23**, **25**–**27**.

File 2 ^1^H and ^13^C NMR spectra for compounds **1**–**6**, **8**, **11**, **12**, **16**–**19**, **21**–**32**, ^1^H NMR data for known compounds **14**, **15**.
