# Supplementary material for: Convergent syntheses of LeX analogues
Source: Beilstein J Org Chem. 2010 Feb 22;6:17. doi: 10.3762/bjoc.6.17 (PMC2870943; doi:10.3762/bjoc.6.17)
Supplement: File 1 — Experimental procedures and characteristics for compounds 4–6, 8, 11, 12, 14–19, 21–23, 25–27. [file Beilstein_J_Org_Chem-06-17-s001.pdf]

Supporting Information File 1:

Experimental Procedures and Characteristics for Compounds **4-6**, **8**, **11**, **12**, **14-19**, **21-23**, **25-27**.

for

Convergent Syntheses of Le^X Analogues

*An Wang, Jenifer Hendel and France-Isabelle Auzanneau**

Department of Chemistry, University of Guelph, Guelph, Ontario, N1G 2W1, Canada.

*Corresponding author. Email: fauzanne@uoguelph.ca

6-Chlorohexyl 2-acetamido-6-*O*-benzyl-3-*O*-chloroacetyl-2-deoxy-*β*-D-glucopyranoside (4). A soln of the benzylidene acetal **12** (400 mg, 0.79 mmol) and $NaCNBH_3$ (600 mg, 9.5 mmol, 12 equiv) in anhyd THF (20 mL) containing activated MS 3 Å (2.0 g) and methyl orange indicator (1 mg) was stirred for 30 min at 0 °C under N_2. A soln of HCl in Et_2O (2.0 M, 4.8 mL, 9.6 mmol 12 equiv) was added drop wise to the reaction mixture at 0 °C until the soln turned pink. The reaction was then allowed to proceed under stirring for 1 h at room temp and the reaction mixture was then filtered over Celite®. The solids were washed with THF (10 mL) and the combined filtrates were concentrated. The residue was dissolved in CH_2Cl_2 (20 mL) and washed sequentially with saturated aq $NaHCO_3$ (2 x 10 mL) and brine (10 mL). The aq phases were re-extracted with CH_2Cl_2 (10 mL) and the combined organic layers were dried and concentrated. Flash chromatography of the residue ($CHCl_3$-MeOH, 20:1) gave the desired alcohol **4** (300 mg, 75%) as colorless oil. $[\alpha]_D$ = -17 (c 1.0, MeOH), ^{1}H NMR (300 MHz, $CDCl_3$): δ 7.40-7.20 (m, 5H, Ar); 5.93 (d, 1H, J = 9.1 Hz, NH); 5.18 (dd, 1H, J = 10.4, 9.2 Hz, H-3); 4.64-4.48 (m, 3H, H-1, $PhCH_2$); 4.10 (s, 2H, $ClCH_2CO$); 3.95-3.66 (m, 5H, H-2, H-4, H-6a, H-6b, OC*H*H); 3.60-

3.38 (m, 4H, H-5, OCH*H*, CH_2Cl); 3.35-3.26 (broad, 1H, OH); 1.91 (s, 3H, CH_3CO); 1.78-1.64 (m, 2H, C*H*$_2$CH$_2$Cl); 1.62-1.48 (m, 2H, OCH$_2$C*H*$_2$); 1.48-1.20 (m, 4H, OCH$_2$CH$_2$C*H*$_2$C*H*$_2$). ^{13}C NMR (75 MHz, $CDCl_3$): δ 170.51, 168.34 (C=O); 137.50, 128.48, 127.91, 127.72 (Ar); 100.69 (C-1); 77.43 (C-3); 73.79 (C-5); 73.75 (Ph*C*H$_2$); 70.74 (C-4); 70.32 (C-6); 69.40 (CH_2O); 54.11 (C-2); 44.99 (CH_2Cl); 40.93 (Cl*C*H$_2$CO); 32.44 (*C*H$_2$CH$_2$Cl); 29.24 (OCH$_2$*C*H$_2$); 26.46, 25.13 (OCH$_2$CH$_2$*C*H$_2$*C*H$_2$); 23.26 (*C*H$_3$CO). HRESIMS Calcd for $C_{23}H_{34}Cl_2NO_7$ $[M+H]^+$ 506.1712, found 506.1730

6-Azidohexyl 2-acetamido-6-*O*-benzyl-3-*O*-chloroacetyl-2-deoxy-*β*-D-glucopyranoside (5). A soln of the chloroacetate **19** (200 mg, 0.391 mmol) in anhyd CH_2Cl_2 (10 mL) containing activated MS 4 Å (500 mg, 50 mg/mL) was stirred for 1 h under N_2. Et_3SiH (188 µL, 1.18 mmol, 3.0 equiv) and TfOH (118 µL, 1.33 mmol, 3.4 equiv) were added to the reaction mixture at -30 ^{o}C and the soln was stirred for 30 min. The reaction was quenched with Et_3N (200 µL) and MeOH (500 µL), the mixture diluted with CH_2Cl_2 (20 mL) and filtered through Celite®. The solids were washed with CH_2Cl_2 and the combined filtrate and washing were concentrated to dryness. Flash chromatography of the residue ($CHCl_3$-MeOH, 50:1) gave alcohol **5** (119 mg, 59%) as a white amorphous powder. $[\alpha]_D$ = -1 (*c* 0.9, MeOH), ^{1}H NMR (400 MHz, $CDCl_3$): δ 7.35-7.25 (m, 5H, Ar); 6.09 (d, 1H, *J* = 9.1 Hz, NH); 5.19 (dd, 1H, *J* = 10.5, 9.1 Hz, H-3); 4.60-4.50 (m, 3H, H-1, PhC*H*$_2$); 4.15-4.08 (m, 2H, ClCH$_2$CO); 3.91-3.66 (m, 5H, H-2, H-4, H-6a, H-6b, OC*H*H); 3.60-3.52 (m, 1H, H-5); 3.47-3.36 (m, 1H, OCH*H*); 3.21 (t, 2H, *J* = 6.9 Hz, CH_2N_3); 1.91 (s, 3H, CH_3CO); 1.62-1.57 (m, 4H, C*H*$_2$CH$_2$N$_3$, OCH$_2$C*H*$_2$); 1.39-1.26 (m, 4H, OCH$_2$CH$_2$C*H*$_2$C*H*$_2$). ^{13}C NMR (100 MHz, $CDCl_3$): δ 170.57, 168.39 (C=O); 137.52, 128.45, 127.87, 127.67 (Ar); 100.64 (C-1); 77.44 (C-3); 73.82 (C-5); 73.69 (Ph*C*H$_2$); 70.61 (C-4); 70.23 (C-6); 69.39 (CH_2O); 54.05 (C-2); 51.28 (CH_2N_3); 40.97 (Cl*C*H$_2$CO); 29.22, 28.69 (*C*H$_2$CH$_2$N$_3$,

OCH_2CH_2); 26.33, 25.39 ($OCH_2CH_2CH_2CH_2$); 23.21 (CH_3CO). HRESIMS Calcd for $C_{23}H_{34}ClN_4O_7$ $[M+H]^+$ 513.2216, found 513.2091.

6-Azidohexyl 6-*O*-benzyl-3-*O*-chloroacetyl-2-deoxy-2-phthalimido-*β*-D-glucopyranoside (6). A soln of benzylidene acetal **17** (2.74 g, 4.57 mmol) and $NaCNBH_3$ (4.38 g, 69.8 mmol, 15 equiv) in anhyd THF (150 mL) containing activated MS 3 Å (7.5 g) and methyl orange indicator (2 mg) was cooled to 0 °C and stirred for 30 min under N_2. A soln of HCl in Et_2O (2.0 M, 35 mL, 70 mmol, 15 equiv) was added drop wise to the reaction mixture stirred at 0 °C. The reaction was allowed to proceed for 1 h at room temp and work up was carried out as described above for the preparation of acceptor **4**. Flash chromatography of the crude product (EtOAc-hexanes, 4:6) gave alcohol **6** (1.83 g, 67%) as colorless oil. $[\alpha]_D$ = -22 (*c* 1.3, $CHCl_3$), ^1H NMR (400 MHz, $CDCl_3$): δ 7.86-7.78, 7.74-7.66, 7.38-7.22 (m, 9H, Ar); 5.71 (t, 1H, *J* = 10.5 Hz, H-3); 5.31 (d, 1H, *J* = 8.4 Hz, H-1); 4.66-4.54 (2d, 2H, *J* = 12.0 Hz, $PhCH_2$); 4.23 (dd, 1H, *J* = 10.8, 8.4 Hz, H-2); 3.88-3.68 (m, 7H, H-4, H-5, H-6a, H-6b, $ClCH_2CO$, OC*H*H); 3.44-3.35 (m, 1H, OCH*H*); 3.12-3.07 (broad, 1H, OH); 3.01 (t, 2H, *J* = 6.8 Hz, CH_2N_3); 1.49-1.31 (m, 2H, $CH_2CH_2N_3$); 1.30-1.18 (m, 2H, OCH_2CH_2); 1.17-1.00 (m, 4H, $OCH_2CH_2CH_2CH_2$). ^{13}C NMR (100 MHz, $CDCl_3$): δ 168.25, 167.39 (C=O); 137.47, 134.30, 131.33, 128.48, 127.91, 127.74, 123.52 (Ar); 97.95 (C-1); 75.10 (C-3); 73.76 ($PhCH_2$); 73.74 (C-5); 71.48 (C-4); 70.16 (C-6); 69.63 (CH_2O); 54.38 (C-2); 51.07 (CH_2N_3); 40.43 ($ClCH_2CO$); 29.03 ($CH_2CH_2N_3$); 28.52 (OCH_2CH_2); 26.13, 25.33 ($OCH_2CH_2CH_2CH_2$). HRESIMS Calcd for $C_{29}H_{37}ClN_5O_8$ $[M+NH_4]^+$ 618.2331, found 618.2330.

2,4,6-tri-*O*-acetyl-3-*O*-chloroacetyl-*α*-D-galactopyranosyl trichloroacetimidate (8). NIS (4.95 g, 22.0 mmol, 1.17 equiv) and TfOH (0.33 mL, 3.7 mmol, 0.2 equiv) were added to a soln of the chloroacetate **23** (9.25 g, 18.9 mmol) in a mixture of MeCN (250 mL) and H_2O (3 mL) at

0 °C. The reaction mixture was stirred for 30 min at 0 °C, the reaction quenched with Et_3N (0.52 mL, 3.7 mmol) and the solvent evaporated. The residue was dissolved in CH_2Cl_2 (300 mL) and washed with a 20% w/w soln of aq $Na_2S_2O_3$ (100 mL). The aq phase was re-extracted with CH_2Cl_2 and the combined organic layers were dried and concentrated. Flash chromatography of the residue (EtOAc-hexanes, 1:1) gave the corresponding hemiacetal (5.0 g, 13 mmol). Trichloroacetonitrile (7.9 mL, 79 mmol, 6.1 equiv) and DBU (0.78 mL, 5.2 mmol, 0.4 equiv) were added under N_2 to a soln of the hemiacetal in anhyd CH_2Cl_2 (50 mL) stirred at room temp. The reaction mixture was stirred for 1 h, concentrated and flash chromatography of the residue (EtOAc-hexanes, 4:6 with 0.1% Et_3N) gave trichloroacetimidate **8** as a yellow amorphous powder (4.72 g, 48% for two steps). $[\alpha]_D$ = +90 (*c* 1.0, $CHCl_3$), ^{1}H NMR (400 MHz, $CDCl_3$): δ 8.66 (s, 1H, NH); 6.59 (d, 1H, *J* = 3.6 Hz, H-1); 5.53 (d, 1H, *J* = 3.2 Hz, H-4); 5.47 (dd, 1H, *J* = 10.8, 3.2 Hz, H-3); 5.38 (dd, 1H, *J* = 10.8, 3.6 Hz, H-2); 4.42 (t, 1H, *J* = 6.7 Hz, H-5); 4.15 (dd, 1H, *J* = 11.3, 6.7 Hz, H-6a); 4.07 (dd, 1H, *J* = 11.3, 6.7 Hz, H-6b); 3.97 (s, 2H, $ClCH_2CO$); 2.14, 2.00 (2s, 9H, CH_3CO). ^{13}C NMR (100 MHz, $CDCl_3$): δ 170.31, 170.23, 169.94, 166.46 (C=O); 160.81 (C=NH); 93.38 (C-1); 90.66 (CCl_3); 69.28 (C-3); 68.88 (C-5); 67.09 (C-4); 66.63 (C-2); 61.05 (C-6); 40.40 ($ClCH_2CO$); 20.60, 20.48, 20.47 (CH_3CO). HRESIMS Calcd for $C_{16}H_{19}O_{10}NCl_4Na$ $[M+Na]^+$ 547.9661, found 547.9664.

6-Chlorohexyl 2-acetamido-4,6-*O*-benzylidene-2-deoxy-*β*-D-glucopyranoside (11). Sodium (106 mg, 4.6 mmol, 0.50 equiv) was added to a soln of peracetate **10** (4.3 g, 9.2 mmol) [1] in anhyd MeOH (200 mL) and the reaction mixture was stirred for 4 h at room temp. The solution was de-ionized with Dowex® 50WX8100 (H^+ resin), the resin filtered off over glass wool and washed with MeOH (100 mL). The combined filtrate and washing were concentrated and the residue was dried under high vacuum giving the corresponding triol (3.1 g, 99%) as an

amorphous powder. Benzaldehyde dimethyl acetal (2.1 mL, 14 mmol, 1.5 equiv) and CSA (300 mg, 3.5 mg/mL) were added under N_2 to a stirred suspension of the triol (3.1 g, 9.1 mmol) in anhyd CH_3CN (85 mL). The reaction was allowed to proceed for 30 min, quenched with Et_3N (0.18 mL) and the mixture was cooled to 0 ^{o}C to promote precipitation. The benzylidene acetal **11** (3.04 g, 77%) was collected by filtration and isolated as a white amorphous powder. $[\alpha]_D$ = -42 (*c* 0.6, MeOH), ^{1}H NMR (400 MHz, $CDCl_3$): δ 7.50-7.44, 7.39-7.30 (m, 5H, Ar); 5.72 (d, 1H, *J* = 5.2 Hz, NH); 5.53 (s, 1H, PhC*H*); 4.71 (d, 1H, *J* = 8.3 Hz, H-1) 4.32 (dd, 1H, *J* = 10.2, 4.9 Hz, H-6a); 4.15 (t, 1H, *J* = 9.5 Hz, H-3); 3.90-3.82 (m, 1H, OC*H*H); 3.77 (t, 1H, *J* = 9.5 Hz, H-6b); 3.56-3.37 (m, 6H, H-2, H-4, H-5, CH_2Cl, OCH*H*); 2.03 (s, 3H, CH_3CO); 1.81-1.72 (m, 2H, CH_2CH_2Cl); 1.64-1.54 (m, 2H, OCH_2CH_2); 1.50-1.31 (m, 4H, $OCH_2CH_2CH_2CH_2$). ^{13}C NMR (100 MHz, $CDCl_3$): δ 171.71 (C=O); 137.03, 129.20, 128.28, 126.32 (Ar); 101.93 (Ph*C*H); 100.55 (C-1); 81.69 (C-4); 71.15 (C-3); 69.77 (CH_2O); 68.62 (C-6); 66.28 (C-5); 59.26 (C-2); 44.98 (CH_2Cl); 32.43 (CH_2CH_2Cl); 29.33 (OCH_2CH_2); 26.51, 25.23 ($OCH_2CH_2CH_2CH_2$); 23.68 (CH_3CO). HRESIMS Calcd for $C_{21}H_{31}ClNO_6$ $[M+H]^+$ 428.1840, found 428.1841.

6-Chlorohexyl 2-acetamido-4,6-*O*-benzylidene-3-*O*-chloroacetyl-2-deoxy-*β*-D-glucopyranoside (12). Chloroacetyl chloride (158 μL, 1.98 mmol, 1.7 equiv) was added at room temp to a stirred soln of the benzylidene acetal **11** (500 mg, 1.17 mmol) in anhyd CH_2Cl_2 (10 mL) containing pyridine (241 μL, 3.0 mmol, 2.6 equiv). The reaction mixture was stirred for 30 min at room temp, the solvent evaporated and the residue co-concentrated with toluene (2 x 10 mL). The crude residue was dissolved in CH_2Cl_2 (20 mL) and washed sequentially with 2 M HCl (10 mL), saturated aq $NaHCO_3$ (10 mL) and water (10 mL). The aq phases were re-extracted with CH_2Cl_2 and the combined organic layers were dried and concentrated. Precipitation of the residue from EtOH (10 mL) gave chloroacetate **12** (450 mg, 77%) as a white amorphous powder.

$[\alpha]_D$ = -41 (*c* 0.3, MeOH), ^{1}H NMR (400 MHz, $CDCl_3$): δ 7.47-7.27 (m, 5H, Ar); 6.04 (d, 1H, *J* = 9.4 Hz, NH); 5.46 (s, 1H, PhC*H*); 5.38 (t, 1H, *J* = 9.9 Hz, H-3); 4.43 (d, 1H, *J* = 8.3 Hz, H-1); 4.28 (dd, 1H, *J* = 10.4, 4.9 Hz, H-6a); 4.14-4.02 (m, 3H, H-2, $ClCH_2CO$); 3.80-3.65 (m, 3H, H-4, H-6b, OC*H*H); 3.58-3.45 (m, 3H, H-5, CH_2Cl); 3.34-3.25 (m, 1H, OCH*H*); 1.95 (s, 3H, CH_3CO); 1.80-1.68 (m, 2H, CH_2CH_2Cl); 1.60-1.46 (m, 2H, OCH_2CH_2); 1.46-1.25 (m, 4H, $OCH_2CH_2CH_2CH_2$). ^{13}C NMR (100 MHz, $CDCl_3$): δ 170.31, 168.08 (C=O); 136.83, 129.11, 128.25, 125.99 (Ar); 101.80 (C-1); 101.29 (Ph*C*H); 78.56 (C-4); 74.08 (C-3); 69.86 (CH_2O); 68.54 (C-6); 65.99 (C-5); 54.31 (C-2); 45.00 (CH_2Cl); 40.83 ($ClCH_2CO$); 32.44 (CH_2CH_2Cl); 29.22 (OCH_2CH_2); 26.47, 25.08 ($OCH_2CH_2CH_2CH_2$); 23.27 (CH_3CO). HRESIMS Calcd for $C_{23}H_{32}Cl_2NO_7$ $[M+H]^+$ 504.1556, found 504.1562.

6-Chlorohexyl 3,4,6-tri-*O*-acetyl-2-deoxy-2-phthalimido-*β*-D-glucopyranoside (14). $BF_3 \cdot Et_2O$ (132 μL, 1.05 mmol, 5.0 equiv) was added to a soln of tetraacetate **13** (100 mg, 0.209 mmol) [2] and chlorohexanol (112 μL, 0.839 mmol, 4.0 equiv) in anhyd CH_2Cl_2 (5 mL). The sealed reaction mixture was then heated to 50 °C in an oil bath for 1 h (Method A) or irradiated in a microwave reactor for 5 min at 50 °C (Method B). The reaction mixtures for Method A and B were then handled identically. They were diluted with CH_2Cl_2 (20 mL) and washed sequentially with saturated aq $NaHCO_3$ (2 x 25 mL) and brine (25 mL). The aq phases were re-extracted with CH_2Cl_2 and the combined organic layers were dried and concentrated. The residues were dissolved in a mixture of Ac_2O and pyridine (1:1, 1 mL) and stirred at room temp for 2 h. The solvents were evaporated and the residues were co-concentrated with toluene (2 x 5 mL). Flash chromatography (EtOAc-hexanes, 4:6) gave the known [3] product **14** as colorless oil in both cases (Method A: 95 mg, 82%, Method B: 98 mg, 85%). $[\alpha]_D$ = +17 (*c* 1.0, $CHCl_3$), lit. [43]: $[\alpha]_D$ = +19 (*c* 1.7, $CHCl_3$), ^{1}H NMR (300 MHz, $CDCl_3$): δ 7.84-7.79 (m, 2H, Ar); 7.74-7.70

(m, 2H, Ar); 5.75 (dd, 1H, *J* = 10.8, 9.0 Hz, H-3); 5.31 (d, 1H, *J* = 8.5 Hz, H-1); 5.13 (dd, 1H, *J* = 10.0, 9.2 Hz, H-4); 4.29 (dd, 1H, *J* = 12.2, 4.6 Hz, H-6a); 4.27 (dd, 1H, *J* = 10.8, 8.5 Hz, H-2); 4.13 (dd, 1H, *J* = 12.2, 2.4 Hz, H-6b); 3.82 (m, 2H, C*H*HO, H-5); 3.39 (m, 1H, CH*H*O); 3.27 (t, 2H, *J* = 6.9 Hz, CH_2Cl); 2.07, 1.99, 1.82 (3s, 9H, CH_3CO); 1.39 (m, 4H, OCH_2CH_2, CH_2CH_2Cl); 1.11 (m, 4H, $OCH_2CH_2CH_2CH_2$).

6-Azidohexyl 3,4,6-tri-*O*-acetyl-2-deoxy-2-phthalimido-*β*-D-glucopyranoside (15). NaN_3 (1.49 g, 22.9 mmol, 3.0 equiv) was added to a soln of chloride **14** (4.24 g, 7.65 mmol) in anhyd DMF (250 mL) at room temp. The mixture was stirred overnight at 80 °C, the solvent evaporated and the residue dissolved in CH_2Cl_2 (250 mL) and washed with water (2 x 150 mL). The aq phases were re-extracted with CH_2Cl_2 (100 mL) and the combined organic layers were dried (Na_2SO_4) and concentrated to give the known [4] compound **15** (4.29 g, 99%) as a colorless oil. $[\alpha]_D$ = +22 (*c* 1.1, $CHCl_3$), lit. [44]: $[\alpha]_D$ = +19 (*c* 1, $CHCl_3$), ^{1}H NMR (400 MHz, $CDCl_3$): 7.89-7.75 (m, 4H, Ar); 5.79 (dd, 1H, *J* = 10.8, 9.1 Hz, H-3); 5.36 (d, 1H, *J* = 8.5 Hz, H-1); 5.17 (dd, 1H, *J* = 9.8, 9.1 Hz, H-4); 4.33 (dd, 1H, *J* = 12.3, 4.7 Hz, H-6a); 4.31 (dd, 1H, *J* = 10.8, 8.5 Hz, H-2); 4.17 (dd, 1H, *J* = 12.3, 2.3 Hz, H-6b); 3.85-3.76 (m, 2H, H-5, OC*H*H); 3.44 (m, 1H, OCH*H*); 3.06 (t, 2H, *J* = 6.9 Hz, CH_2N_3); 1.86, 2.03, 2.11 (3s, 9H, CH_3CO); 1.45-1.43 (m, 2H, $CH_2CH_2N_3$); 1.29-1.23 (m, 2H, OCH_2CH_2); 1.17-1.12 (m, 4H, $OCH_2CH_2CH_2CH_2$).

6-Azidohexyl 4,6-*O*-benzylidene-2-deoxy-2-phthalimido-*β*-D-glucopyranoside (16). NaOMe (2.16 g, 40 mmol, 2.5 equiv) was added to a soln of compound **15** (8.96 g, 16.0 mmol) in anhyd MeOH (200 mL) and the mixture was stirred at room temp for 4 h. The solution was de-ionized with Dowex® 50WX8100 (H^+ resin), the resin filtered off over glass wool and washed with MeOH (100 mL). The combined filtrate and washing were concentrated and the residue was

dried under high vacuum to give the corresponding triol (6.5 g, 94%) as an amorphous powder. Benzaldehyde dimethyl acetal (3.4 mL, 23 mmol, 1.5 equiv) and CSA (1.00 g, 4 mg/mL) were added under N_2 to a stirred suspension of the triol (6.5 g, 15 mmol) in anhyd MeCN (250 mL). The soln was stirred at room temp for 4 h and additional benzaldehyde dimethyl acetal (1.1 mL, 7.3 mmol, 0.5 equiv) was added. After 3 h the reaction was quenched with Et_3N (0.6 mL, 4.3 mmol) and the solvents was evaporated. Flash chromatography of the residue (EtOAc-hexanes, 3:7) gave benzylidene acetal **16** as colorless oil (4.73 g, 56%). $[\alpha]_D$ = -31 (*c* 1.0, $CHCl_3$), ^{1}H NMR (400 MHz, $CDCl_3$): δ 7.87-7.80, 7.74-7.67, 7.51-7.44, 7.39-7.31 (m, 9H, Ar); 5.55 (s, 1H, PhC*H*); 5.22 (d, 1H, *J* = 8.5 Hz, H-1); 4.59 (dd, 1H, *J* = 10.6, 8.6 Hz, H-3); 4.36 (dd, 1H, *J* = 10.3, 4.3 Hz, H-6a); 4.21 (dd, 1H, *J* = 10.6, 8.5 Hz, H-2); 3.85-3.76 (m, 2H, H-6b, OC*H*H); 3.64-3.53 (m, 2H, H-4, H-5); 3.43-3.35 (m, 1H, OCH*H*); 3.02 (t, 2H, *J* = 6.9 Hz, CH_2N_3); 1.47-1.30 (m, 2H, $CH_2CH_2N_3$); 1.30-1.16 (m, 2H, OCH_2CH_2); 1.16-1.01 (m, 4H, $OCH_2CH_2CH_2CH_2$). ^{13}C NMR (100 MHz, $CDCl_3$): δ 168.28 (C=O); 136.94, 134.16, 131.56, 129.29, 128.32, 126.26, 123.41 (Ar); 101.88 (Ph*C*H); 98.86 (C-1); 82.22 (C-4); 69.74 (CH_2O); 68.65 (C-6); 68.55 (C-3); 66.11 (C-5); 56.59 (C-2); 51.07 (CH_2N_3); 29.06 ($CH_2CH_2N_3$); 28.51 (OCH_2CH_2); 26.12, 25.30 ($OCH_2CH_2CH_2CH_2$). HRESIMS Calcd for $C_{27}H_{30}N_4NaO_7$ $[M+Na]^+$ 545.2012, found 545.2007.

6-Azidohexyl 4,6-*O*-benzylidene-3-*O*-chloroacetyl-2-deoxy-2-phthalimido-*β*-D-glucopyranoside (17). Chloroacetyl chloride (1.43 mL, 18.0 mmol, 2.0 equiv) was added at room temp to a soln of benzylidene acetal **16** (4.7 g, 9.0 mmol) in anhyd CH_2Cl_2 (150 mL) and pyridine (14 mL, 181 mmol, 20 equiv). The reaction mixture was stirred for 2 h, the solvent evaporated, the residue co-concentrated with toluene (2 x 100 mL), dissolved in CH_2Cl_2 (150 mL) and washed with 2 M HCl (100 mL), saturated aq $NaHCO_3$ (100 mL) and water (100 mL). The aq phases were re-extracted with CH_2Cl_2 and the combined organic layers were dried and

concentrated. Flash chromatography (EtOAc-hexanes, 3:7) gave chloroacetate **17** as colorless oil (3.02 g, 56%). $[\alpha]_D$ = -26 (*c* 1.0, $CHCl_3$), ^{1}H NMR (400 MHz, $CDCl_3$): δ 7.89-7.81, 7.77-7.70, 7.46-7.44, 7.38-7.30 (m, 9H, Ar); 5.94 (dd, 1H, *J* = 10.4, 9.0 Hz, H-3); 5.52 (s, 1H, PhC*H*); 5.40 (d, 1H, *J* = 8.4 Hz, H-1); 4.41 (dd, 1H, *J* = 10.4, 4.7 Hz, H-6a); 4.32 (dd, 1H, *J* = 10.4, 8.4 Hz, H-2); 3.89 (m, 2H, $ClCH_2CO$); 3.87-3.69 (m, 4H, H-4, H-5, H-6b, OC*H*H); 3.46-3.37 (m, 1H, OCH*H*); 3.02 (t, 2H, *J* = 6.9 Hz, CH_2N_3); 1.49-1.30 (m, 2H, $C\mathit{H}_2CH_2N_3$); 1.30-1.16 (m, 2H, $OCH_2C\mathit{H}_2$); 1.16-1.01 (m, 4H, $OCH_2CH_2C\mathit{H}_2C\mathit{H}_2$). ^{13}C NMR (100 MHz, $CDCl_3$): δ 167.94, 167.37, 166.67 (C=O); 136.65, 134.36, 131.24, 129.17, 128.22, 126.14, 123.58 (Ar); 101.59 (Ph*C*H); 98.59 (C-1); 79.14 (C-4); 71.44 (C-3); 69.98 (CH_2O); 68.57 (C-6); 66.06 (C-5); 56.01 (C-2); 51.03 (CH_2N_3); 40.30 ($Cl\mathit{C}H_2CO$); 29.01 ($\mathit{C}H_2CH_2N_3$); 28.49 ($OCH_2\mathit{C}H_2$); 26.09, 25.27 ($OCH_2CH_2\mathit{C}H_2\mathit{C}H_2$). HRESIMS Calcd for $C_{29}H_{31}ClN_4NaO_8$ $[M+Na]^+$ 621.1728, found 621.1732

6-Azidohexyl 2-acetamido-4,6-*O*-benzylidene-2-deoxy-*β*-D-glucopyranoside (18). Ethylenediamine (5.7 mL, 85 mmol, 10 equiv) was added to a soln of phthalimido **15** (4.8 g, 8.6 mmol) in anhyd EtOH (250 mL) and the reaction mixture was refluxed overnight. The solvent was evaporated, the residue co-concentrated with toluene (3 x 50 mL), dissolved in Ac_2O and pyridine (1:1, 50 mL) and the mixture was stirred overnight at room temp. The solvents were evaporated, the residue co-concentrated with toluene (3 x 50 mL) and the crude triacetate was dissolved in anhyd MeOH (100 mL). Na (0.46 g, 20 mmol) was added to the reaction mixture at room temp, the solution was stirred at room temp for 4 h and de-ionized with Dowex® 50WX8100 (H^+ resin). The resin was filtered off over glass wool, washed with MeOH (100 mL) and the combined filtrate and washing were concentrated and the residue dried under high vacuum. Benzaldehyde dimethyl acetal (1.9 mL, 12.7 mmol, 1.5 equiv) and CSA (1.00 g, 4 mg/mL) were added to a suspension stirred under N_2 of the resulting crude triol in anhyd MeCN

(250 mL). The soln was stirred at room temp for 1 h and additional benzaldehyde dimethyl acetal (0.6 mL, 4 mmol, 0.5 equiv) was added. The soln was stirred for another 30 min at room temp and the reaction quenched with Et_3N (0.6 mL, 4.3 mmol). Half of the solvent was evaporated and the remaining solution was placed at -24 ^{o}C for 1 h to promote precipitation of benzylidene **18**. The white solid was collected by filtration and washed with cold MeCN (20 mL). The combined filtrate and washing was concentrated to approximately 50 mL and placed at -24 ^{o}C for 1 h to provide a second crop of precipitate. The combined solids were dried under high vacuum yielding benzylidene acetal **18** (2.42 g, 66% for four steps). $[\alpha]_D$ = -42 (*c* 0.5, DMF), ^{1}H NMR (400 MHz, $CDCl_3$): δ 7.52-7.40, 7.39-7.30 (m, 5H, Ar); 5.78 (d, 1H, *J* = 6.0 Hz, NH); 5.51 (s, 1H, PhC*H*); 4.68 (d, 1H, *J* = 8.3 Hz, H-1); 4.31 (dd, 1H, *J* = 10.5, 4.8 Hz, H-6a); 4.12 (t, 1H, *J* = 9.3 Hz, H-3); 3.85 (m, 1H, OC*H*H); 3.76 (t, 1H, *J* = 10.1 Hz, H-6b); 3.56-3.38 (m, 4H, H-2, H-4, H-5, OCH*H*); 3.25 (t, 2H, *J* = 6.8 Hz, CH_2N_3); 2.02 (s, 3H, CH_3CO); 1.65-1.50 (m, 4H, $CH_2CH_2N_3$, OCH_2CH_2); 1.44-1.28 (m, 4H, $OCH_2CH_2CH_2CH_2$). ^{13}C NMR (100 MHz, $CDCl_3$): δ 171.70 (C=O); 137.03, 129.19, 128.27, 126.32 (Ar); 101.92 (Ph*C*H); 100.60 (C-1); 81.65 (C-4); 71.06 (C-3); 69.77 (CH_2O); 68.60 (C-6); 66.25 (C-5); 59.02 (C-2); 51.31 (CH_2N_3); 29.33, 28.73 ($CH_2CH_2N_3$, OCH_2CH_2); 26.40, 25.48 ($OCH_2CH_2CH_2CH_2$); 23.61 (CH_3CO). HRESIMS Calcd for $C_{21}H_{31}O_6N_4$ $[M + H]^+$ 435.2244, found 435.2223.

6-Azidohexyl 2-acetamido-4,6-*O*-benzylidene-3-*O*-chloroacetyl-2-deoxy-*β*-D-glucopyranoside (19). Chloroacetyl chloride (0.75 mL, 9.4 mmol, 2.0 equiv) was added to a soln of benzylidene acetal **18** (2.04 g, 4.7 mmol) in anhyd CH_2Cl_2 (200 mL) and pyridine (7.6 mL, 94 mmol, 20 equiv) at 0 ^{o}C. The reaction mixture was stirred for 30 min, the solvent evaporated, the residue co-concentrated with toluene (2 x 100 mL) and dissolved in CH_2Cl_2 (150 mL). The organic soln was washed sequentially with 2 M HCl (100 mL), saturated aq $NaHCO_3$

(100 mL), water (100 mL) and the aq phases re-extracted with CH_2Cl_2. The combined organic layers were dried and concentrated and triturating the residue in EtOH (50 mL) gave chloroacetate **19** (1.70 g, 71%) as a white amorphous powder. $[\alpha]_D$ = -65 (*c* 0.6, DMF), ^1H NMR (400 MHz, $CDCl_3$): δ 7.43-7.37, 7.36-7.30 (m, 5H, Ar); 6.05 (d, 1H, *J* = 9.4 Hz, NH); 5.46 (s, 1H, PhC*H*); 5.38 (t, 1H, *J* = 10.0 Hz, H-3); 4.46 (d, 1H, *J* = 8.4 Hz, H-1); 4.29 (dd, 1H, *J* = 10.5, 5.0 Hz, H-6a); 4.15-4.03 (m, 3H, H-2, $ClCH_2CO$); 3.80-3.66 (m, 3H, H-4, H-6b, OC*H*H); 3.56-3.48 (m, 1H, H-5); 3.36-3.28 (m, 1H, OCH*H*); 3.24 (t, 2H, *J* = 6.9 Hz, CH_2N_3); 1.95 (s, 3H, CH_3CO); 1.60-1.47 (m, 4H, $CH_2CH_2N_3$, OCH_2CH_2); 1.40-1.27 (m, 4H, $OCH_2CH_2CH_2CH_2$). ^{13}C NMR (100 MHz, $CDCl_3$): δ 170.35, 168.03 (C=O); 136.82, 129.12, 128.25, 126.00 (Ar); 101.77 (C-1); 101.30 (Ph*C*H); 78.55 (C-4); 74.04 (C-3); 69.87 (CH_2O); 68.54 (C-6); 66.01 (C-5); 54.37 (C-2); 51.33 (CH_2N_3); 40.82 ($ClCH_2CO$); 29.25, 28.73 ($CH_2CH_2N_3$, OCH_2CH_2); 26.37, 25.38 ($OCH_2CH_2CH_2CH_2$); 23.25 (CH_3CO). HRESIMS Calcd for $C_{23}H_{32}ClN_4O_7$ $[M+H]^+$ 511.1960, found 511.1958.

Tolyl 2,6-di-*O*-acetyl- 3,4-*O*-isopropylidene-*β*-1-thio-D-galactopyranoside (21). A soln of the known [5] isopropylidene acetal **20** (2.26 g, 6.92 mmol) in a mixture of Ac_2O and pyridine (1:1, 30 mL) was stirred for 18 h at room temp. The solvents were evaporated and the residue was co-concentrated with toluene (2 x 30 mL) and dissolved in CH_2Cl_2 (200 mL). The organic soln was washed sequentially with 2 M HCl (100 mL), saturated aq $NaHCO_3$ (100 mL) and water (100 mL) and the aq phases were re-extracted with CH_2Cl_2. The combined organic layers were dried (Na_2SO_4), concentrated and the residue dried under high vacuum giving diacetate **21** as a white amorphous powder (2.66 g, 95%). $[\alpha]_D$ = +41 (*c* 1.0, $CHCl_3$), ^1H NMR ($CDCl_3$): δ 7.39-7.32, 7.09-7.01 (m, 4H, Ar); 5.02-4.94 (m, 1H, H-2); 4.49 (d, 1H, *J* = 10.0 Hz, H-1); 4.36-4.26 (m, 2H, H-6a, H-6b); 4.18-4.11 (m, 2H, H-3, H-4); 3.95-3.88 (m, 1H, H-5); 2.28 (s, 3H, PhCH_3); 2.09,

2.04 (2 s, 6H, CH_3CO); 1.47, 1.28 (2 s, 6H, $C(CH_3)_2$). ^{13}C NMR ($CDCl_3$): δ 170.58, 169.51 (C=O); 137.84, 132.40, 129.57, 129.44 (Ar); 110.70 (*C*(CH_3)$_2$); 85.92 (C-1); 76.94 (C-3); 74.01 (C-5); 73.40 (C-4); 71.22 (C-2); 63.48 (C-6); 27.42, 26.20 (C(*C*H_3)$_2$); 20.98, 20.91, 20.68 (*C*H_3CO×2, Ph*C*H_3). HRESIMS Calcd for $C_{20}H_{30}O_7NS$ $[M+NH_4]^+$ 428.1743, found 428.1753.

Tolyl 2,6-di-*O*-acetyl-*β*-1-thio-D-galactopyranoside (22). A soln of the isopropylidene acetal **21** (2.66 g, 6.48 mmol) in 90% AcOH (50 mL) was stirred at 70 °C for 4 h. The solvent was evaporated and the residue co-concentrated with toluene (2 x 25 mL) and dried under high vacuum giving diol **22** as a white amorphous powder (2.30 g, 96%). $[\alpha]_D$ = +26 (*c* 0.9, $CHCl_3$), ^1H NMR ($CDCl_3$): δ 7.40-7.34, 7.12-7.06 (m, 4H, Ar); 4.95 (t, 1H, *J*=9.7 Hz, H-2); 4.53 (d, 1H, *J* = 10.0 Hz, H-1); 4.34 (dd, 1H, *J* = 11.6, 5.8 Hz, H-6a); 4.28 (dd, 1H, *J* = 11.6, 6.9 Hz, H-6b); 3.92 (d, 1H, *J* = 3.1 Hz, H-4); 3.62-3.70 (m, 2H, H-3, H-5); 2.31 (s, 3H, PhC*H*$_3$); 2.14, 2.06 (2 s, 6H, CH_3CO). ^{13}C NMR ($CDCl_3$): δ 171.23, 171.10 (C=O); 138.23, 132.93, 129.63, 128.82 (Ar); 86.29 (C-1); 75.88 (C-5); 73.43 (C-3); 71.13 (C-2); 68.81 (C-4); 62.83 (C-6); 21.13, 21.07 (*C*H_3CO); 20.82 (Ph*C*H_3). HRESIMS Calcd for $C_{17}H_{26}O_7$ NS $[M+NH_4]^+$ 388.1430, found 388.1449.

Tolyl 2,4,6-tri-*O*-acetyl-3-*O*-chloroacetyl-*β*-1-thio-D-galactopyranoside (23). Trimethylorthoacetate (1.3 mL, 10 mmol, 1.5 equiv) and CSA (0.8 g, 4 mg/mL) were added under N_2 to a soln of diol **22** (2.53 g, 6.83 mmol) in anhyd MeCN (200 mL). The soln was stirred at room temp for 30 min, H_2O (2 mL) was added and after 5 min the reaction was quenched with Et_3N (0.5 mL) and the solvent was evaporated. Chloroacetyl chloride (1.09 mL, 13.7 mmol, 2.0 equiv) was added to a mixture of the crude triacetate in anhyd CH_2Cl_2 (200 mL) and pyridine (11.0 mL, 137 mmol, 20.0 equiv) at 0 °C. The reaction mixture was stirred for 15 min, the

solvent evaporated, the residue co-concentrated with toluene (2 x 50 mL) and dissolved in CH_2Cl_2 (400 mL). The organic soln was washed sequentially with 2 M HCl (200 mL), saturated aq $NaHCO_3$ (200 mL) and brine (200 mL) and the aq phases were re-extracted with CH_2Cl_2. The combined organic layers were dried and concentrated. Flash chromatography (EtOAc-hexanes, 35:65) of the residue gave chloroacetate **23** as a white amorphous powder (2.32 g, 70% for three steps). $[\alpha]_D$ = +11 (*c* 1.0, $CHCl_3$), ^1H NMR (400 MHz, $CDCl_3$): δ 7.41-7.35, 7.13-7.07 (m, 4H, Ar); 5.38 (d, 1H, *J* = 3.3 Hz, H-4); 5.20 (t, 1H, *J* = 9.9 Hz, H-2); 5.07 (dd, 1H, *J* = 9.9, 3.3 Hz, H-3); 4.63 (d, 1H, *J* = 9.9 Hz, H-1); 4.17 (dd, 1H, *J* = 11.3, 6.8 Hz, H-6a); 4.09 (dd, 1H, *J* = 11.3, 6.7 Hz, H-6b); 3.96-3.87 (m, 3H, H-5, $ClCH_2CO$); 2.31 (s, 3H, $PhCH_3$); 2.08, 2.07, 2.01 (3 s, 9H, CH_3CO). ^{13}C NMR (100 MHz, $CDCl_3$): δ 170.33, 170.27, 169.29, 166.55 (C=O); 138.52, 133.17, 129.59, 128.29 (Ar); 86.74 (C-1); 74.14 (C-5); 73.72 (C-3); 66.94, 66.90 (C-2, C-4); 61.35 (C-6); 40.33 ($ClCH_2CO$); 21.10, 20.77 (CH_3CO); 20.59 ($PhCH_3$). HRESIMS Calcd for $C_{21}H_{29}O_9NSCl$ $[M+NH_4]^+$ 506.1252, found 506.1241.

6-Chlorohexyl 2-acetamido-4-*O*-(2,4,6-tri-*O*-acetyl-3-*O*-chloroacetyl-*β*-D-galactopyranosyl)-6-*O*-benzyl-3-*O*-chloroacetyl-2-deoxy-*β*-D-glucopyranoside (25). $BF_3{\cdot}OEt_2$ (38 μL, 0.30 mmol, 2.0 equiv) was added to a soln of the acceptor **4** (76 mg, 0.15 mmol) and donor **8** (396 mg, 0.75 mmol, 5.0 equiv) in anhyd CH_2Cl_2 (4 mL) at 40 °C. The reaction mixture was stirred for 1 h at 40 °C, the reaction quenched with Et_3N (50 μL, 0.35 mmol) and the solvent evaporated. Flash chromatography (EtOAc-hexanes, 1:1) gave the disaccharide **25** (94 mg, 72%) as colorless oil. $[\alpha]_D$ = -7 (*c* 0.8 $CHCl_3$), ^1H NMR (400 MHz, $CDCl_3$): δ 7.40-7.27 (m, 5H, Ar); 5.84 (d, 1H, *J* = 9.2 Hz, NH); 5.23 (bd, 1H, *J* = 3.4 Hz, H-4'); 5.13 (dd, 1H, *J* = 10.1, 8.9 Hz, H-3); 4.93 (dd, 1H, *J* = 10.4, 8.0 Hz, H-2'); 4.80 (dd, 1H, *J* = 10.4, 3.4 Hz, H-3'); 4.75 (d, 1H, *J* = 12.0 Hz, PhC*H*H); 4.47 (d, 1H, *J* = 7.9 Hz, H-1); 4.40 (d,

1H, *J* = 12.0 Hz, PhCH*H*); 4.34 (d, 1H, *J* = 7.9 Hz, H-1'); 4.15-4.00 (m, 4H, H-6a', H-6b', $ClCH_2CO$); 3.99-3.88 (m, 4H, H-2, H-4, $ClCH_2CO$); 3.87-3.78 (m, 1H, OC*H*H); 3.74-3.63 (m, 2H, H-6a, H-6b); 3.60-3.53 (m, 1H, H-5'); 3.52-3.38 (m, 4H, H-5, OCH*H*, CH_2Cl); 2.10, 2.05, 1.93, 1.92 (4 s, 12H, CH_3CO); 1.77-1.67 (m, 2H, CH_2CH_2Cl); 1.60-1.49 (m, 2H, OCH_2CH_2); 1.44-1.27 (m, 4H, $OCH_2CH_2CH_2CH_2$). ^{13}C NMR (100 MHz, $CDCl_3$): δ 170.44, 170.36, 170.30, 169.33, 167.63. 160.51 (C=O); 137.54, 128.63, 128.20, 128.15 (Ar); 100.84 (C-1); 99.79 (C-1'); 74.42, 74.13 (C-3, C-4, C-5); 73.63 (Ph*C*H_2); 72.32, (C-3'); 70.50 (C-5'); 69.43 (CH_2O); 68.80 (C-2'); 67.23 (C-6); 66.62 (C-4'); 60.84 (C-6'); 53.46 (C-2); 44.96 (CH_2Cl); 40.84, 40.30 (Cl*C*H_2CO); 32.39 (*C*H_2CH_2Cl); 29.16 (OCH_2*C*H_2); 26.43, 25.10 (OCH_2CH_2*C*H_2*C*H_2); 23.24, 20.63, 20.58, 20.58 (*C*H_3CO). HRESIMS Calcd for $C_{37}H_{51}Cl_3NO_{16}$ $[M+H]^+$ 870.2273, found 870.2234

6-Azidohexyl 2-acetamido-4-*O*-(2,4,6-tri-*O*-acetyl-3-*O*-chloroacetyl-*β*-D-galactopyranosyl)-6-*O*-benzyl-3-*O*-chloroacetyl-2-deoxy-*β*-D-glucopyranoside (26). Glycosylation of acceptor **5** (37 mg, 0.072 mmol) with donor **8** (190 mg, 0.36 mmol, 5.0 equiv) as well as work up of the reaction mixture were carried out as described above for the preparation of disaccharide **25**. Flash chromatography (EtOAc-hexanes, 1:1 then $CHCl_3$-MeOH, 9:1) then reverse-phase HPLC (gradient, MeCN-H_2O, 1:1 to 100% MeCN) gave semi-pure disaccharide **26** (17 mg, 27%) as colorless oil. $[\alpha]_D$ = -7 (*c* 0.8, $CHCl_3$), ^{1}H NMR (400 MHz, $CDCl_3$): δ 7.40-7.27 (m, 5H, Ar); 5.58 (d, 1H, *J* = 9.1 Hz, NH); 5.24 (d, 1H, *J* = 3.4 Hz, H-4'), 5.12 (dd, 1H, *J* = 10.1, 8.8 Hz, H-3); 4.97 (dd, 1H, *J* = 10.4, 8.0 Hz, H-2'); 4.80 (dd, 1H, *J* = 10.4, 3.5 Hz, H-3'); 4.76 (d, 1H, *J* = 12.0 Hz, PhC*H*H); 4.48 (d, 1H, *J* = 7.9 Hz, H-1); 4.42 (d, 1H, *J* = 12.0 Hz, PhCH*H*); 4.38 (d, 1H, *J* = 7.9 Hz, H-1'); 4.16-4.00 (m, 4H, H-6a', H-6b', $ClCH_2CO$); 4.00-3.89 (m, 4H, H-2, H-4, $ClCH_2CO$); 3.88-3.78 (m, 1H, OC*H*H); 3.74-3.66 (m, 2H, H-6a, H-6b); 3.63-3.57 (m, 1H, H-5');

3.50-3.38 (m, 2H, H-5, OCH*H*); 3.23 (t, 2H, *J* = 6.9 Hz, CH_2N_3); 2.12, 2.06, 1.94, 1.93 (4 s, 12H, CH_3CO); 1.62-1.45 (m, 4H, C*H*$_2$$CH_2N_3$, OCH_2C*H*$_2$); 1.44-1.20 (m, 4H, OCH_2CH_2C*H*$_2$C*H*$_2$). ^{13}C NMR (100 MHz, $CDCl_3$): δ 170.43, 170.31, 170.26, 168.94, 167.31. 166.59 (C=O); 137.63, 128.66, 128.21, 128.14 (Ar); 100.87 (C-1); 99.99 (C-1'); 74.39, 74.33 (C-3, C-4, C-5); 73.68 (Ph*C*H_2); 72.56, (C-3'); 70.47 (C-5'); 69.35 (CH_2O); 68.81 (C-2'); 67.30 (C-6); 66.63 (C-4'); 60.90 (C-6'); 53.68 (C-2); 51.32 (CH_2N_3); 40.78, 40.35 (Cl*C*H_2CO); 29.25, 28.74 (*C*$H_2CH_2N_3$, OCH_2*C*H_2); 26.38, 25.46 (OCH_2CH_2*C*H_2*C*H_2); 23.29, 20.67, 20.65, 20.65 (*C*H_3CO). HRESIMS Calcd for $C_{37}H_{51}Cl_2N_4O_{16}$ $[M+H]^+$ 877.2677, found 877.2669.

6-Azidohexyl 4-*O*-(2,4,6-tri-*O*-acetyl-3-*O*-chloroacetyl-*β*-D-galactopyranosyl)-6-*O*-benzyl-3-*O*-chloroacetyl-2-deoxy-2-phthalimido-*β*-D-glucopyranoside (27). Glycosylation of acceptor **6** (30 mg, 0.050 mmol) with donor **8** (132 mg, 0.25 mmol, 5.0 equiv) as well as work up of the reaction mixture were carried out as described above for the preparation of disaccharide **25**. Flash chromatography (EtOAc-hexanes, 4:6) then reverse-phase HPLC (MeCN-H_2O, 7:3) gave disaccharide **27** (5.4 mg, 11%) as colorless oil. $[\alpha]_D$ = +13 (*c* 0.3, $CHCl_3$), ^{1}H NMR (400 MHz, $CDCl_3$): δ 7.87-7.79, 7.77-7.69, 7.43-7.30 (m, 9H, Ar); 5.75 (dd, 1H, *J* = 10.9, 9.0 Hz, H-3); 5.28 (d, 1H, *J* = 8.4 Hz, H-1); 5.21 (d, 1H, *J* = 2.7 Hz, H-4'); 4.97 (dd, 1H, *J* = 10.3, 8.0 Hz, H-2'); 4.83 (d, 1H, *J* = 12.0 Hz, PhC*H*H); 4.78 (dd, 1H, *J* = 10.3, 3.5 Hz, H-3'); 4.44 (d, 1H, *J* = 12.0 Hz, PhCH*H*); 4.00 (d, 1H, *J* = 8.0 Hz, H-1'); 4.25 (dd, 1H, *J* = 10.9, 8.4 Hz, H-2); 4.11-3.98 (m, 3H, H-4, H-6a', H-6b'); 3.97-3.86 (m, 4H, 2 x $ClCH_2CO$); 3.86-3.73 (m, 3H, H-6a, H-6b, OC*H*H); 3.65-3.58 (m, 1H, H-5); 3.55 (dd, 1H, *J* = 7.1, 6.5 Hz, H-5'); 3.43-3.35 (m, 1H, OCH*H*); 3.00 (t, 2H, *J* = 6.8 Hz, CH_2N_3); 2.10, 2.08, 1.96 (3 s, 9H, CH_3CO); 1.50-1.32 (m, 2H, C*H*$_2$$CH_2N_3$); 1.31-1.20 (m, 2H, OCH_2C*H*$_2$); 1.19-1.01 (m, 4H, OCH_2CH_2C*H*$_2$C*H*$_2$). ^{13}C NMR (100 MHz, $CDCl_3$): δ 170.47, 170.30, 168.70, 166.63, 166.50 (C=O); 137.64, 134.33, 131.37,

128.72, 128.29, 128.26, 123.61 (Ar); 100.00 (C-1'); 98.17 (C-1); 75.12 (C-4); 74.35 (C-5); 73.74 (Ph*C*H_2); 72.71, 72.62 (C-3, C-3'); 70.40 (C-5'); 69.74 (CH_2O); 68.82 (C-2'); 67.13 (C-6); 66.62 (C-4'); 60.87 (C-6'); 54.55 (C-2); 51.12 (CH_2N_3); 40.55, 40.37 (Cl*C*H_2CO); 29.03 (*C*$H_2CH_2N_3$); 28.52 (OCH_2*C*H_2); 26.13, 25.33 (OCH_2CH_2*C*H_2*C*H_2); 20.69, 20.68, 20.66 (*C*H_3CO). HRESIMS Calcd for $C_{43}H_{54}Cl_2N_5O_{17}$ $[M+NH_4]^+$ 982.2892, found 982.2922.

References

1. Zemlyakov, A. E. *Chem. Nat. Comp.* **1998**, *34*, 80-85.

2. Lemieux, R. U. ; Takeda, T. ; Chung, B. Y. **1976**, *A.C.S. Symp. Ser. 39*, 90-115.

3. Nitz, M.; Bundle, D. R. *J. Org. Chem.* **2000**, *65*, 3064-3073.

4. Joosten, J. A. F.; Kamerling, J. P.; Vliegenthart, J. F. G. *Carbohydr. Res.* **2003**, *338*, 2611-2627.

5. Choudhury, A. K.; Roy, N. *J. Carbohydr. Chem.* **1997**, *16*, 1363-1371.
