# Supplementary material for: Convergent syntheses of LeX analogues
Source: Beilstein J Org Chem. 2010 Feb 22;6:17. doi: 10.3762/bjoc.6.17 (PMC2870943; doi:10.3762/bjoc.6.17)

Supporting Information File 2:

- **^{1}H- and ^{13}C NMR spectra for compounds 1-6, 8, 11, 12, 16-19, 21-32**
- **^{1}H NMR spectra for known compounds 14, 15**

for

Convergent Syntheses of Le^X Analogues

*An Wang, Jenifer Hendel and France-Isabelle Auzanneau**

Department of Chemistry, University of Guelph, Guelph, Ontario, N1G 2W1, Canada.

* Corresponding author. Email: fauzanne@uoguelph.ca

Compound **1**, 400 MHz, D_2O

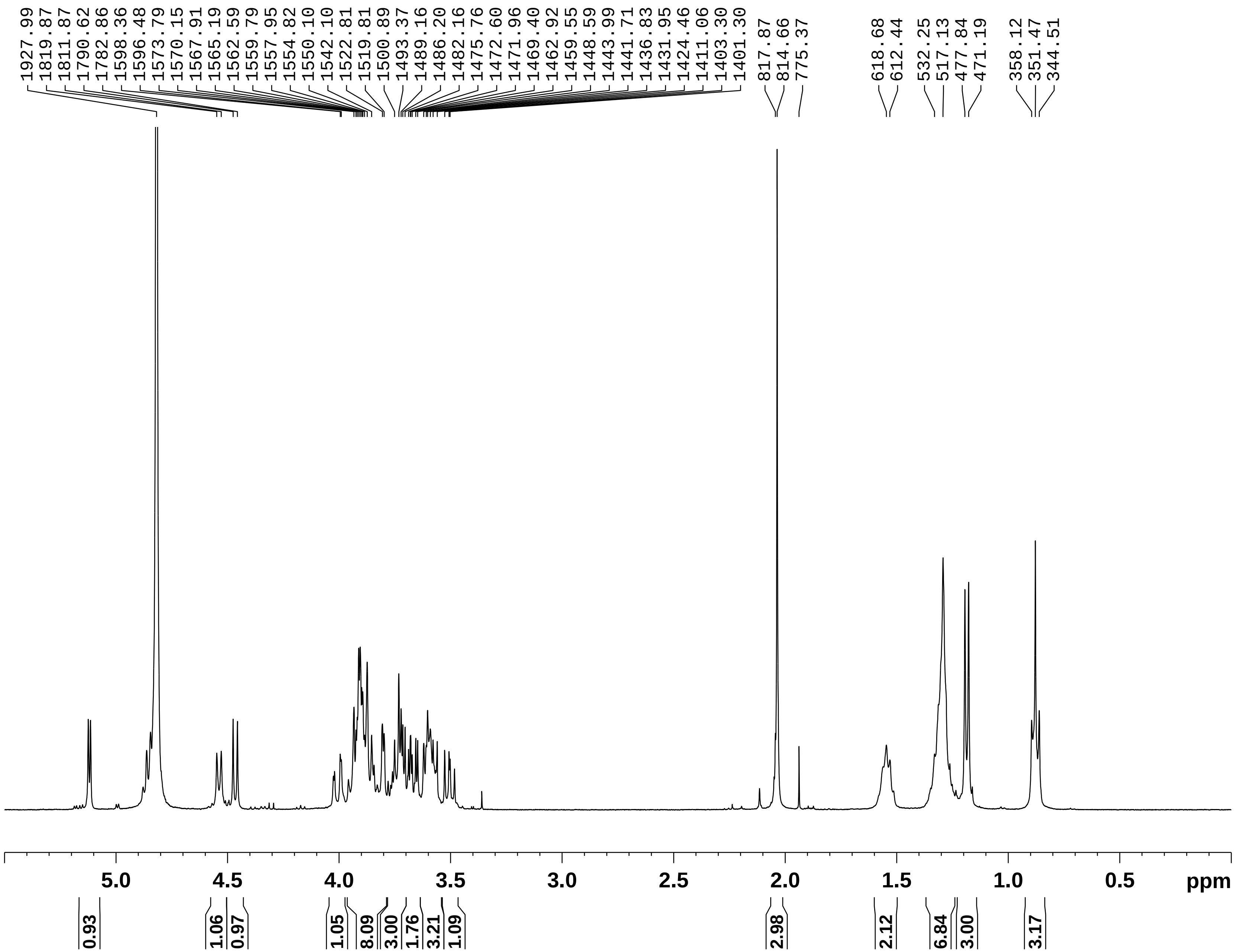

Compound **1**, 100 MHz, D_2O

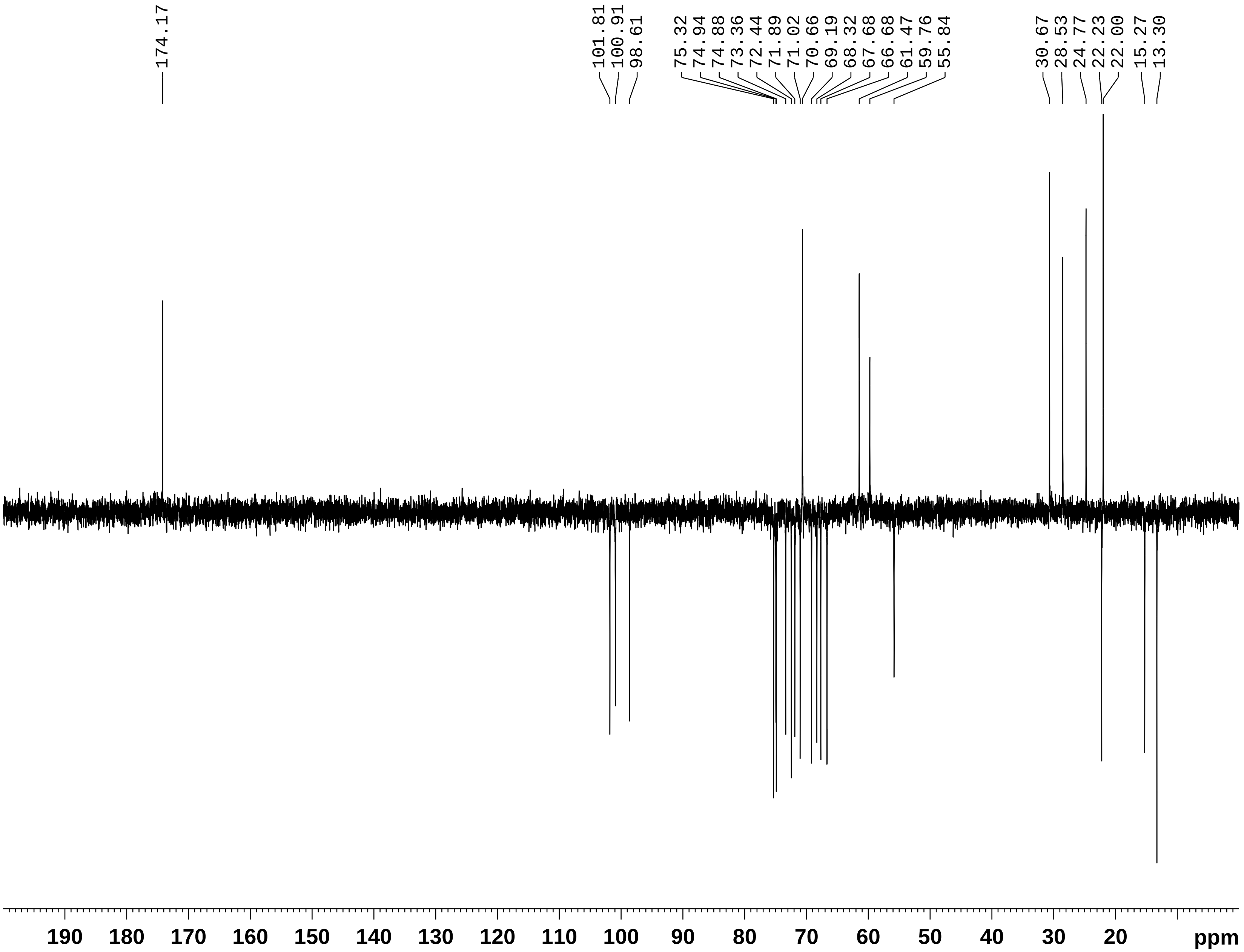

Compound **2**, 400 MHz, D_2O

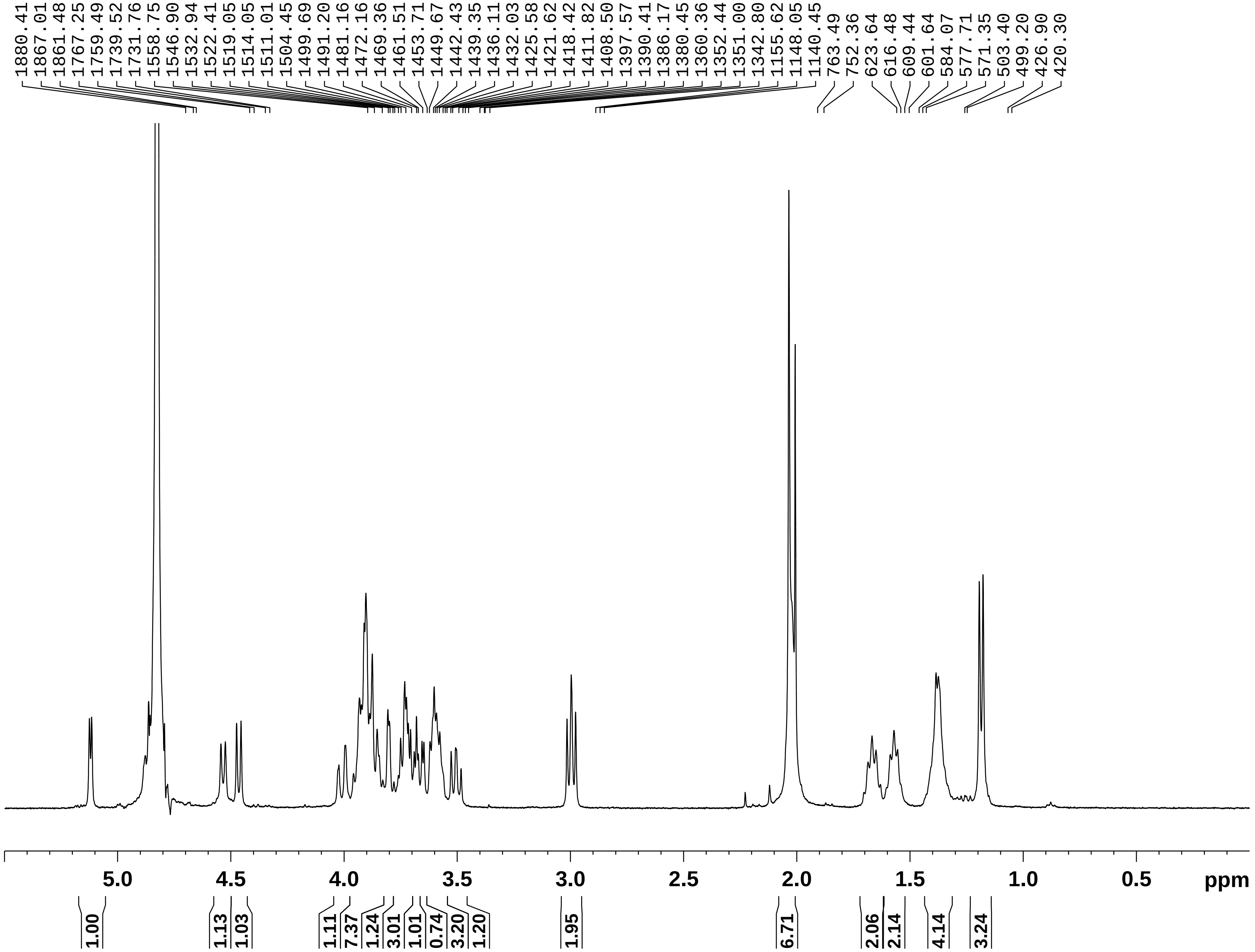

Compound **2**, 100 MHz, D_2O

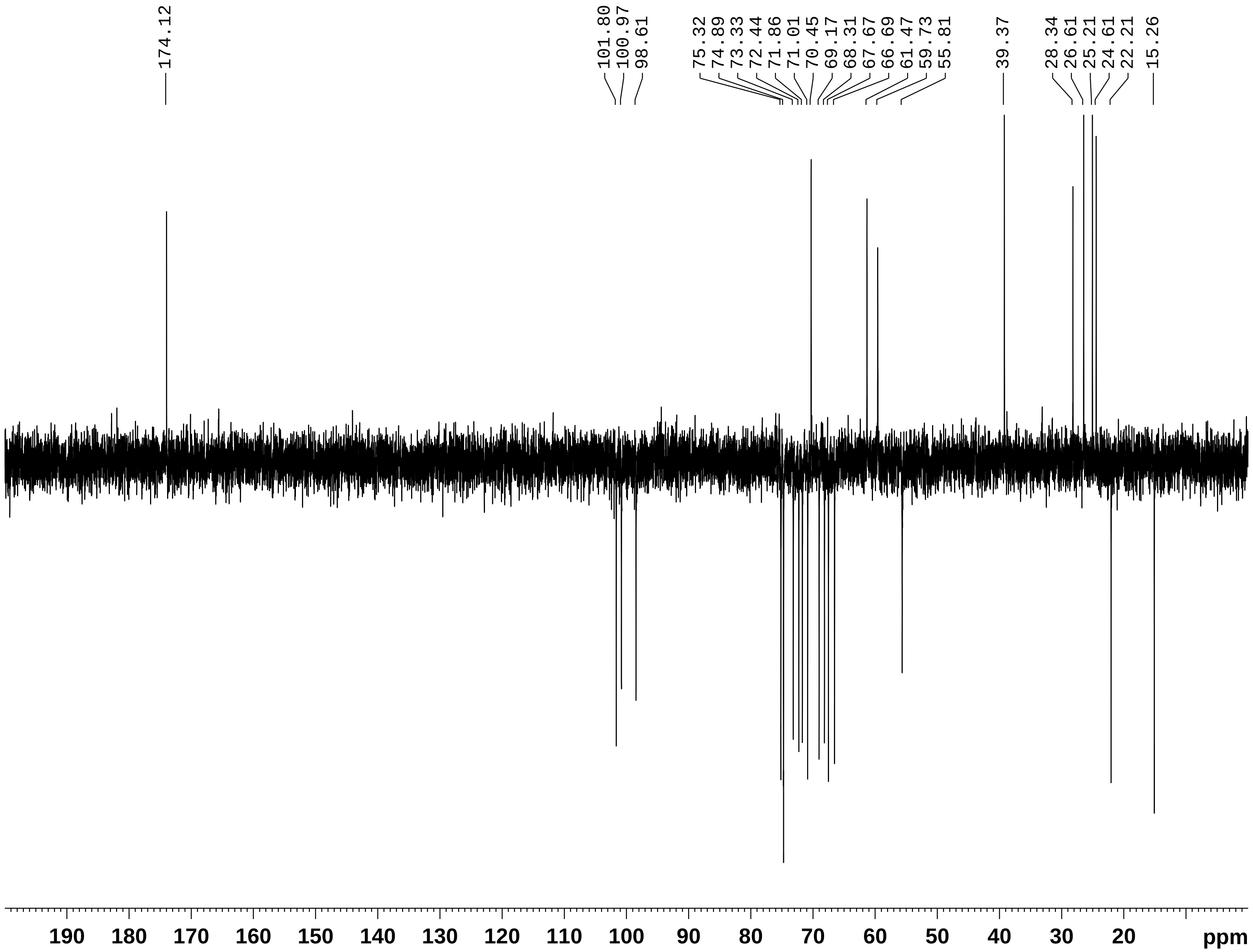

Compound **3**, 600 MHz, D_2O

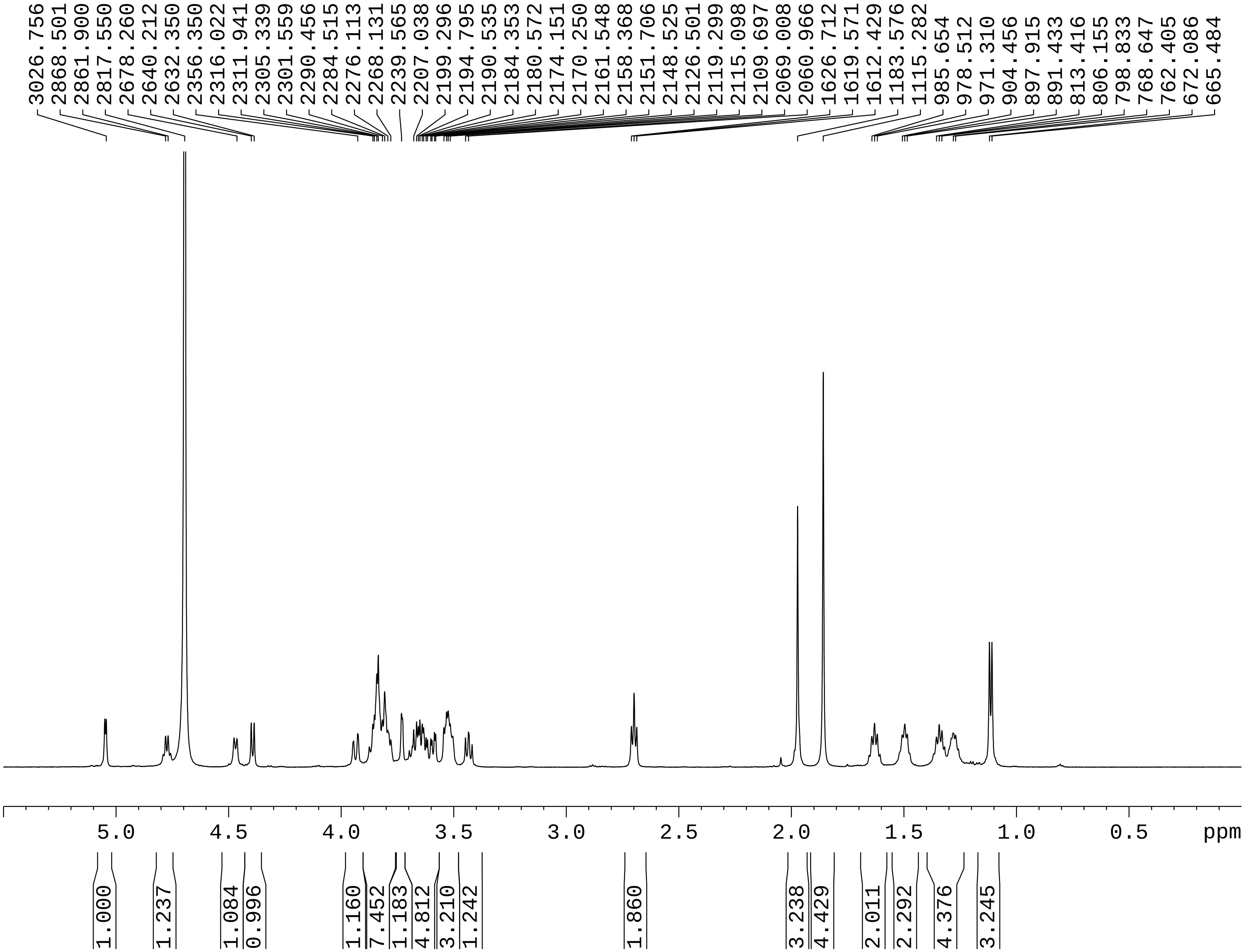

Compound **3**, 150 MHz, D_2O

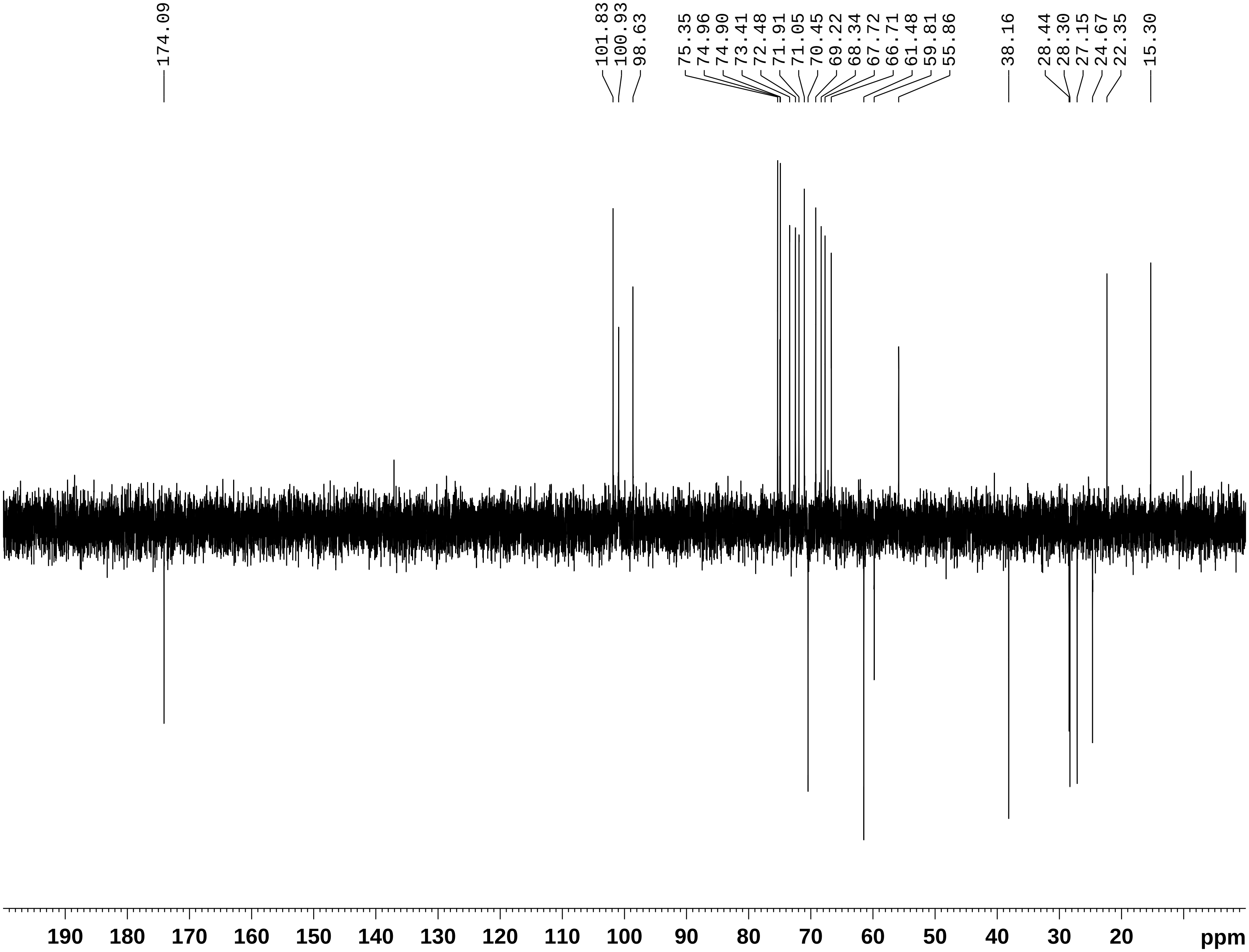

Compound **4**, 300 MHz, $CDCl_3$

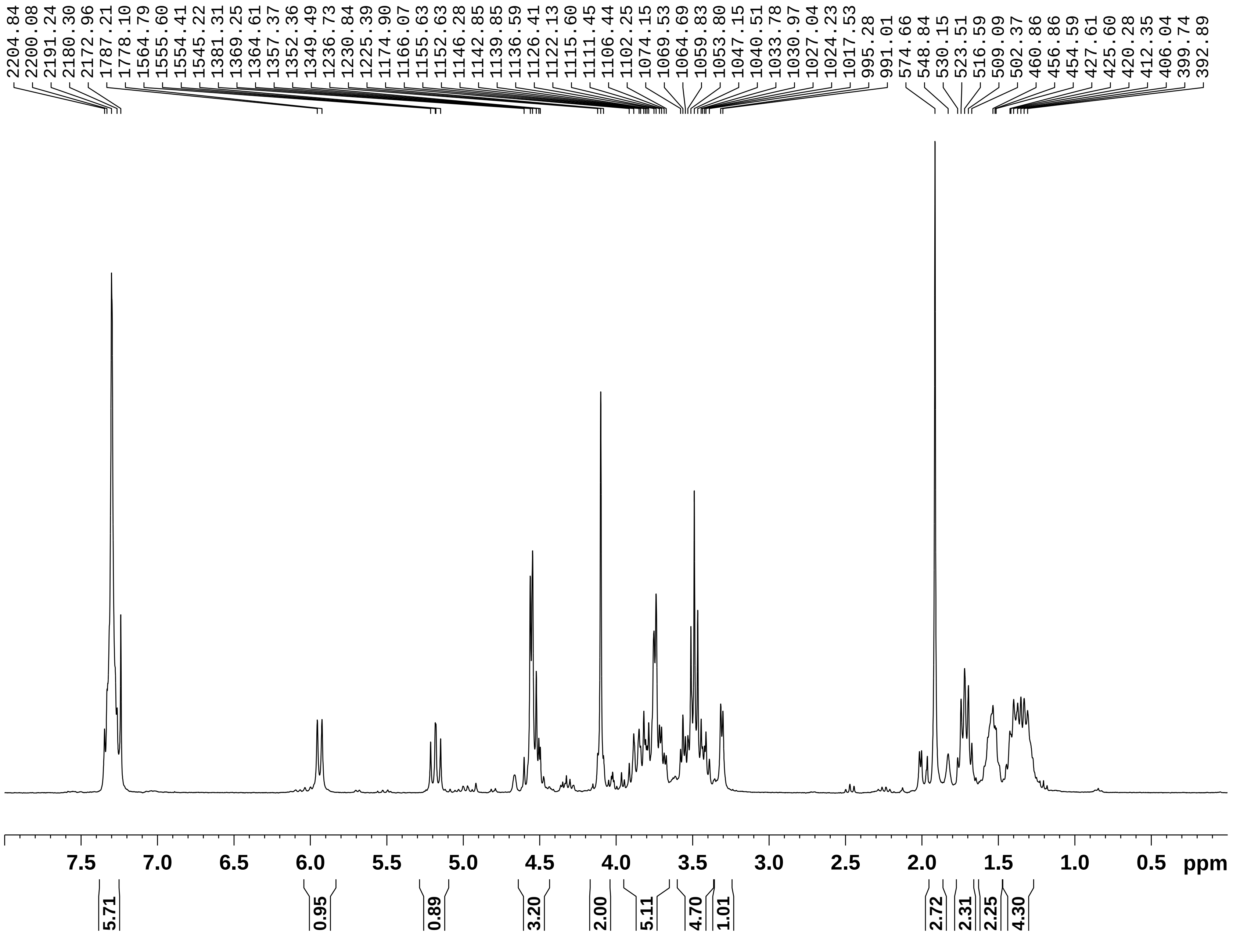

Compound **4**, 75 MHz, $CDCl_3$

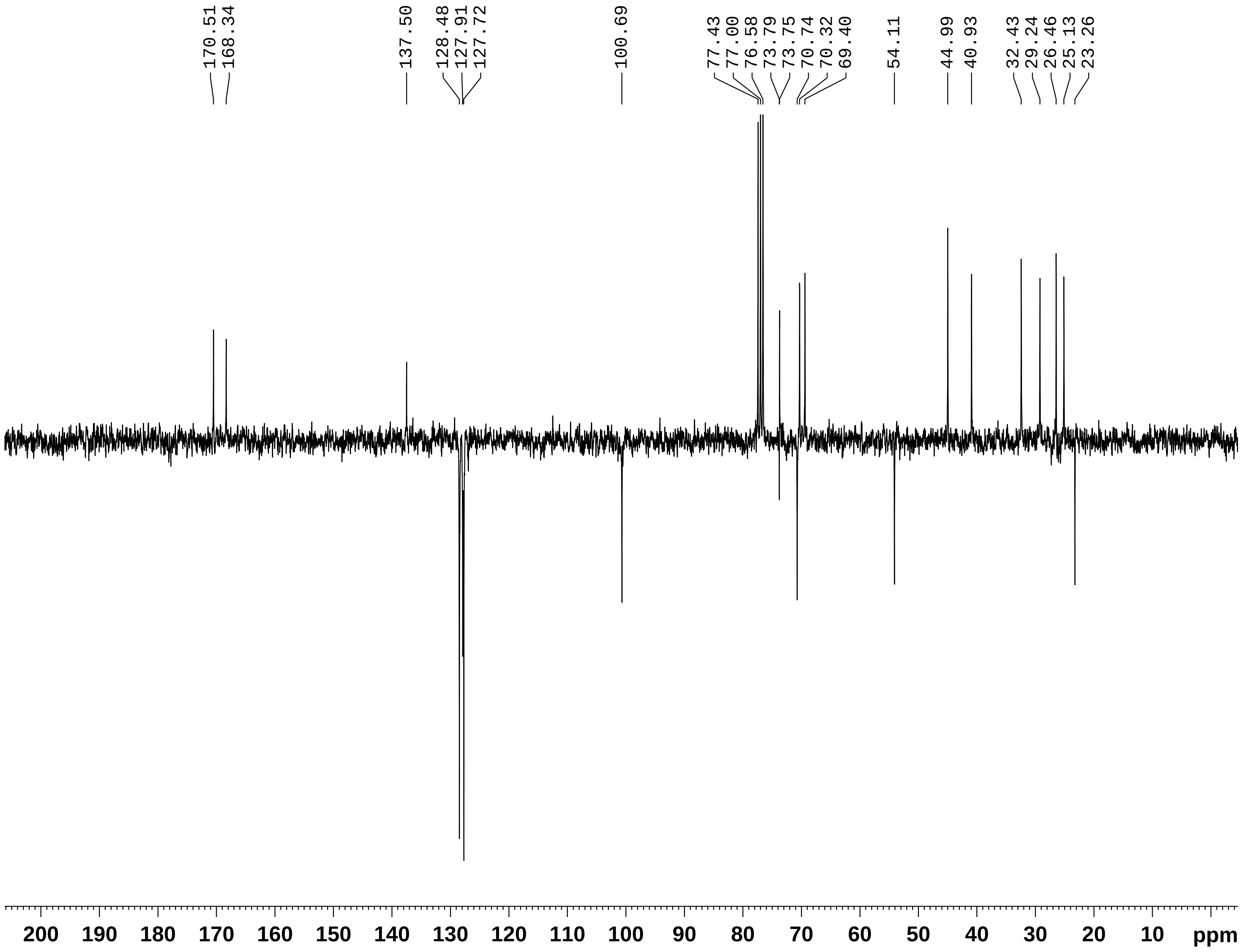

Compound **5**, 400 MHz, $CDCl_3$

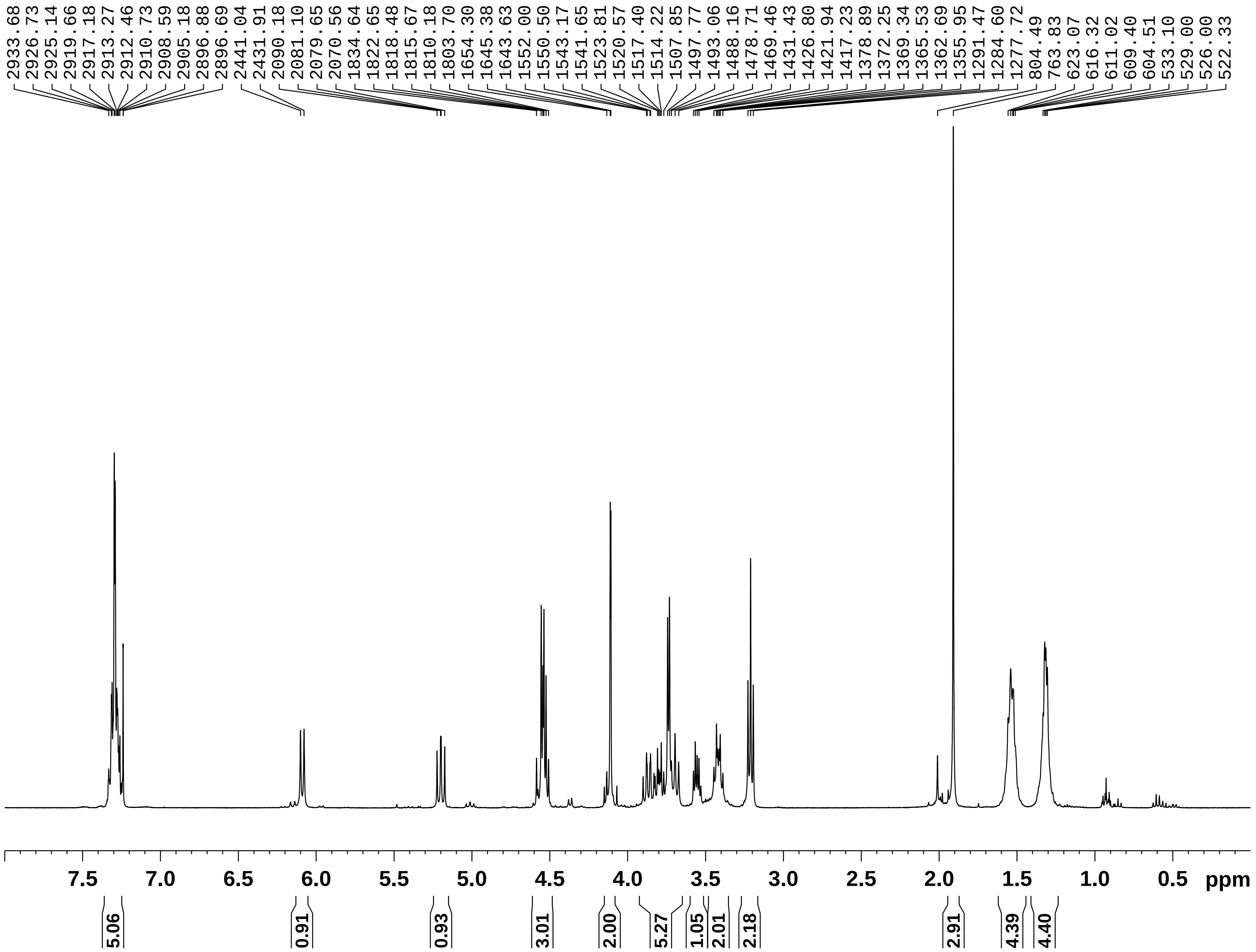

Compound **5**, 100 MHz, $CDCl_3$

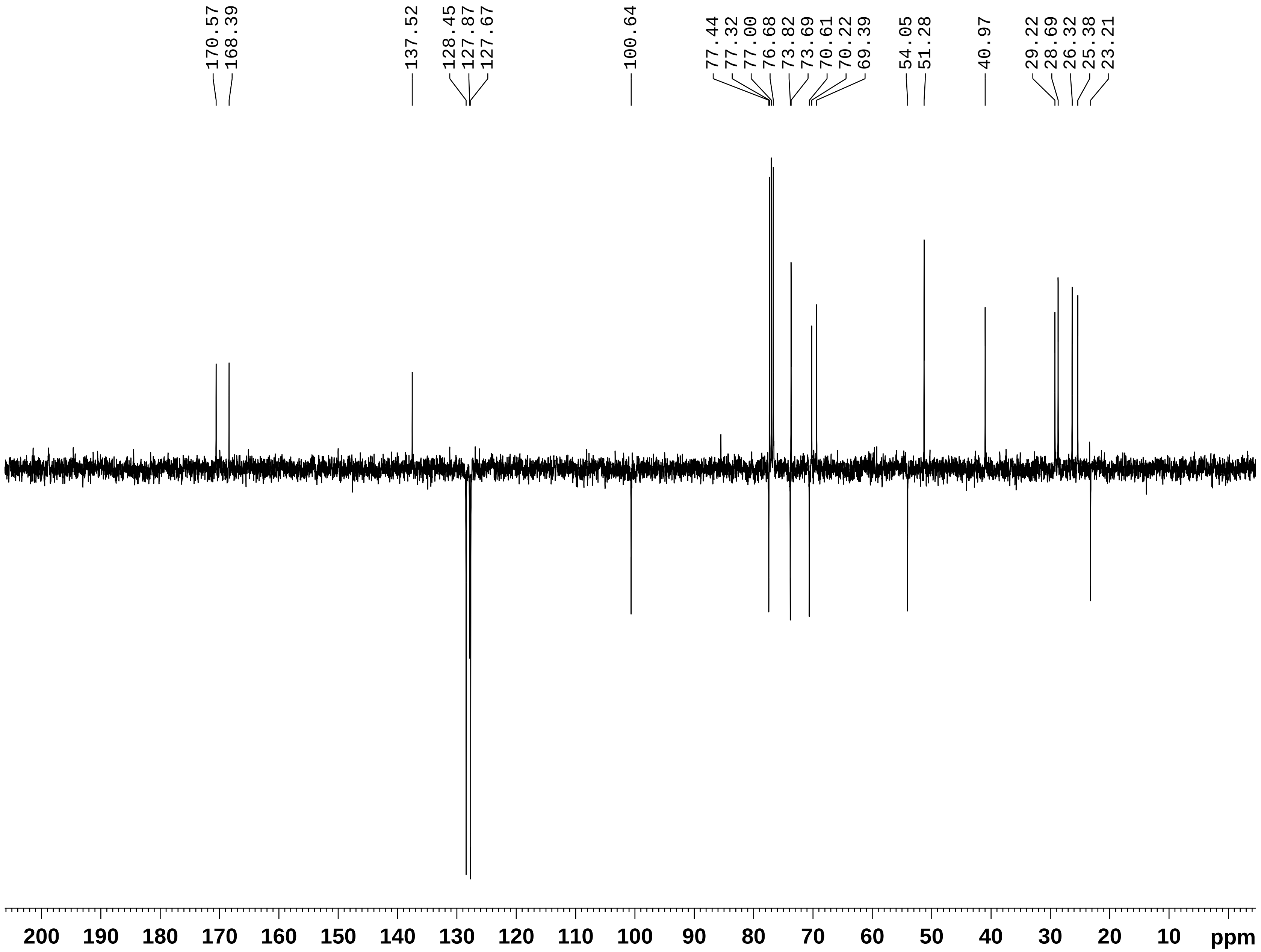

Compound **6**, 400 MHz, $CDCl_3$

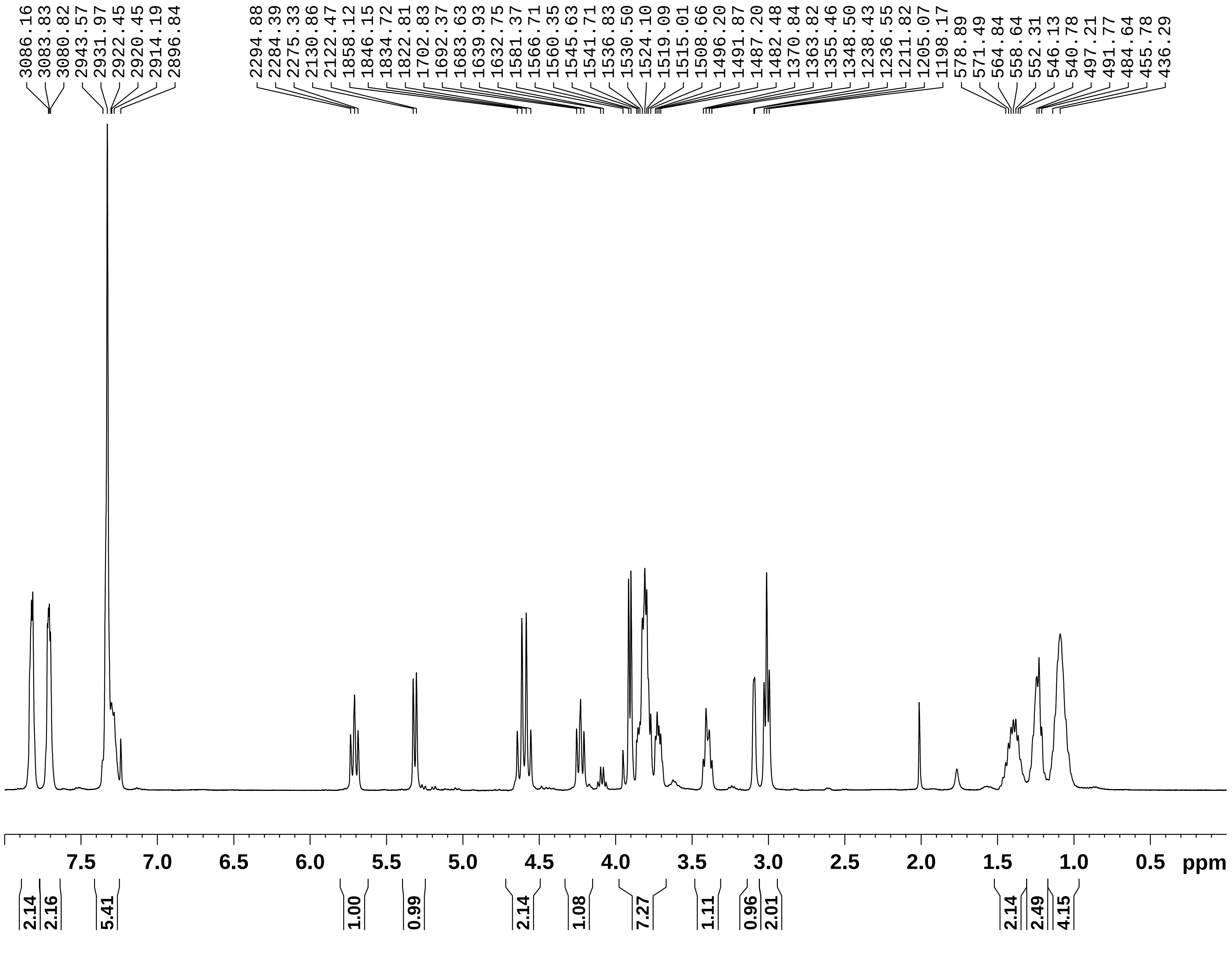

Compound **6**, 100 MHz, $CDCl_3$

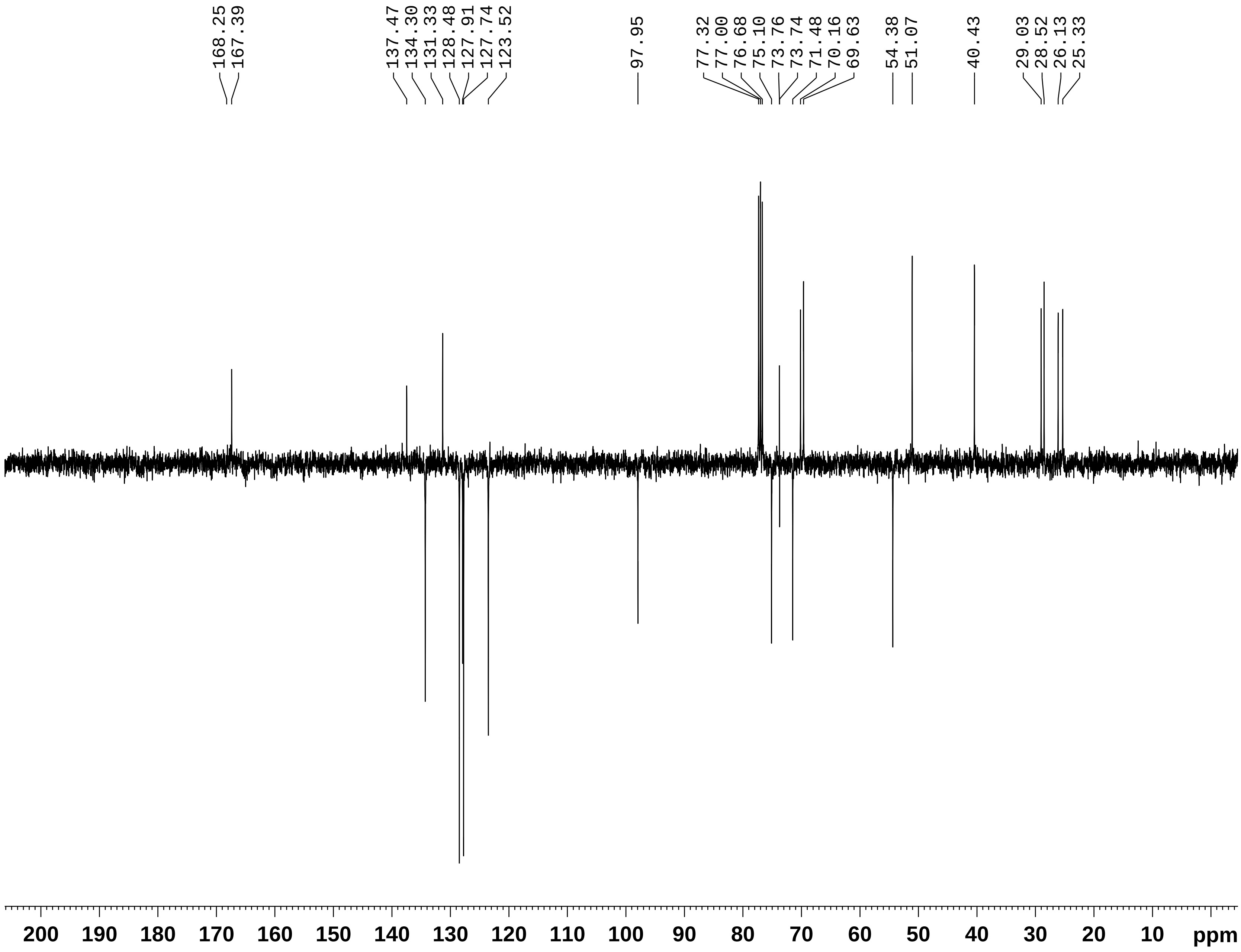

Compound **8**, 400 MHz, $CDCl_3$

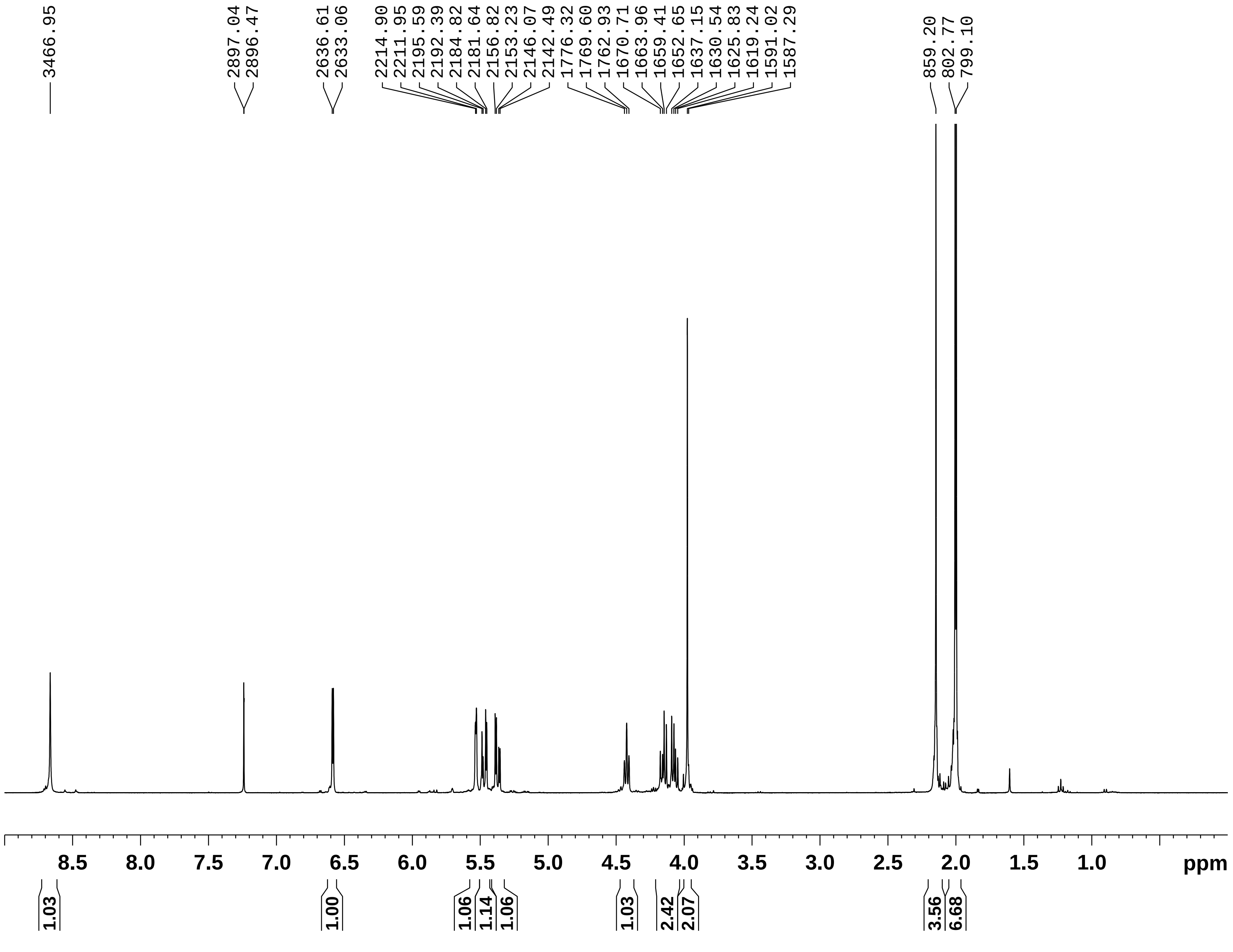

Compound **8**, 100 MHz, $CDCl_3$

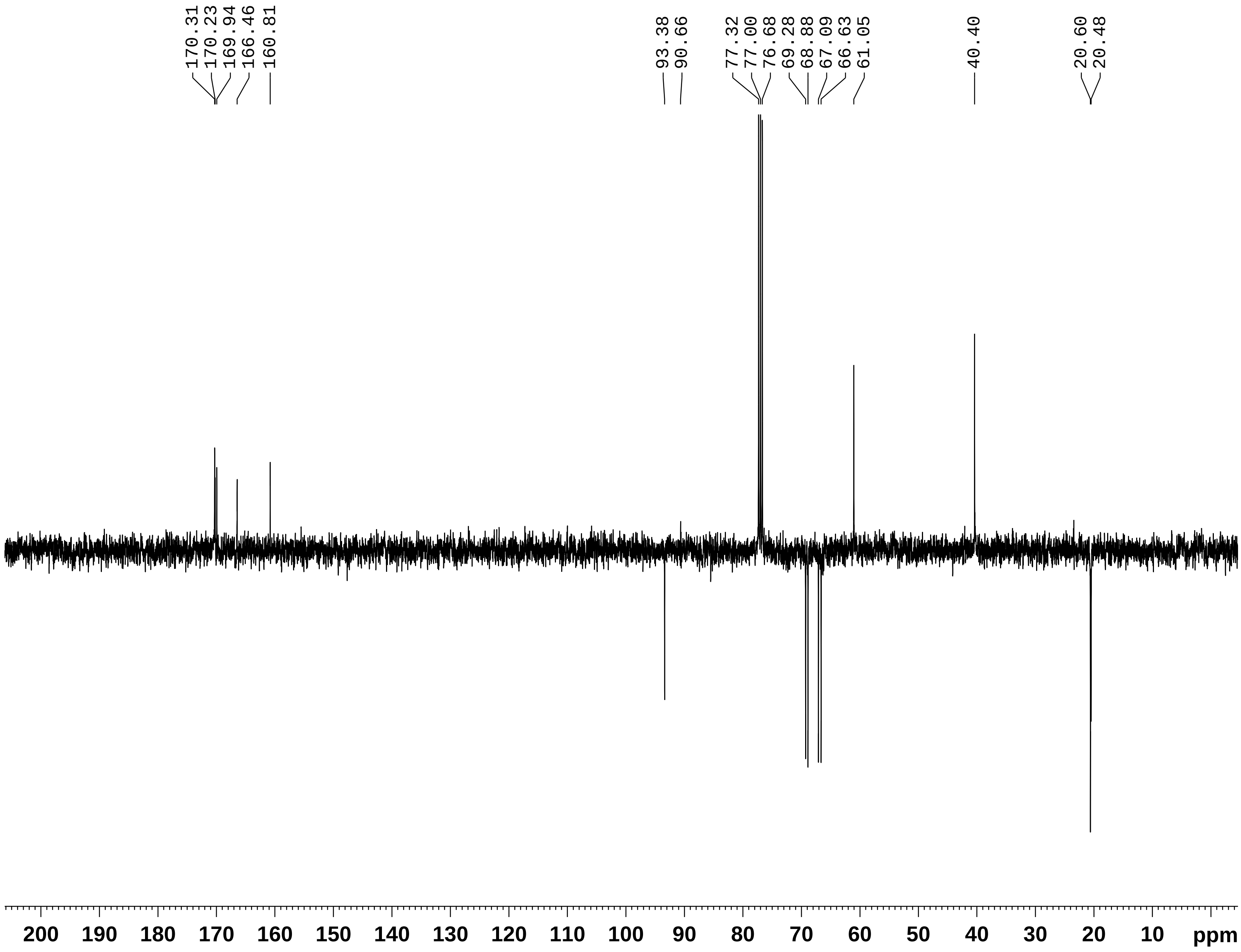

Compound **11**, 400 MHz, $CDCl_3$

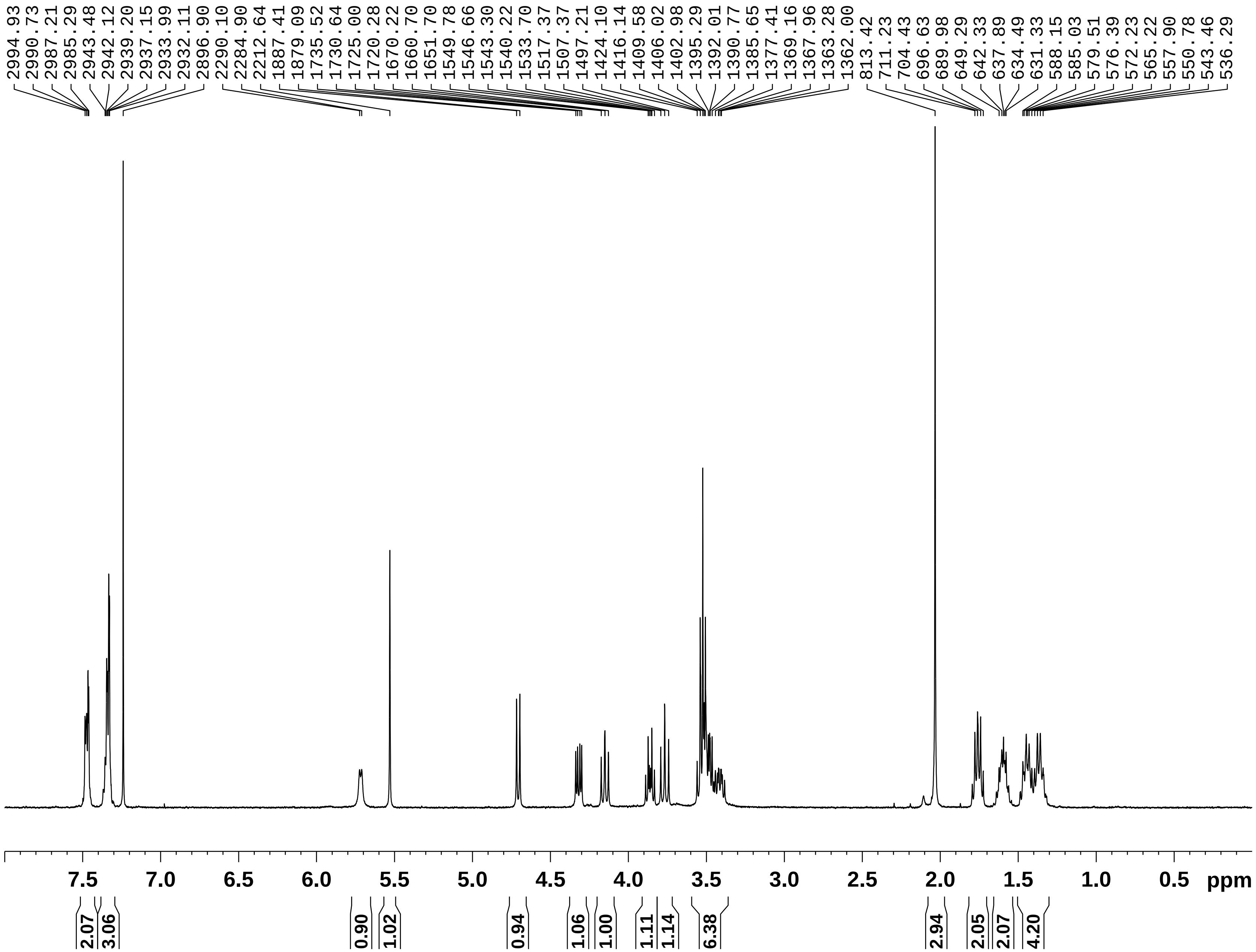

Compound **11**, 100 MHz, $CDCl_3$

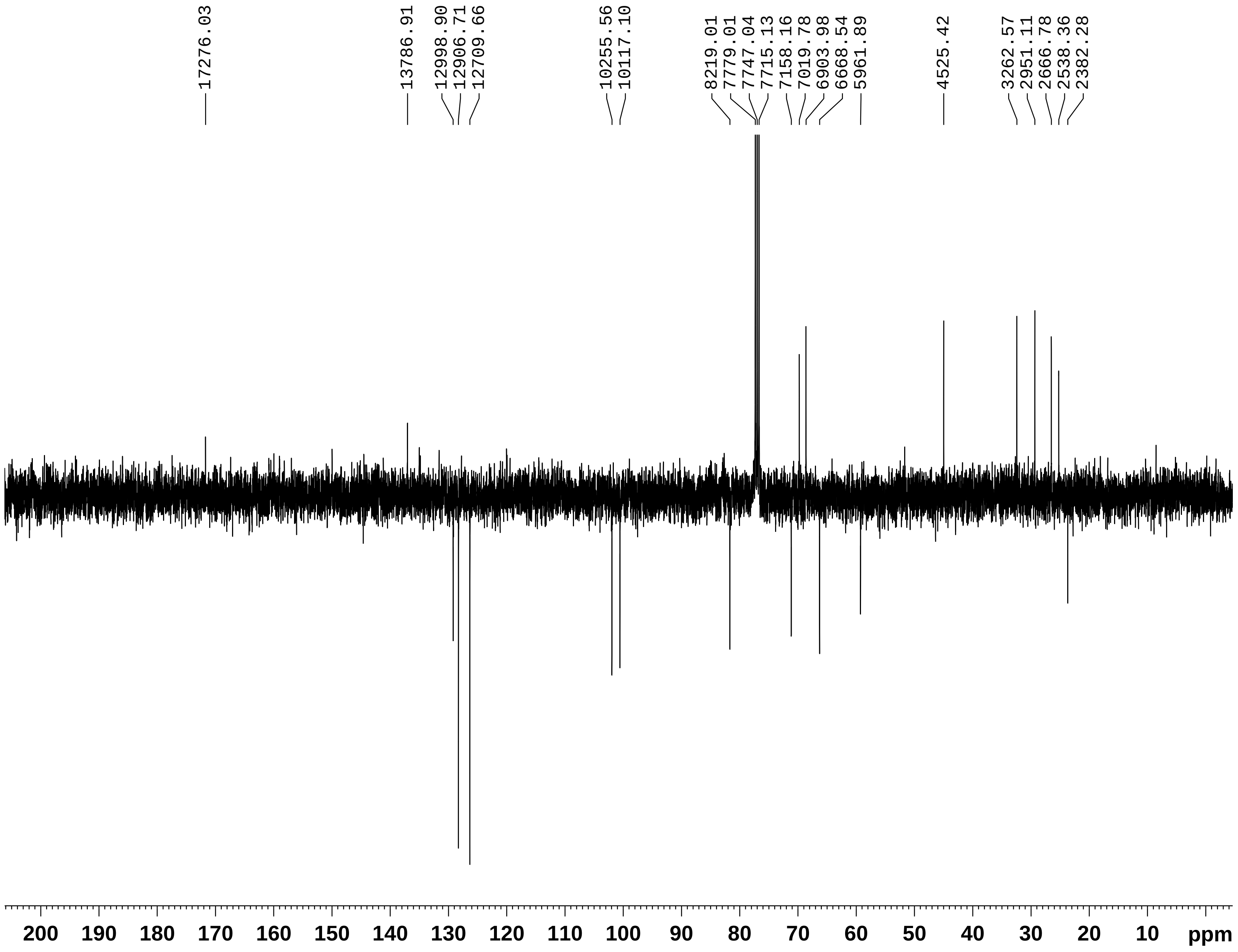

Compound **12**, 400 MHz, $CDCl_3$

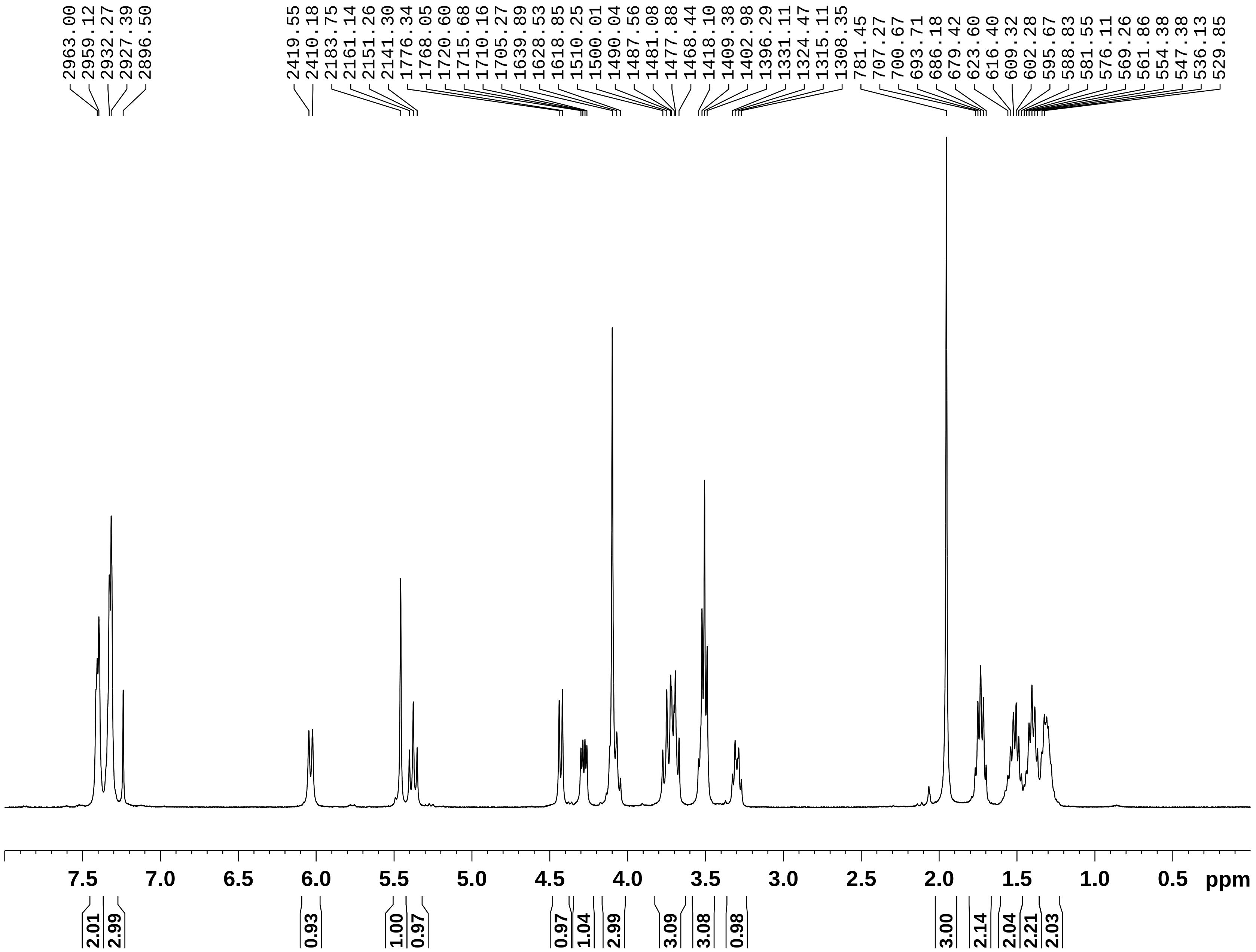

Compound **12**, 100 MHz, $CDCl_3$

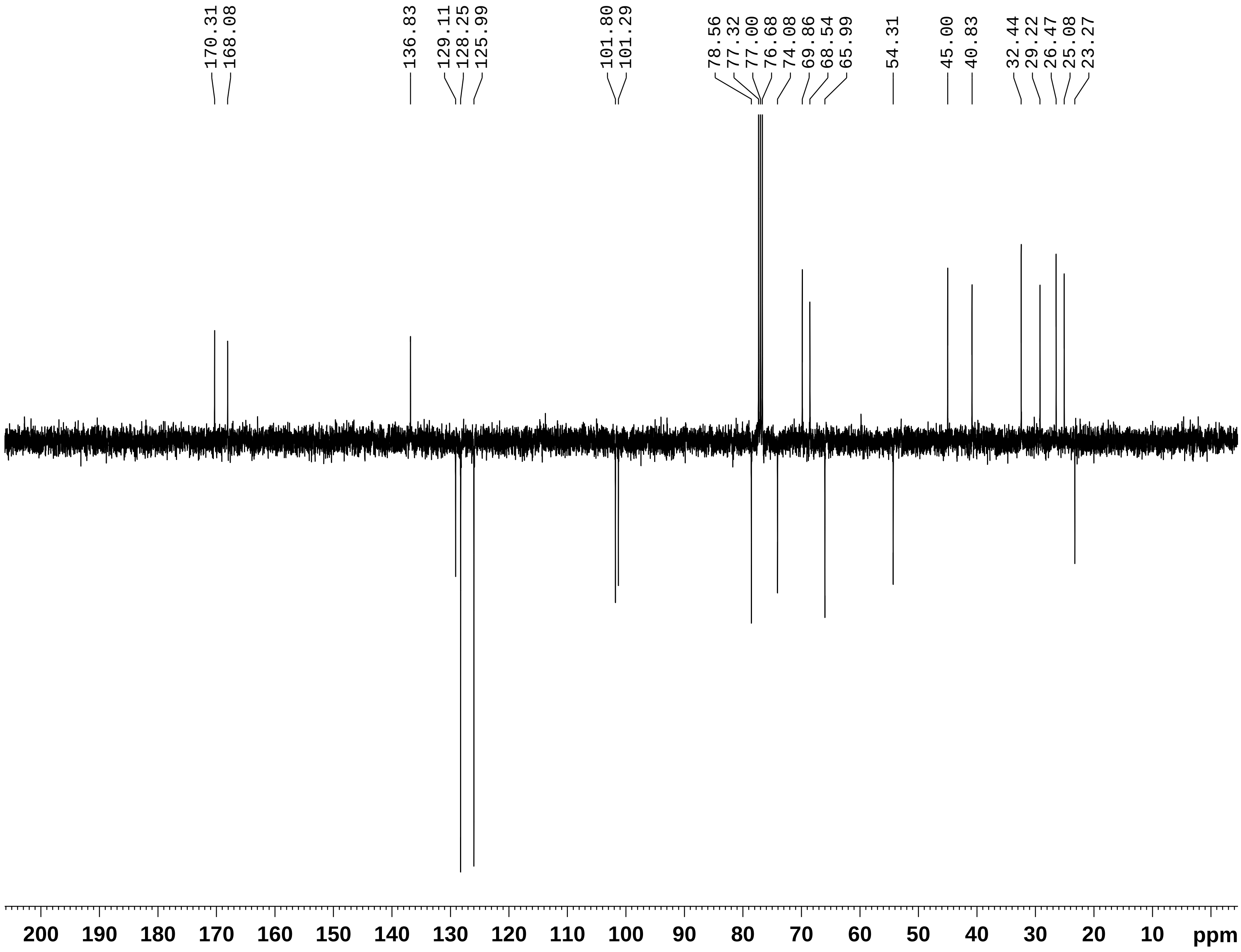

Compound **14**, 300 MHz, $CDCl_3$

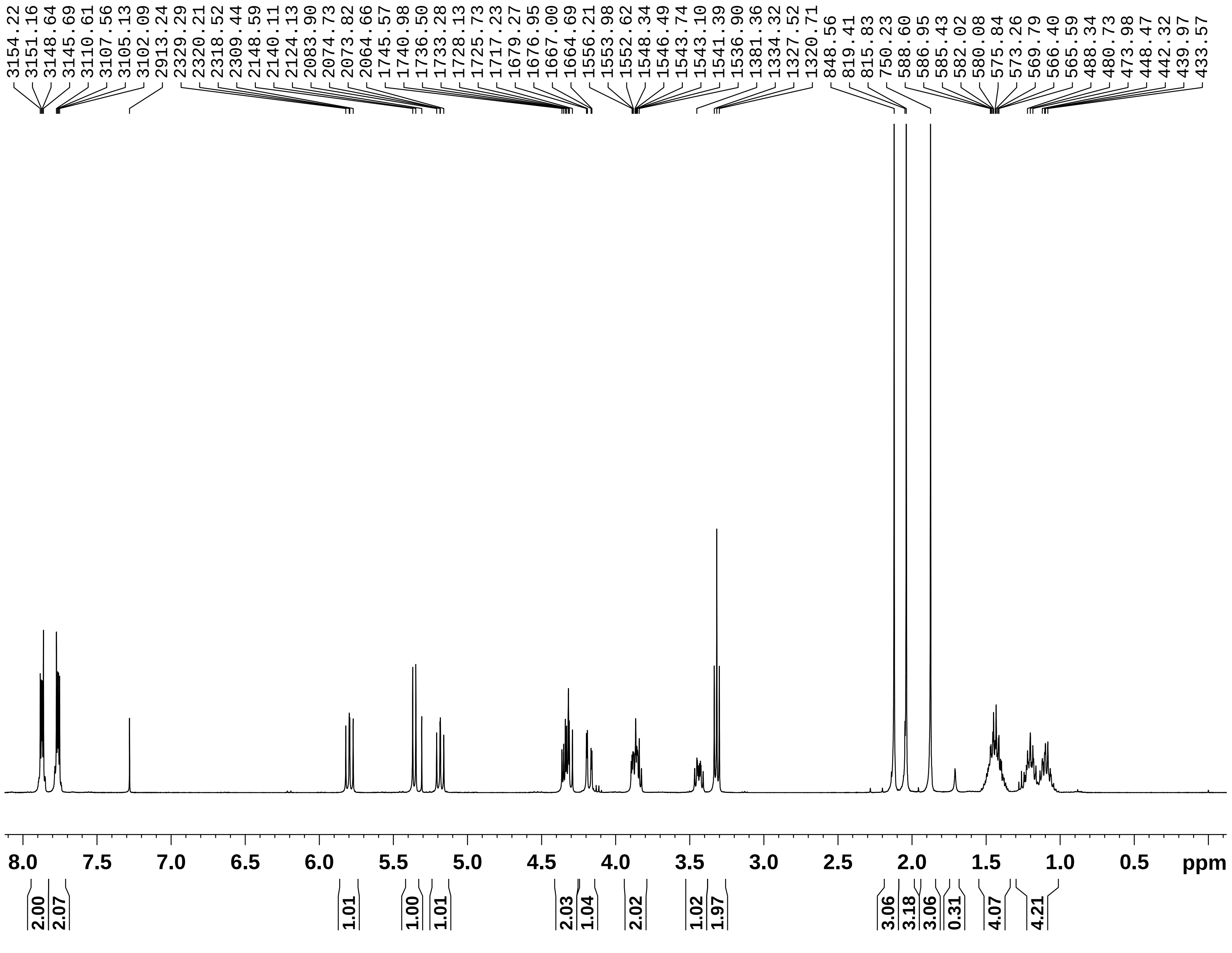

Compound **15**, 400 MHz, $CDCl_3$

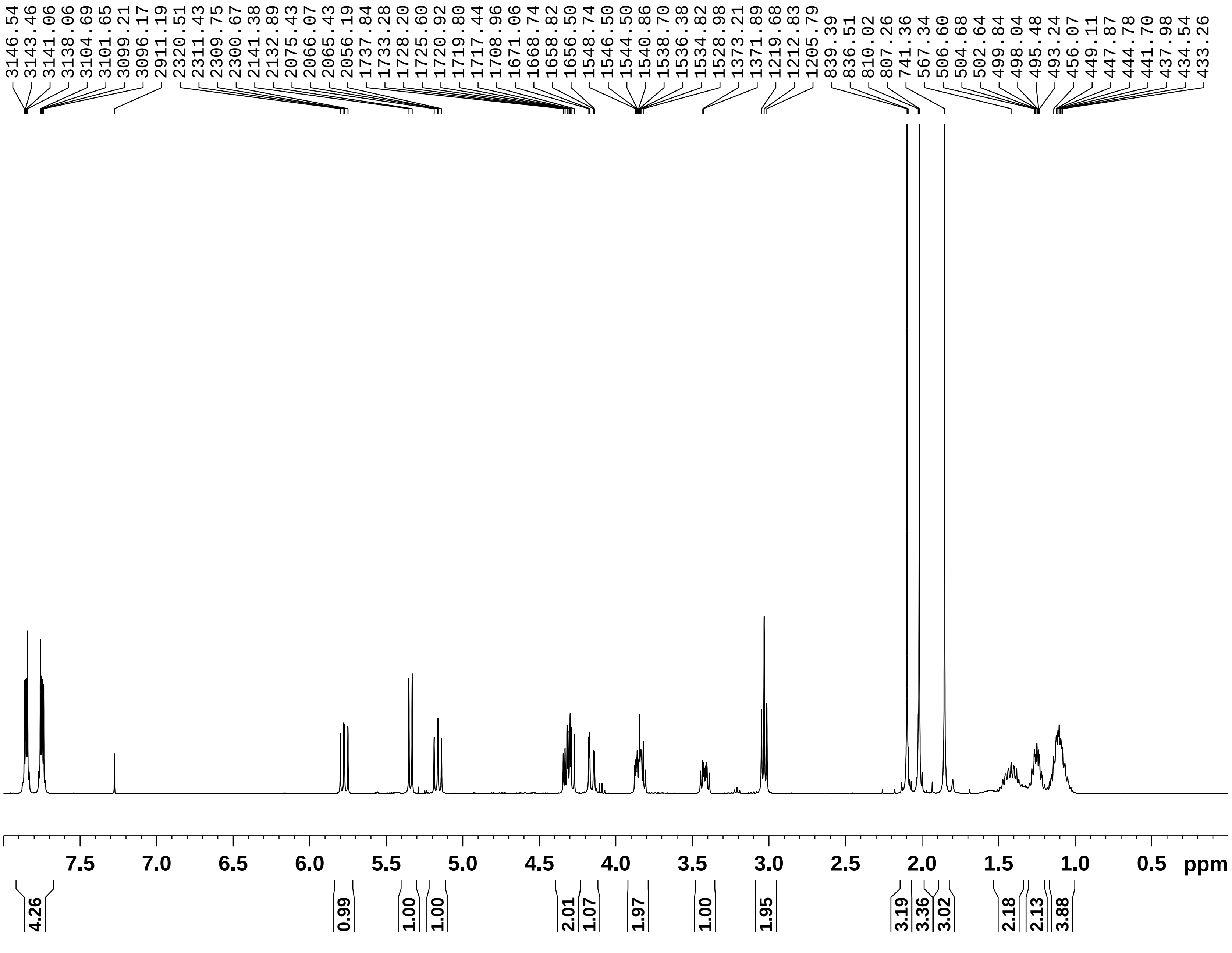

Compound **16**, 400 MHz, $CDCl_3$

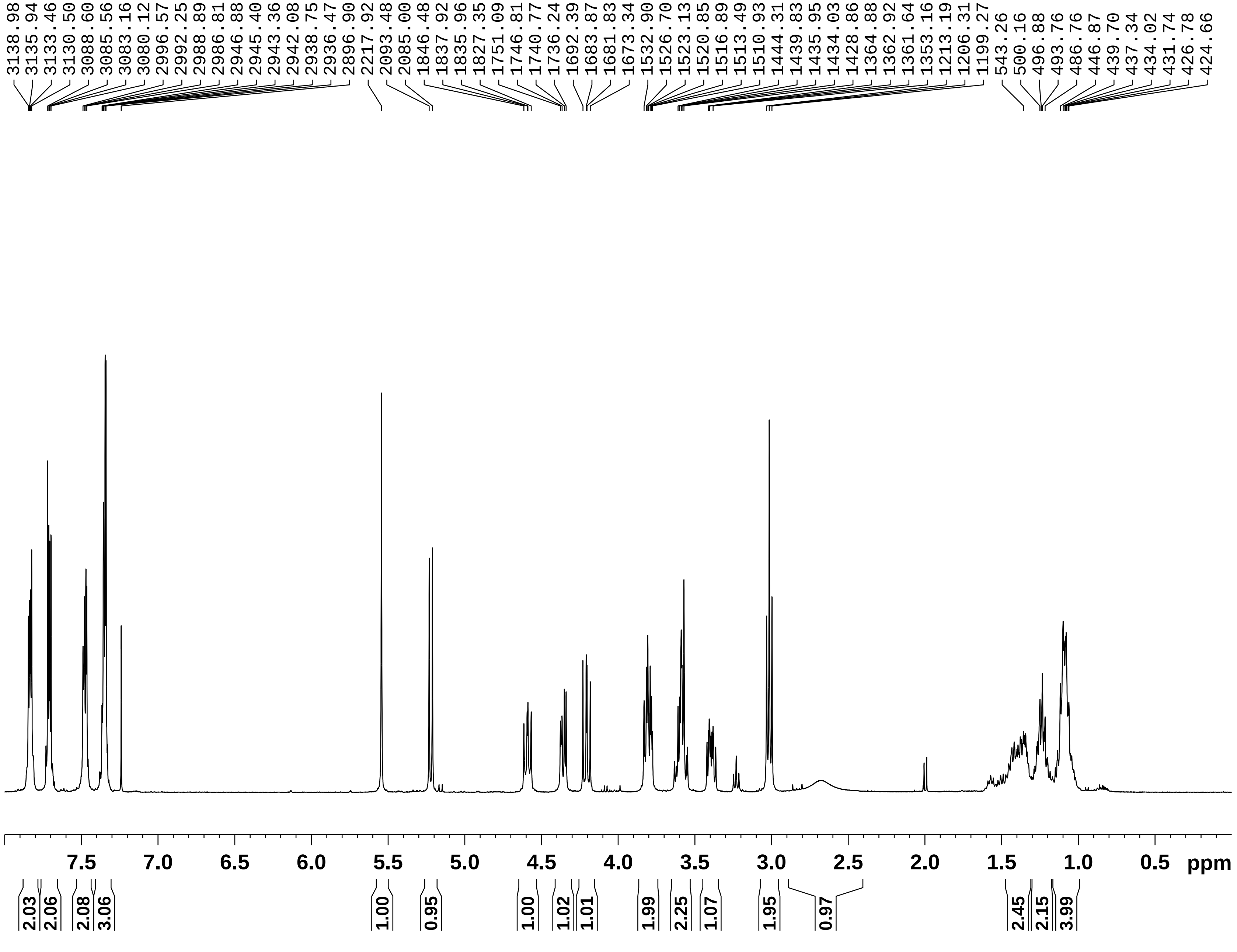

Compound **16**, 100 MHz, $CDCl_3$

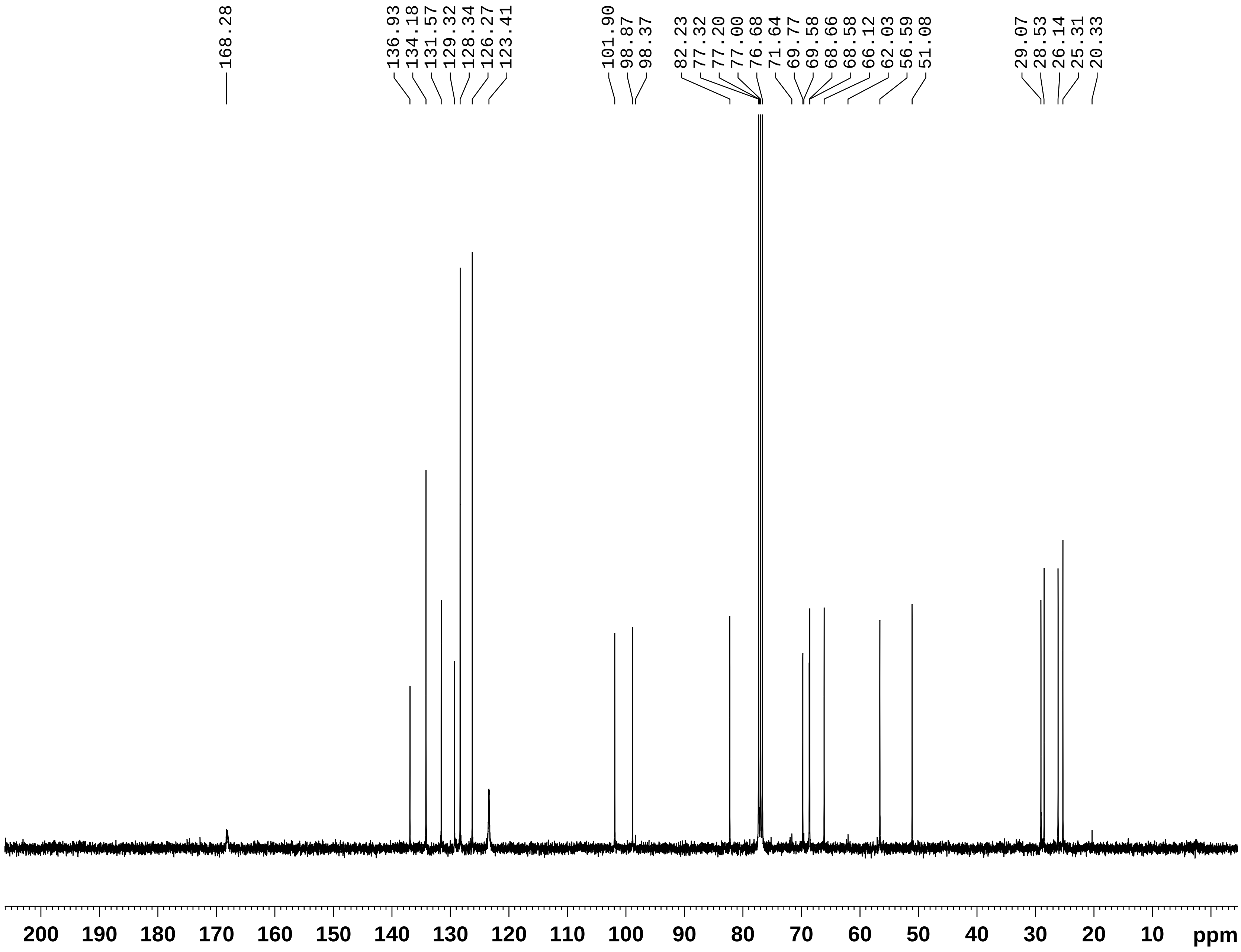

Compound **17**, 400 MHz, $CDCl_3$

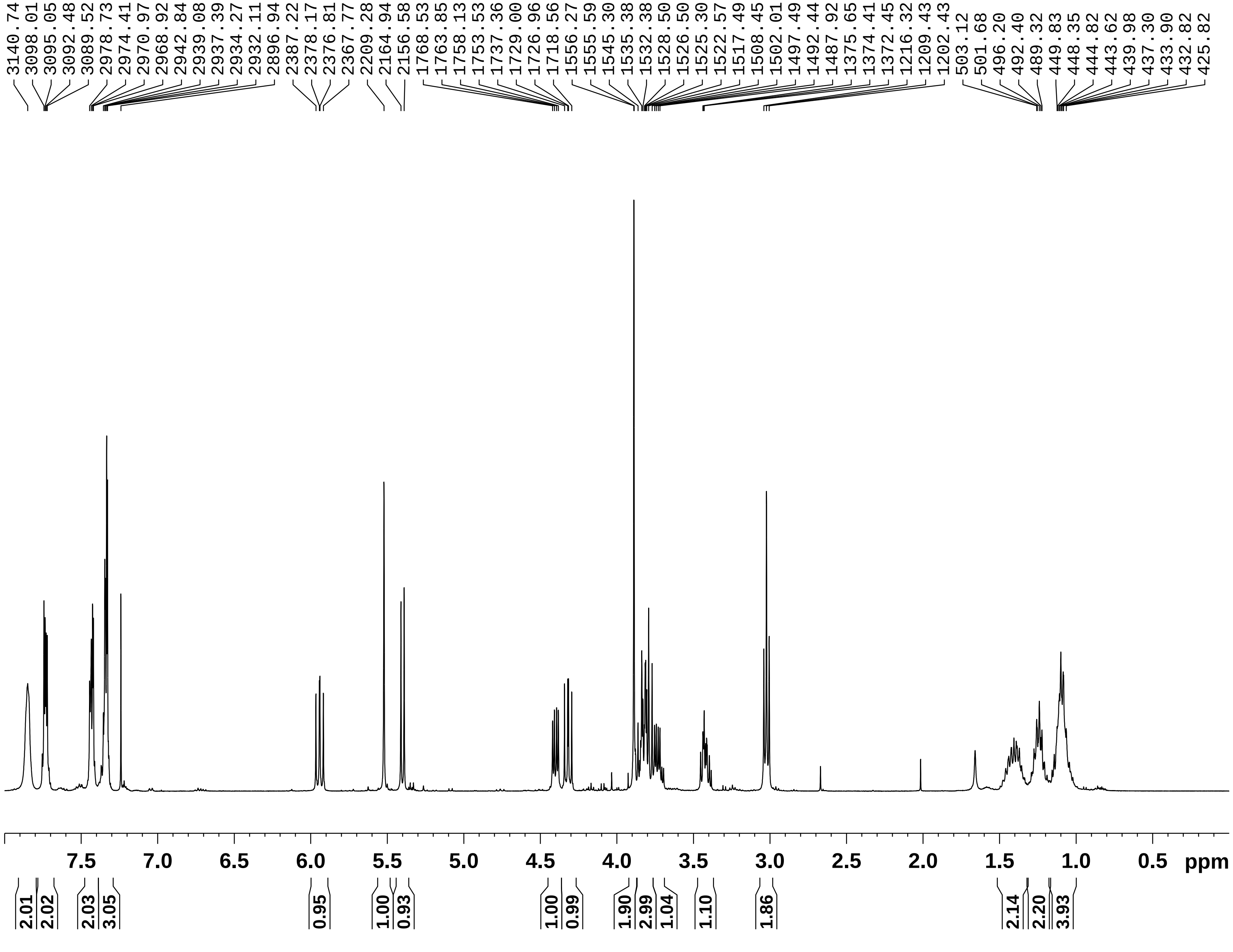

Compound **17**, 100 MHz, $CDCl_3$

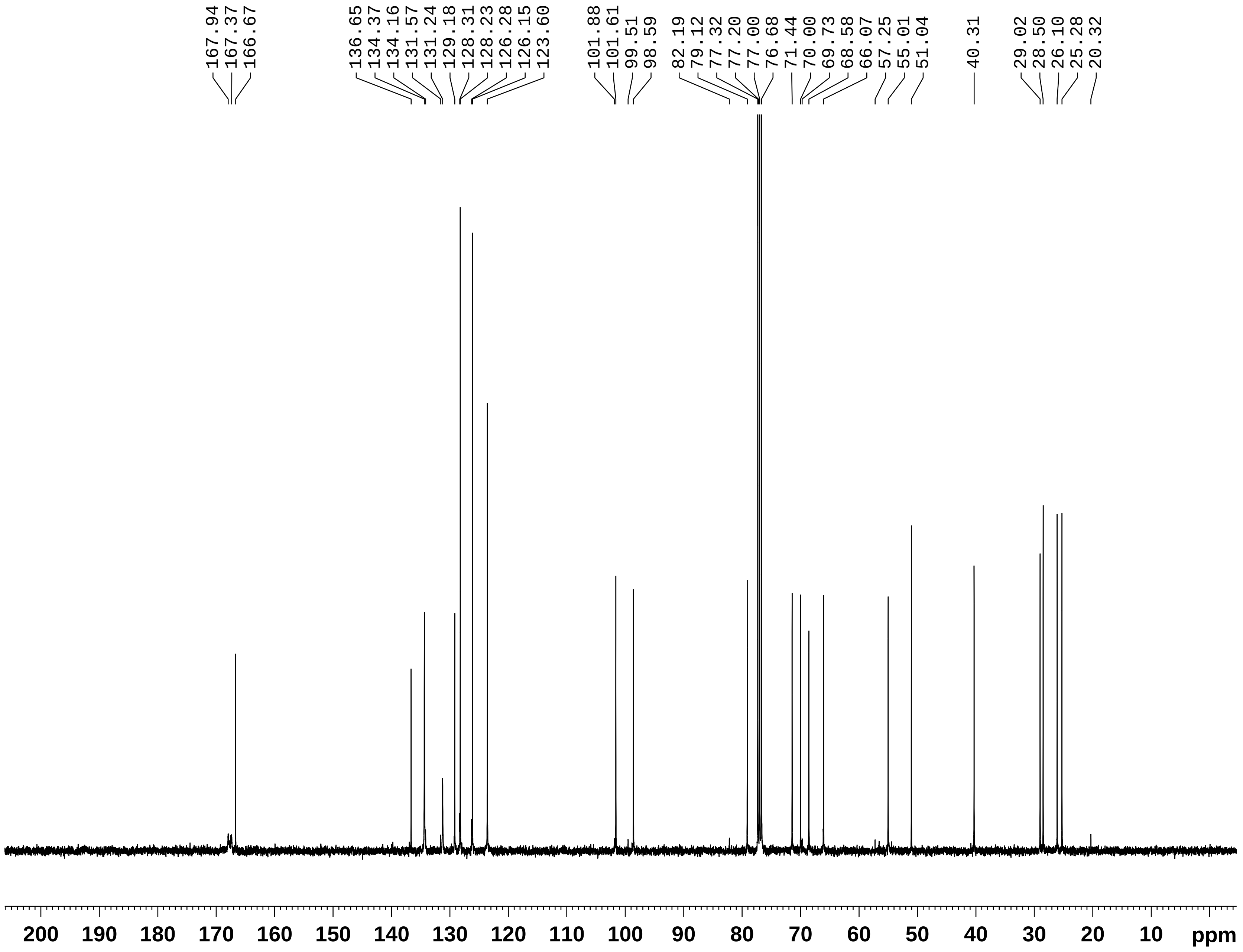

Compound **18**, 400 MHz, $CDCl_3$

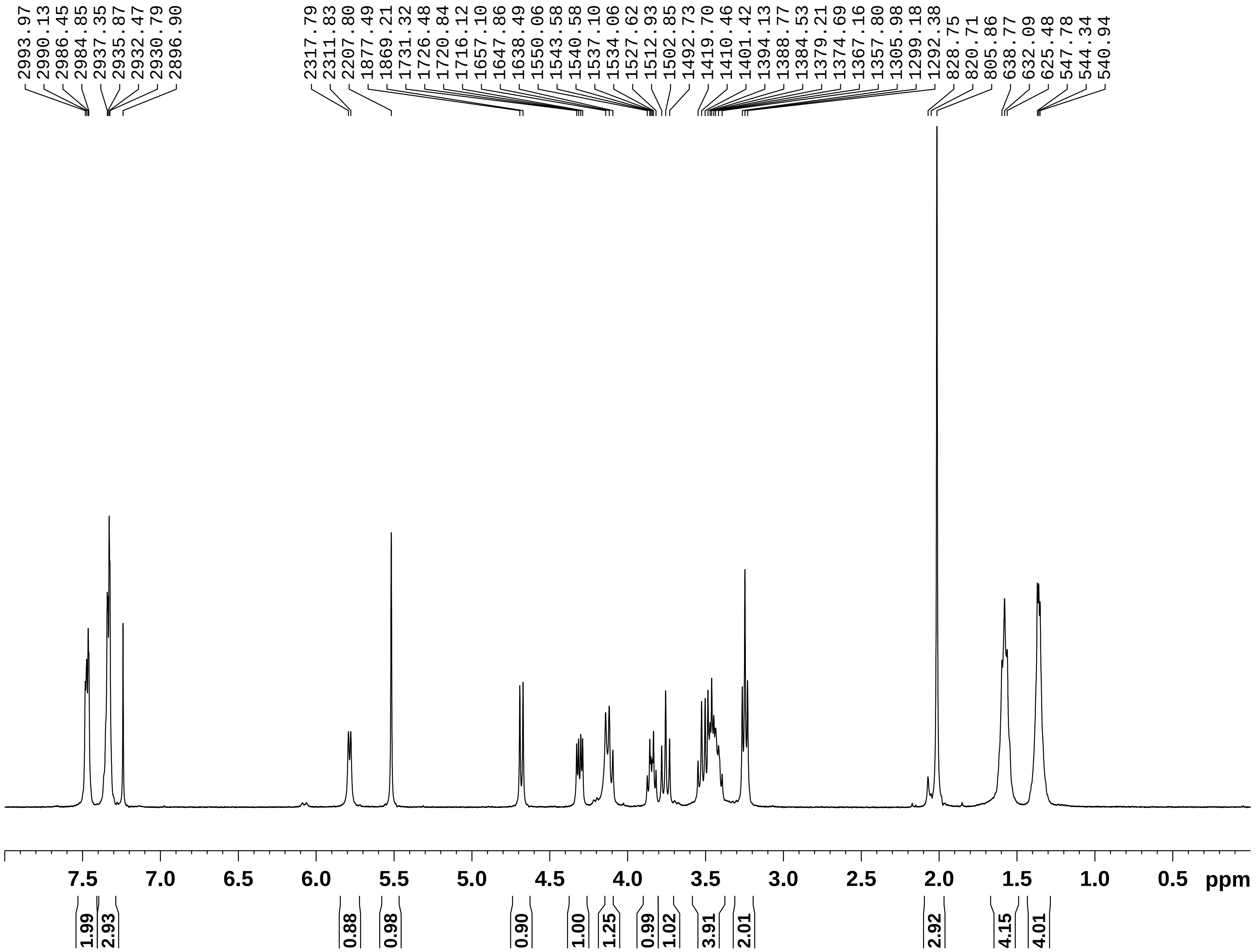

Compound **18**, 100 MHz, $CDCl_3$

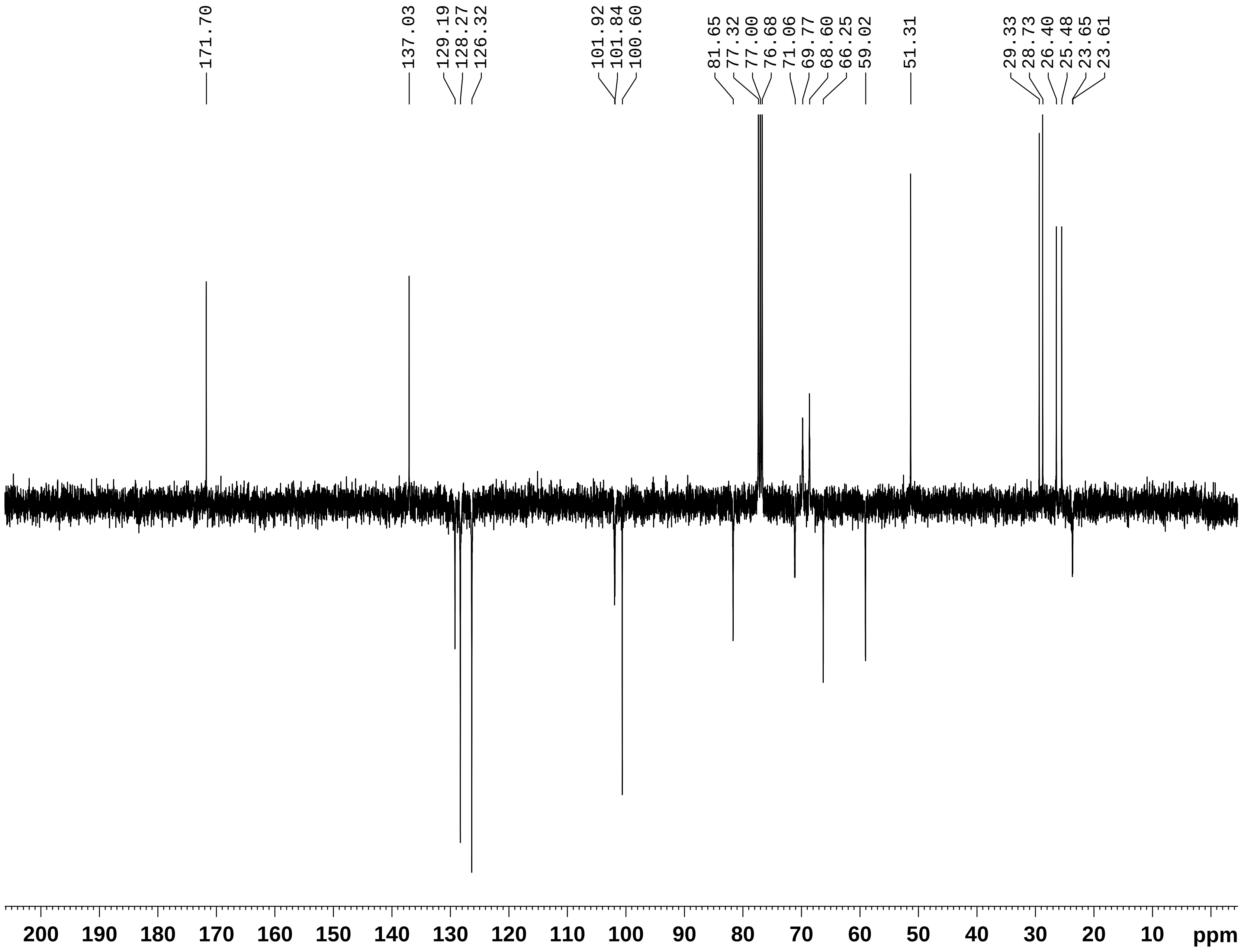

Compound **19**, 400 MHz, $CDCl_3$

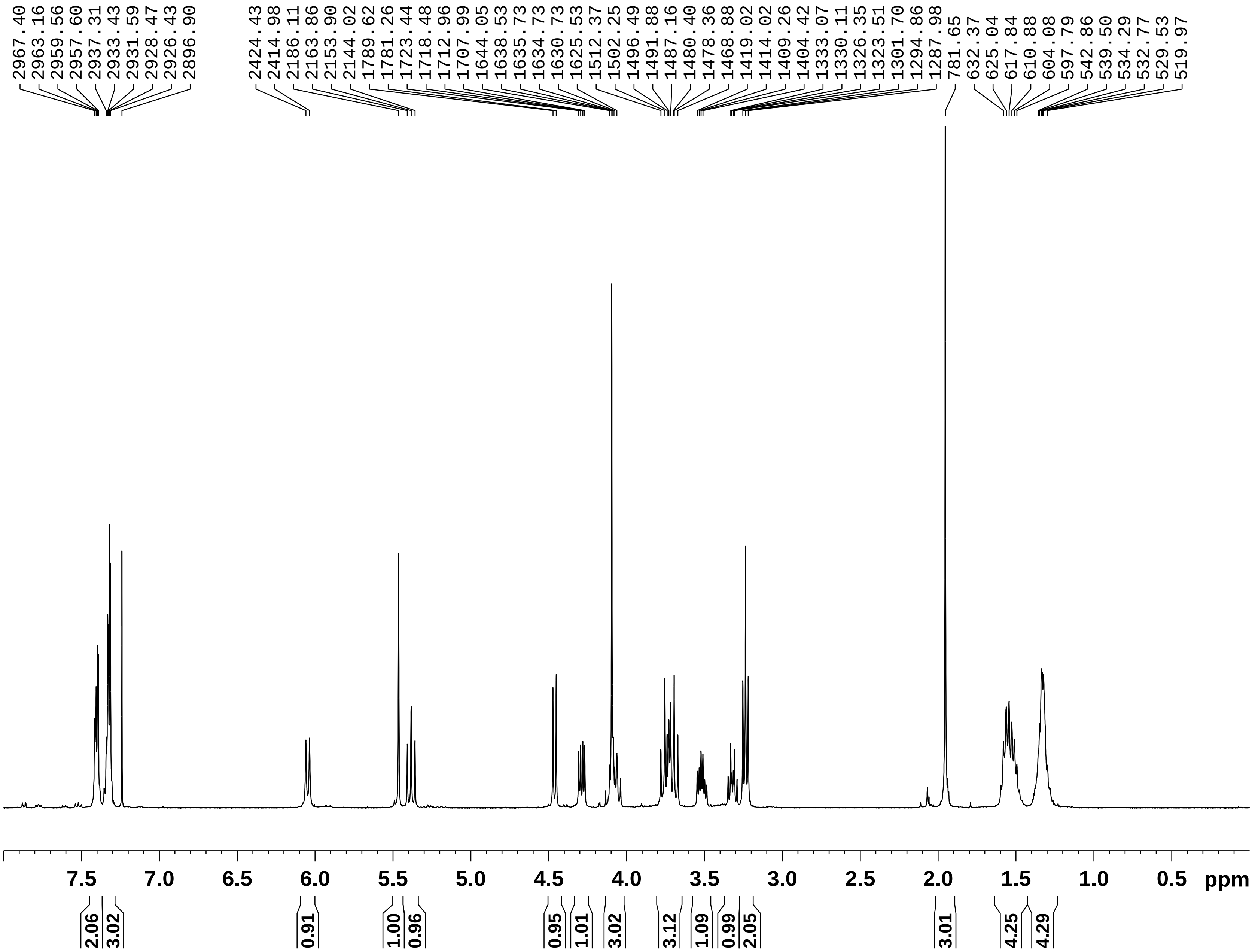

Compound **19**, 100 MHz, $CDCl_3$

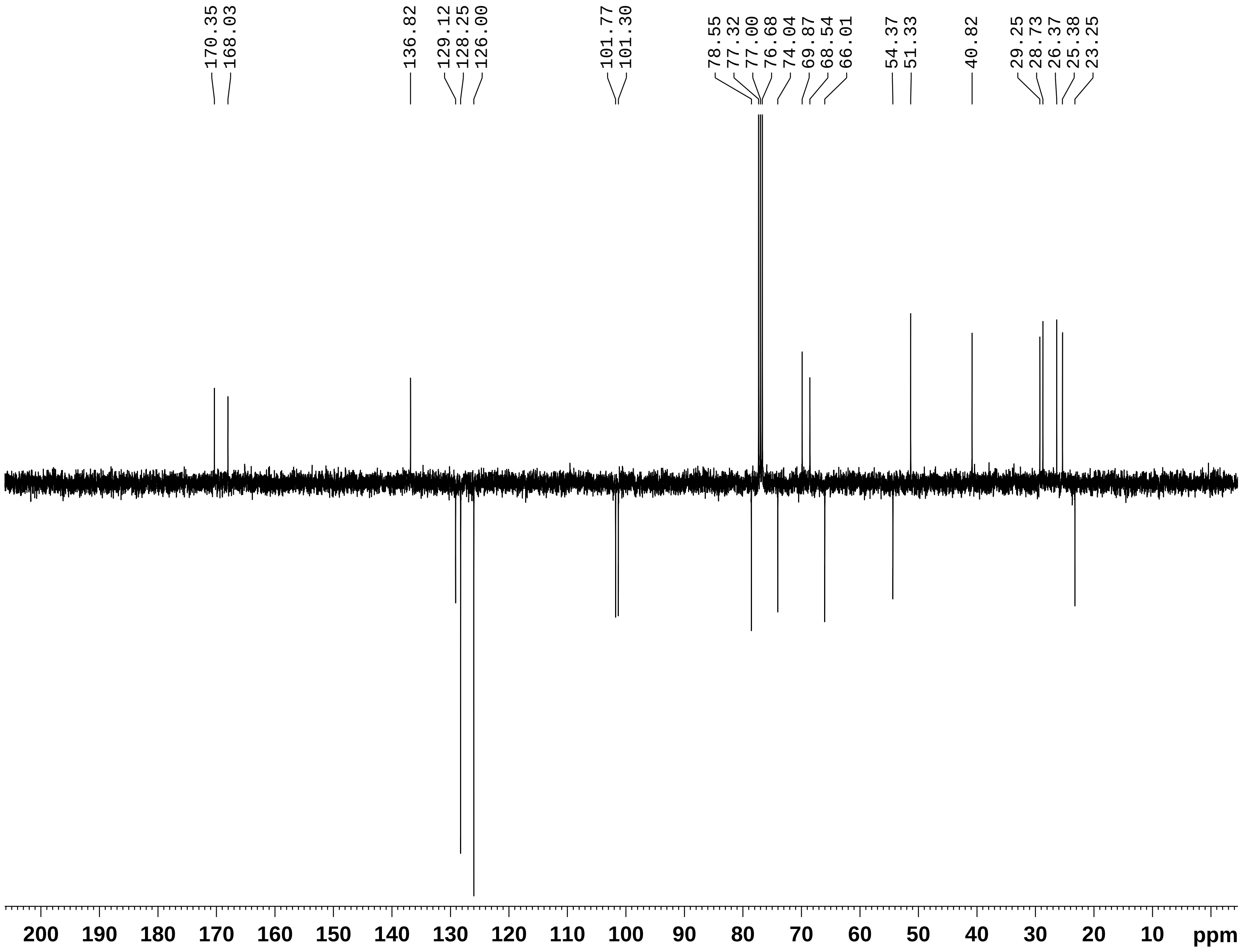

Compound **21**, 400 MHz, $CDCl_3$

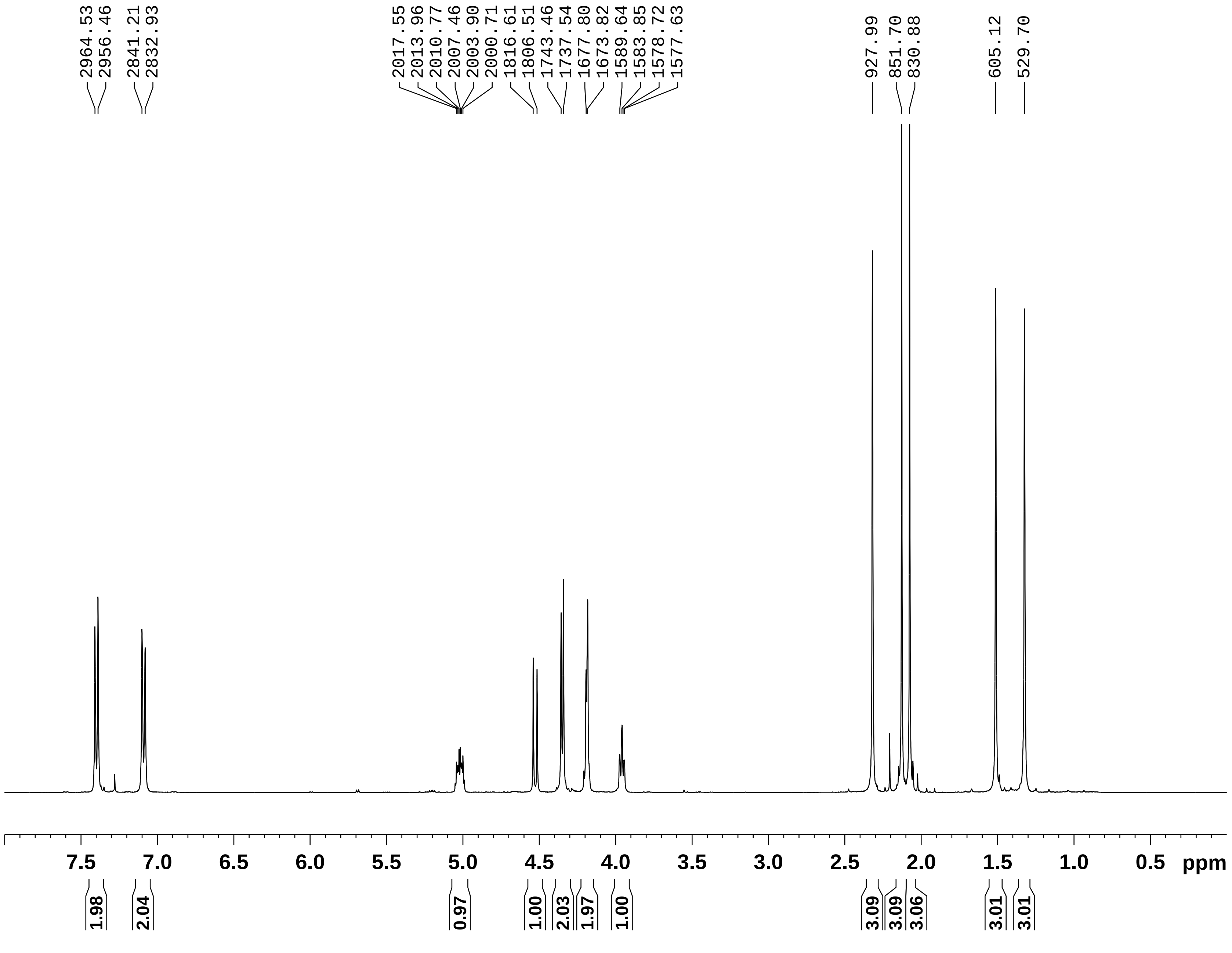

Compound **21**, 100 MHz, $CDCl_3$

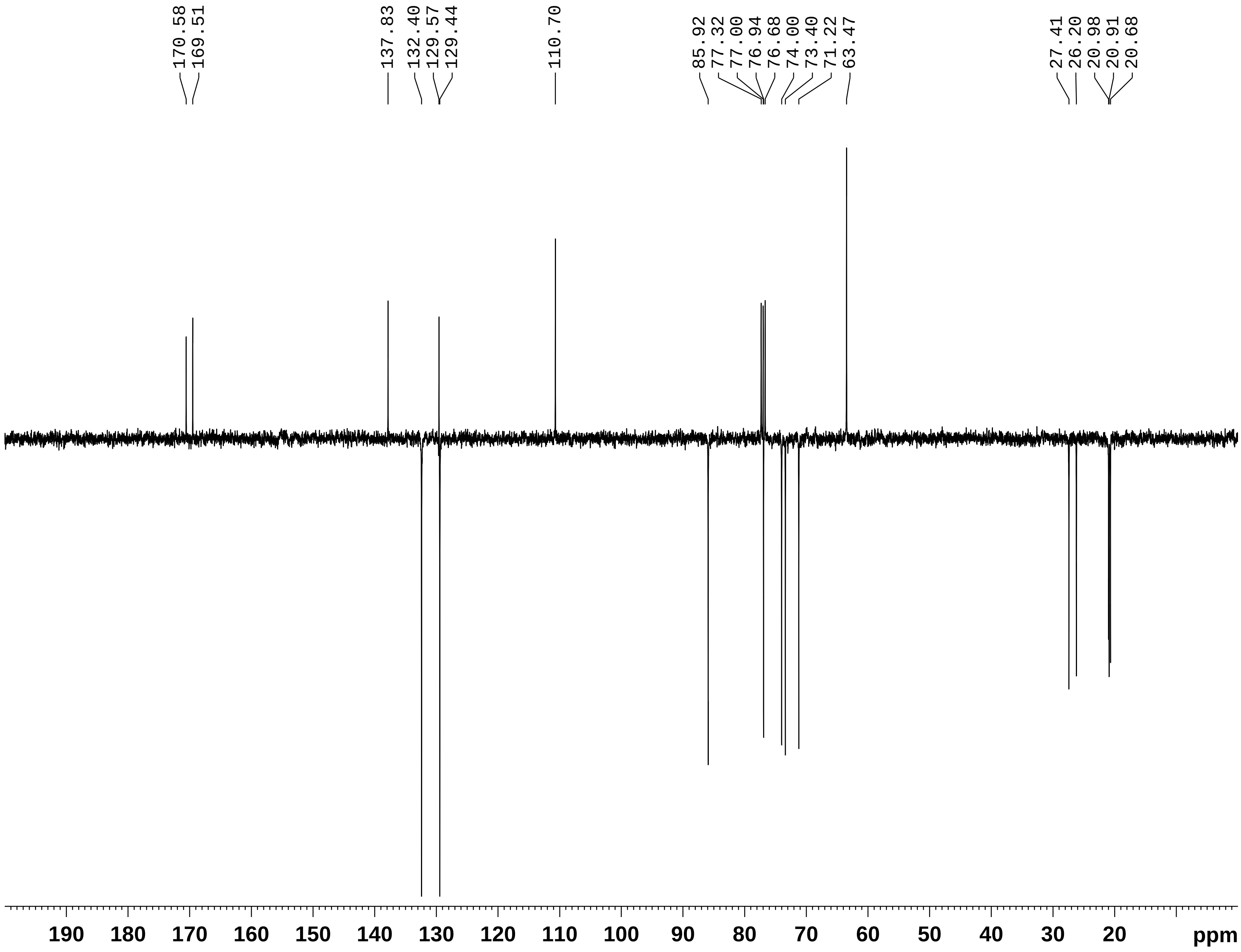

Compound **22**, 400 MHz, $CDCl_3$

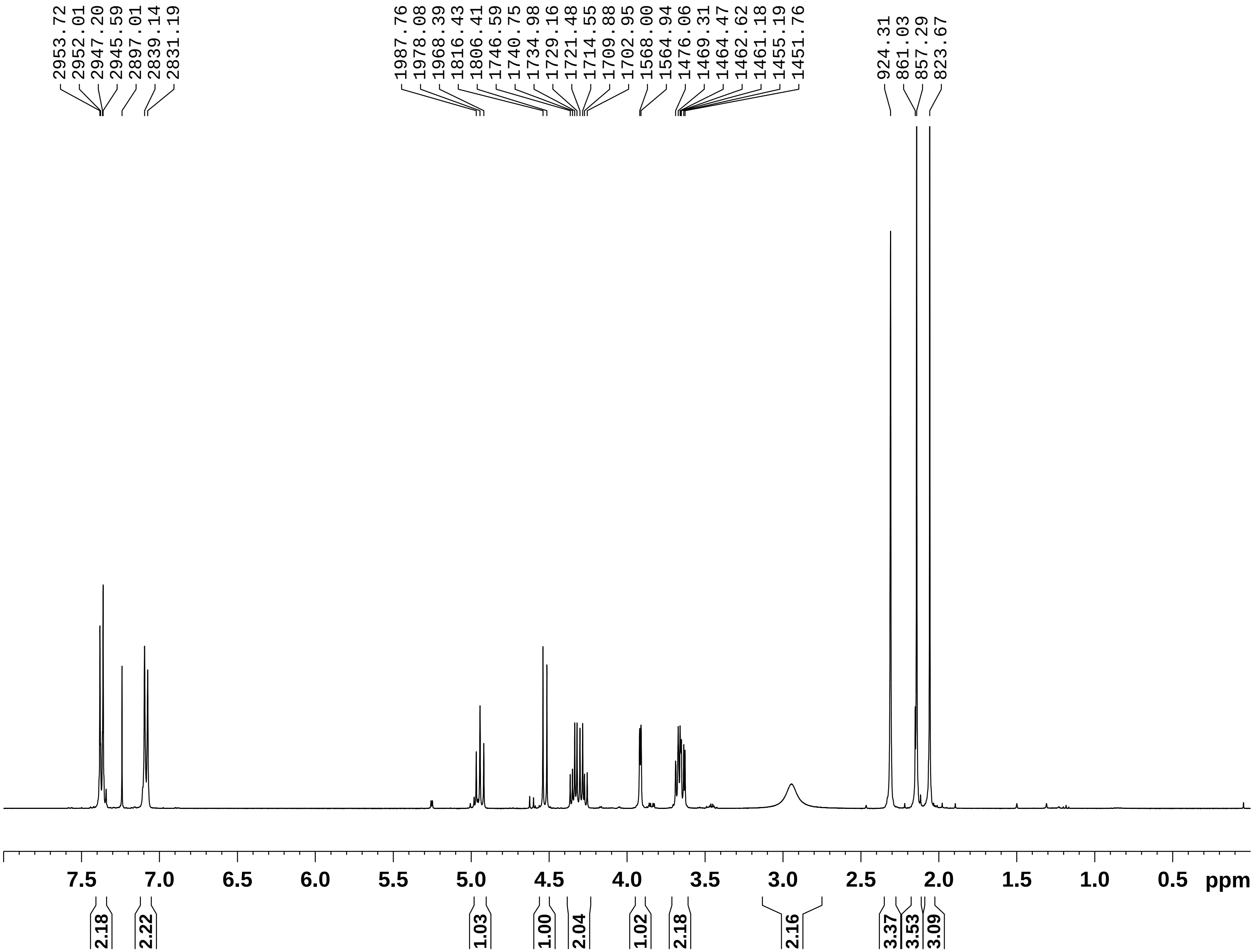

Compound **22**, 100 MHz, $CDCl_3$

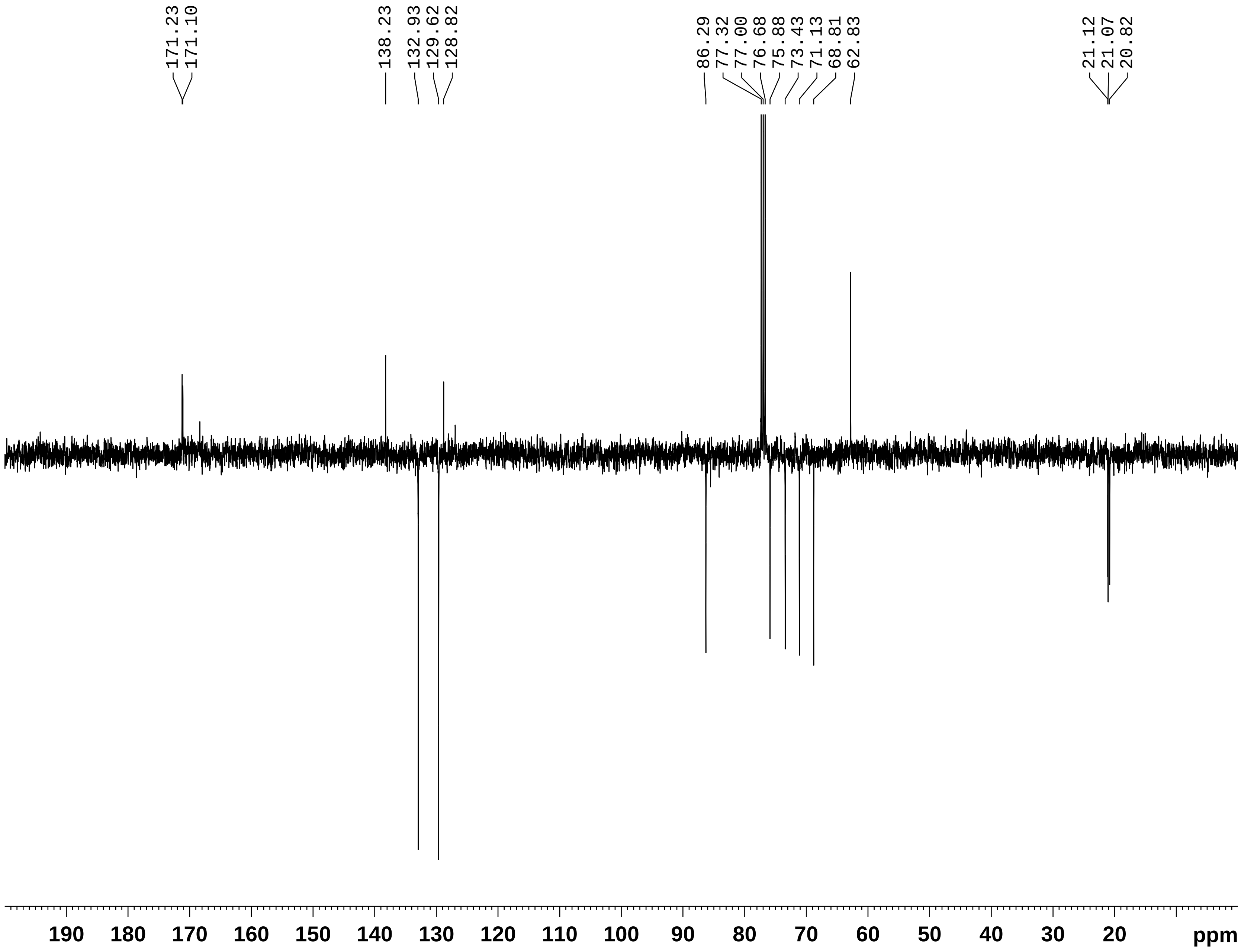

Compound **23**, 400 MHz, $CDCl_3$

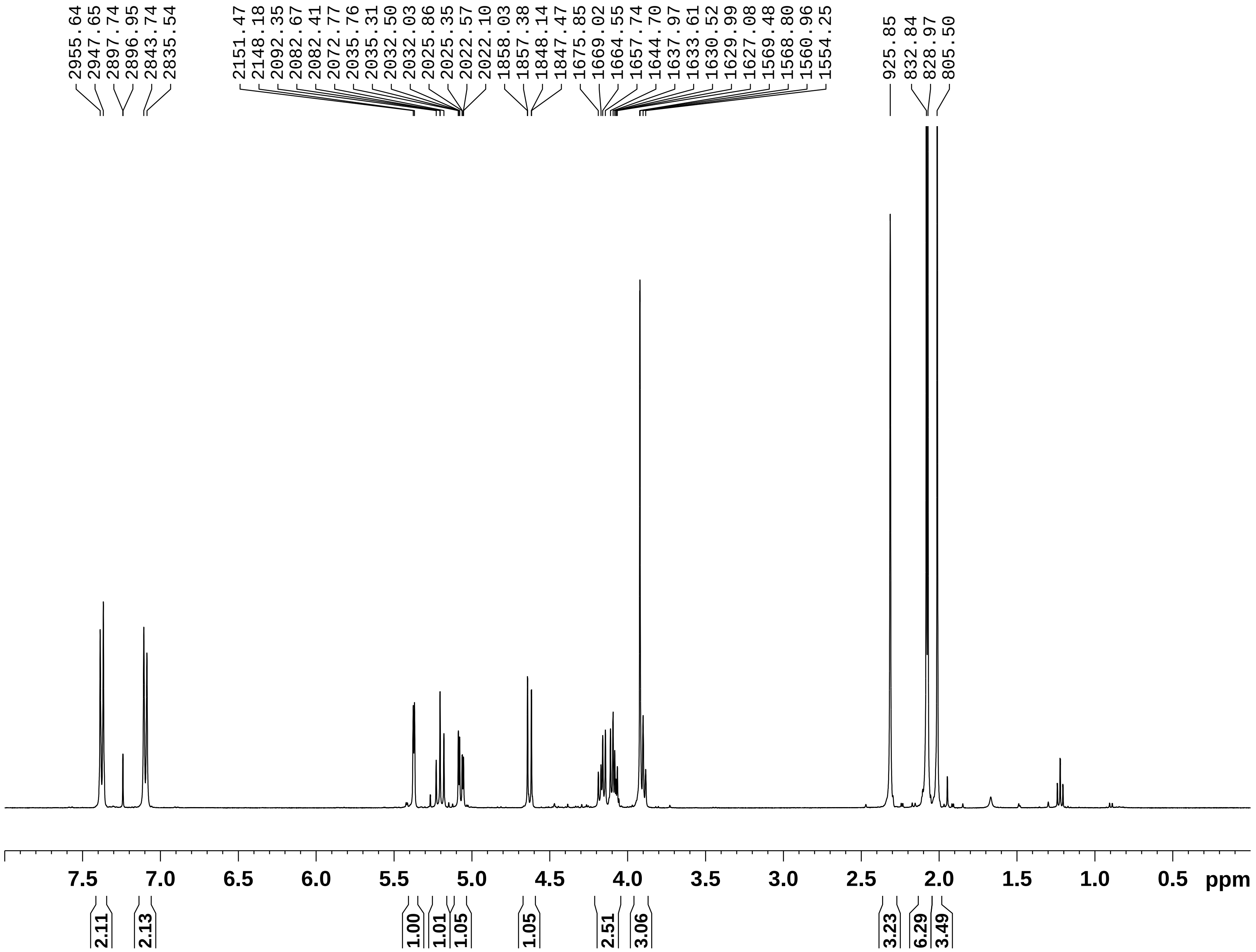

Compound **23**, 100 MHz, $CDCl_3$

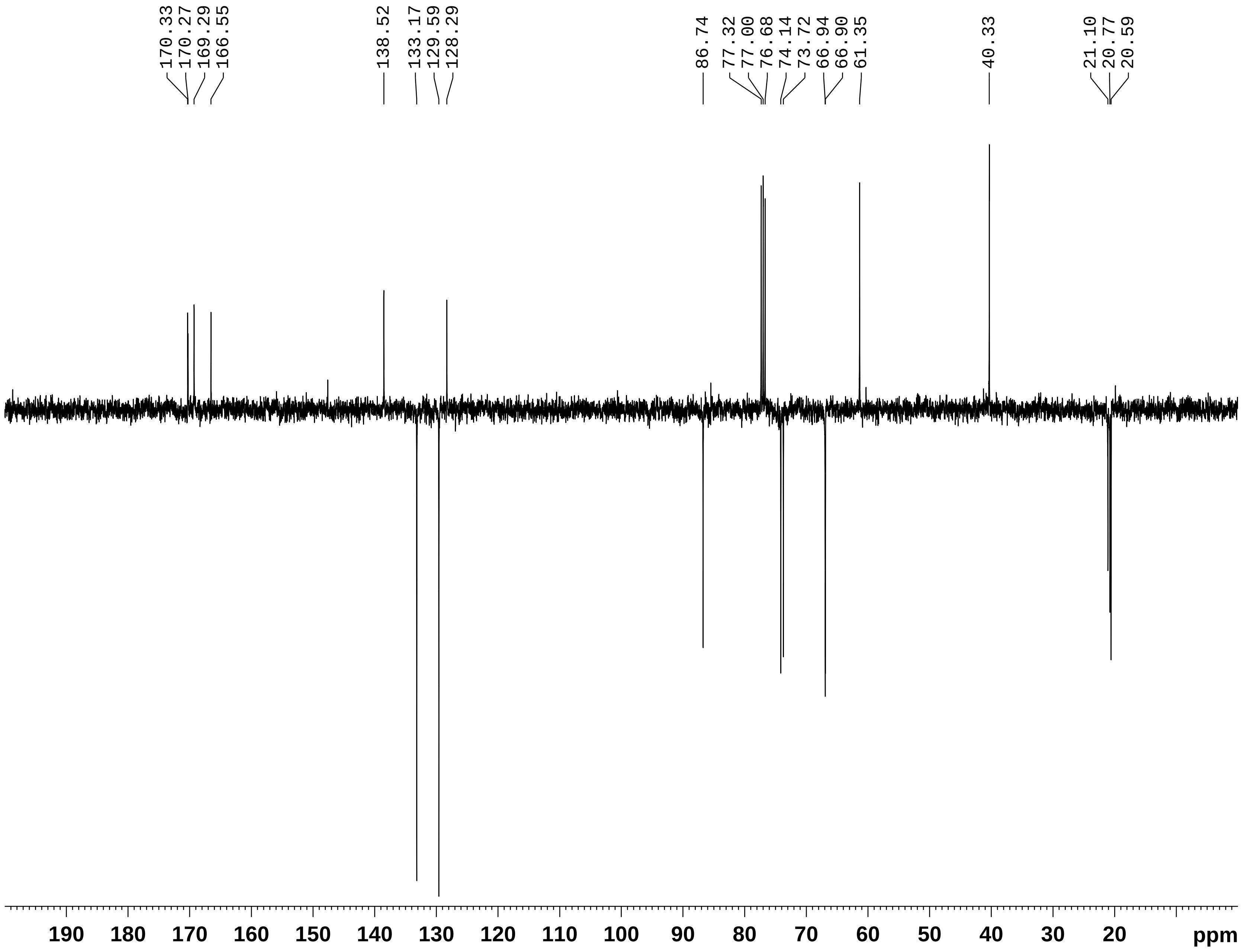

Compound **24**, 400 MHz, $CDCl_3$

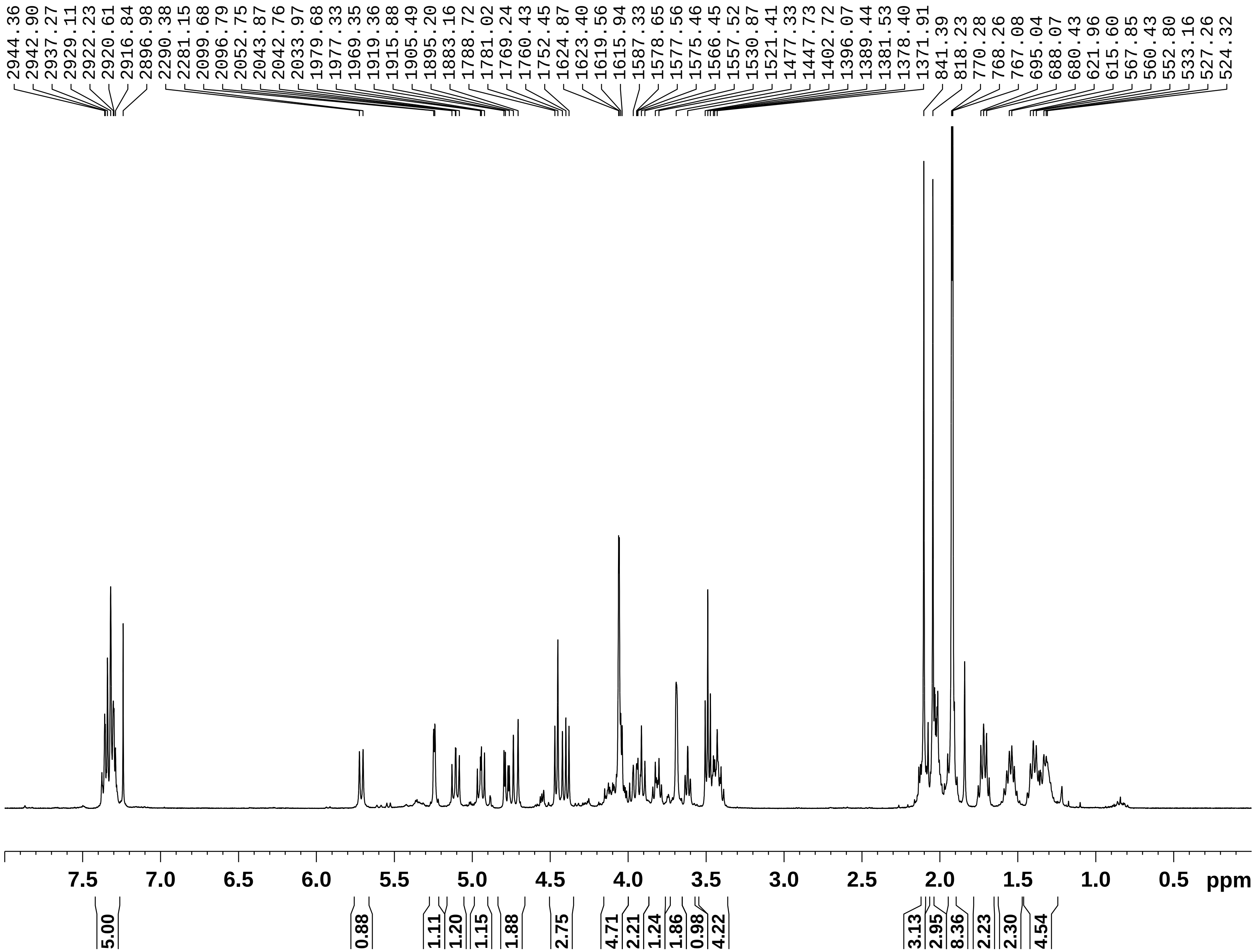

Compound **24**, 100 MHz, $CDCl_3$

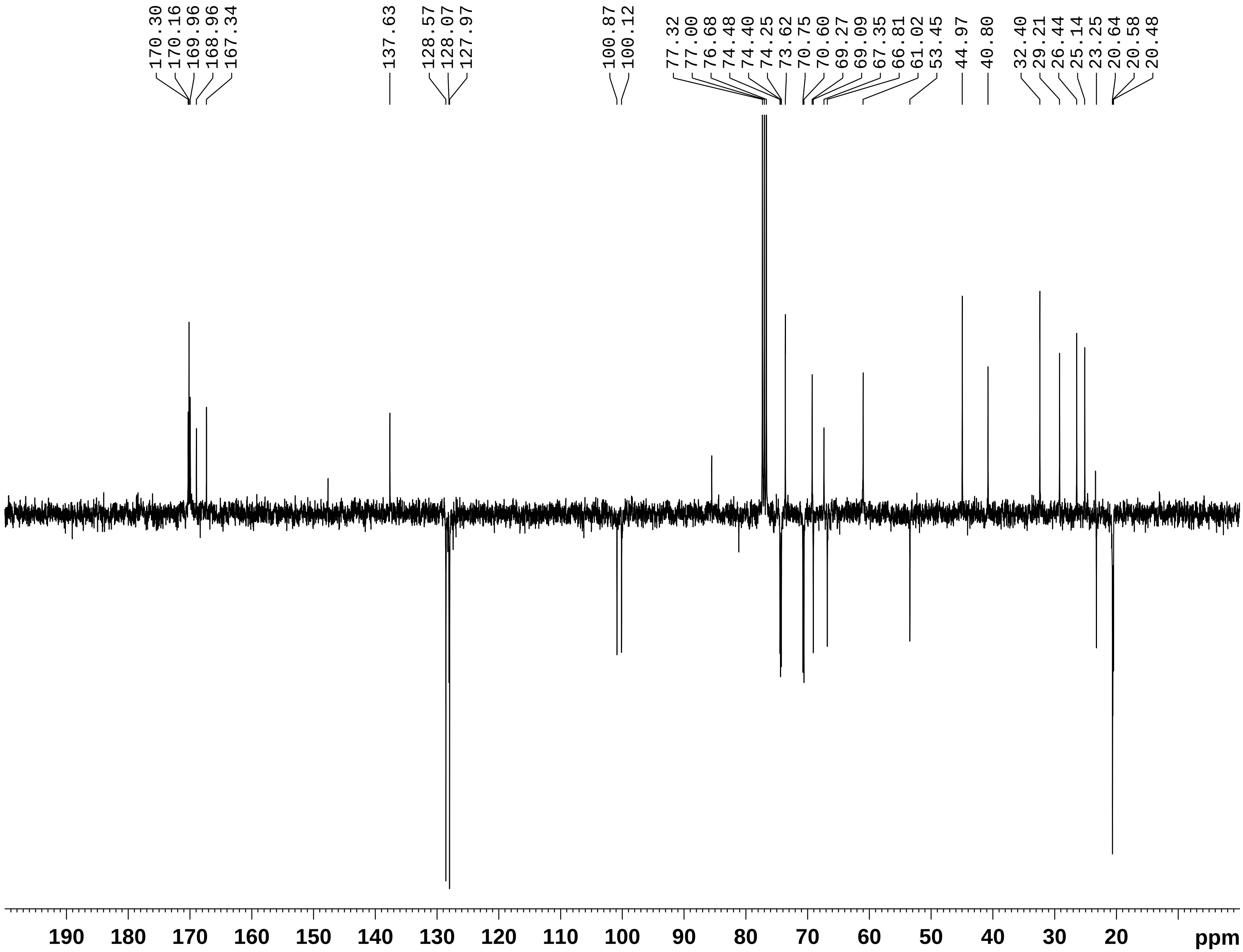

Compound **25**, 400 MHz, $CDCl_3$

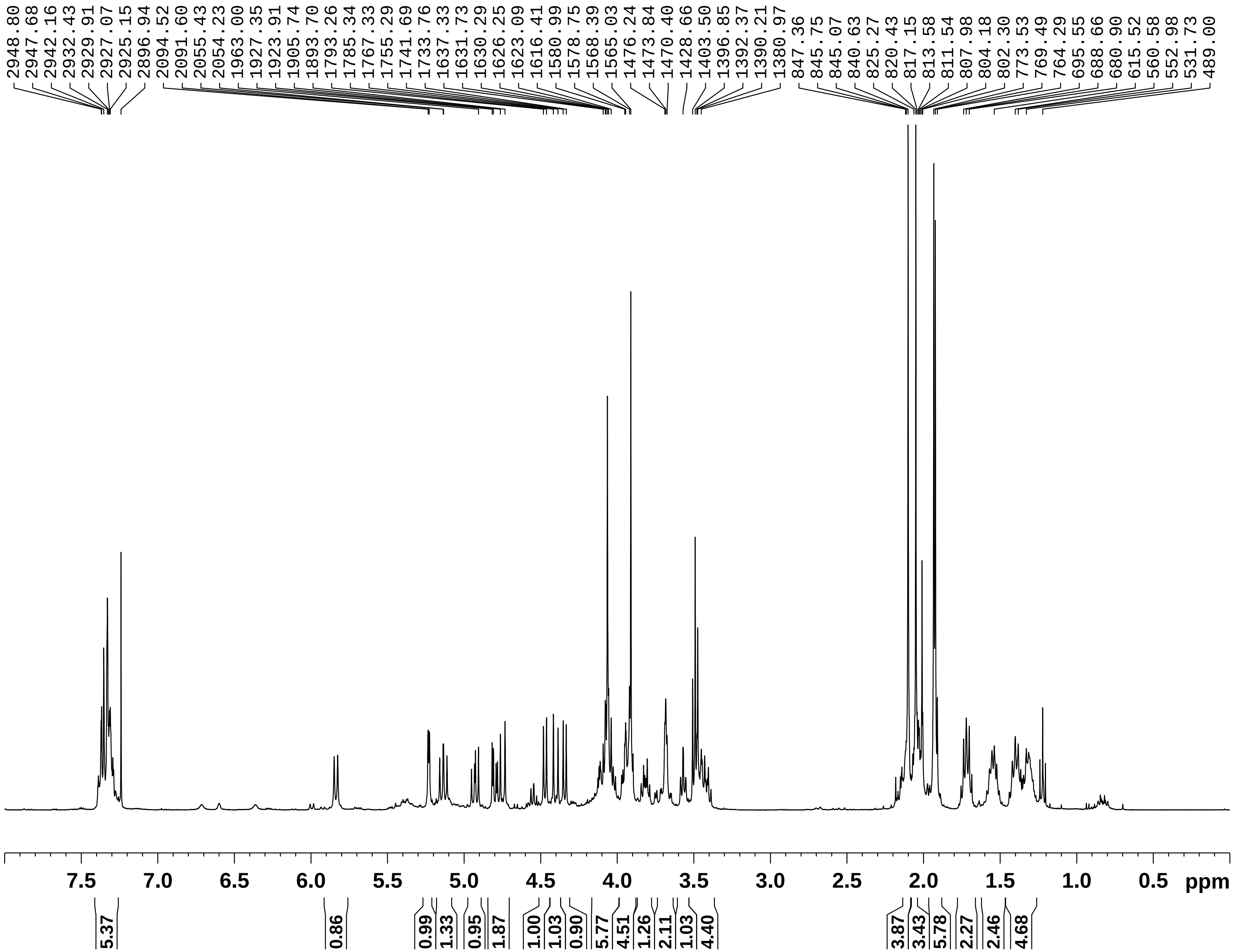

Compound **25**, 100 MHz, $CDCl_3$

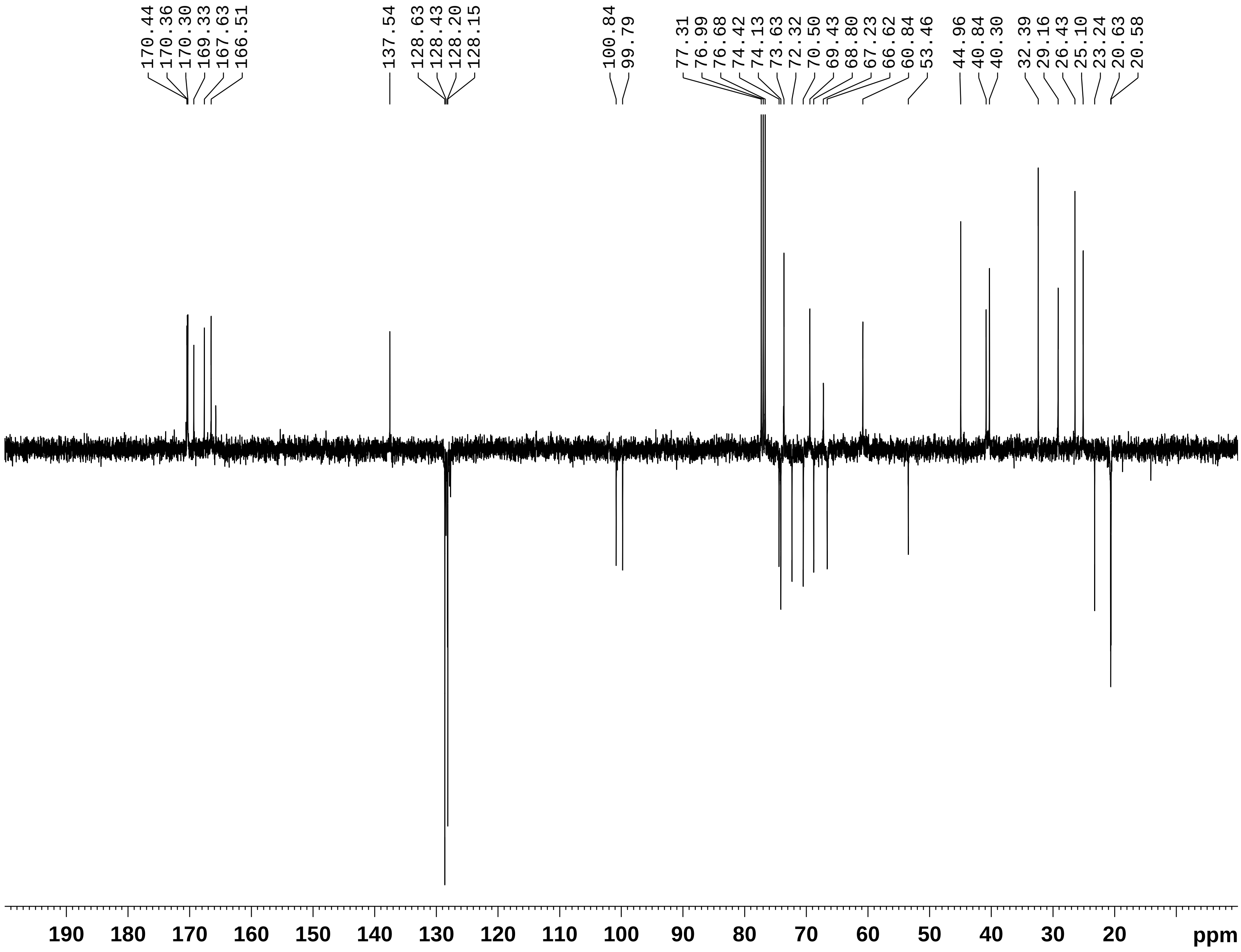

Compound **26**, 400 MHz, $CDCl_3$

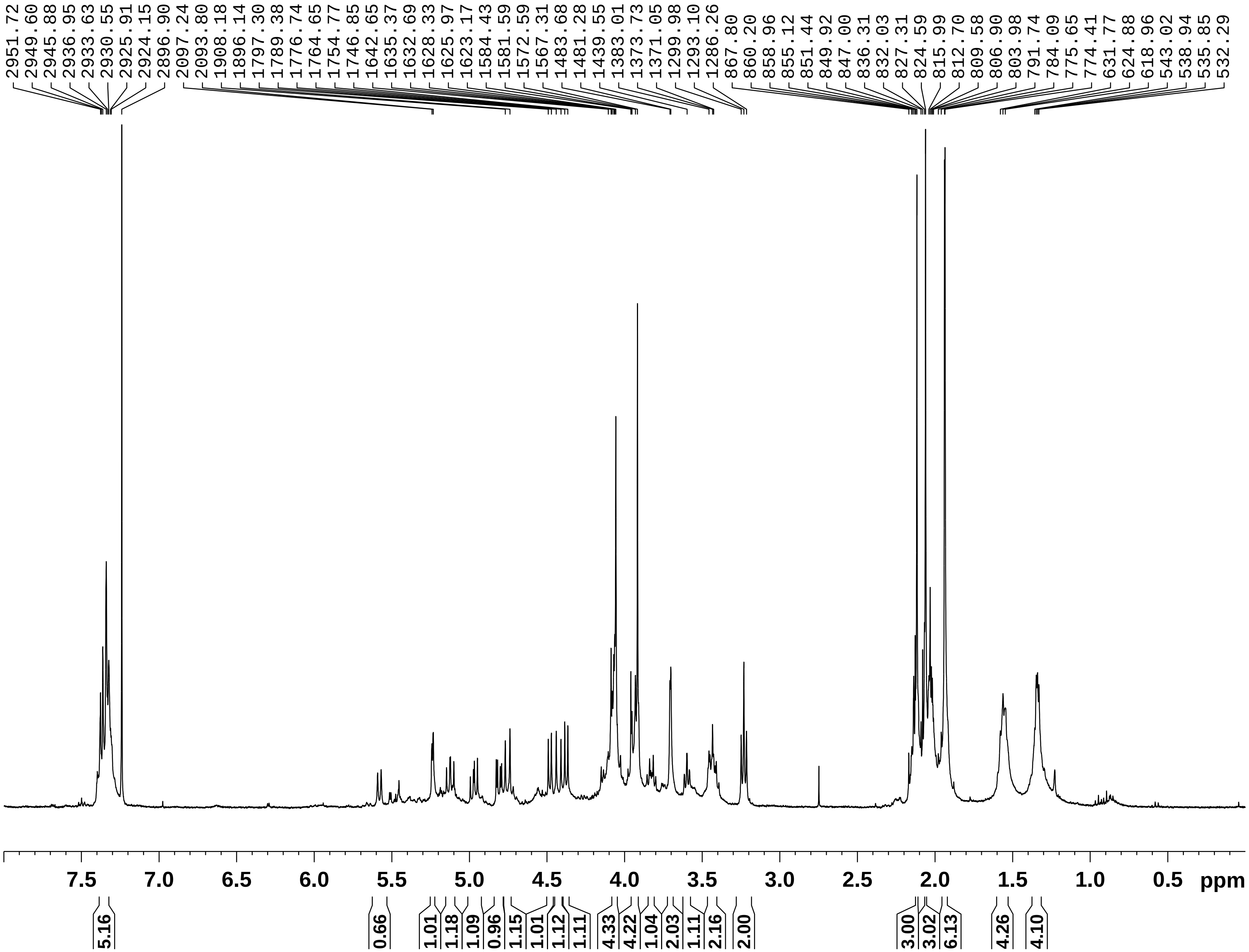

Compound **26**, 100 MHz, $CDCl_3$

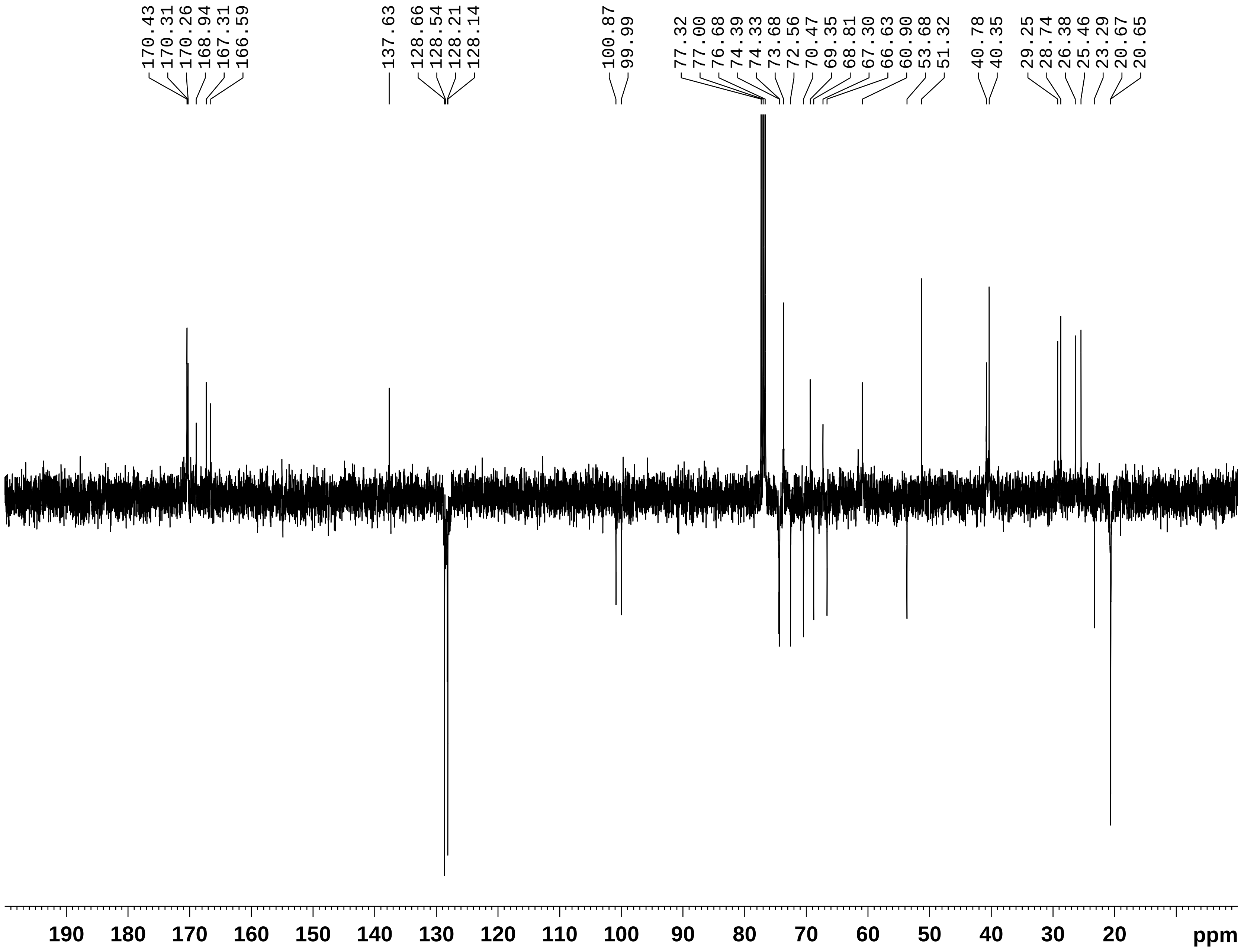

Compound **27**, 400 MHz, $CDCl_3$

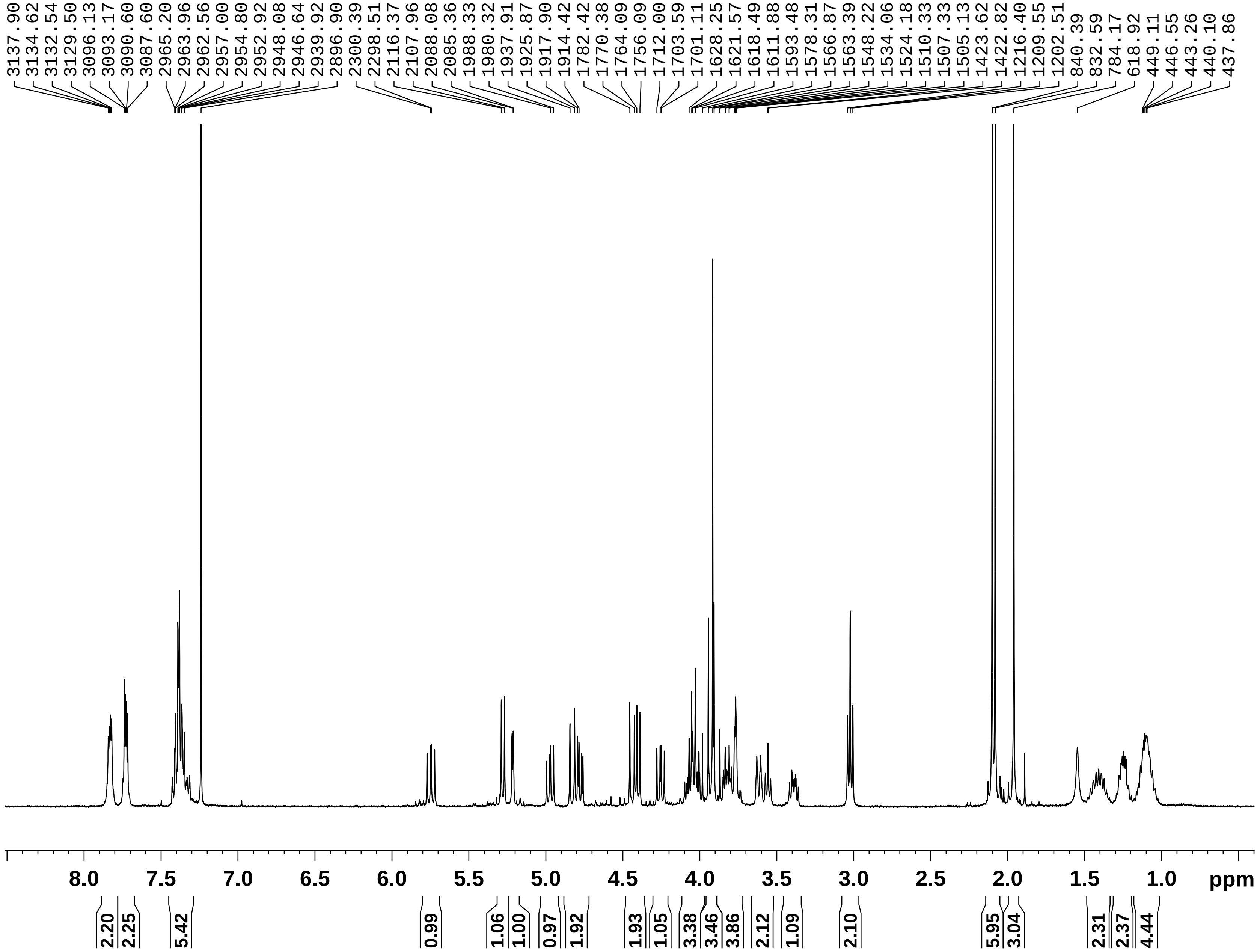

Compound **27**, 100 MHz, $CDCl_3$

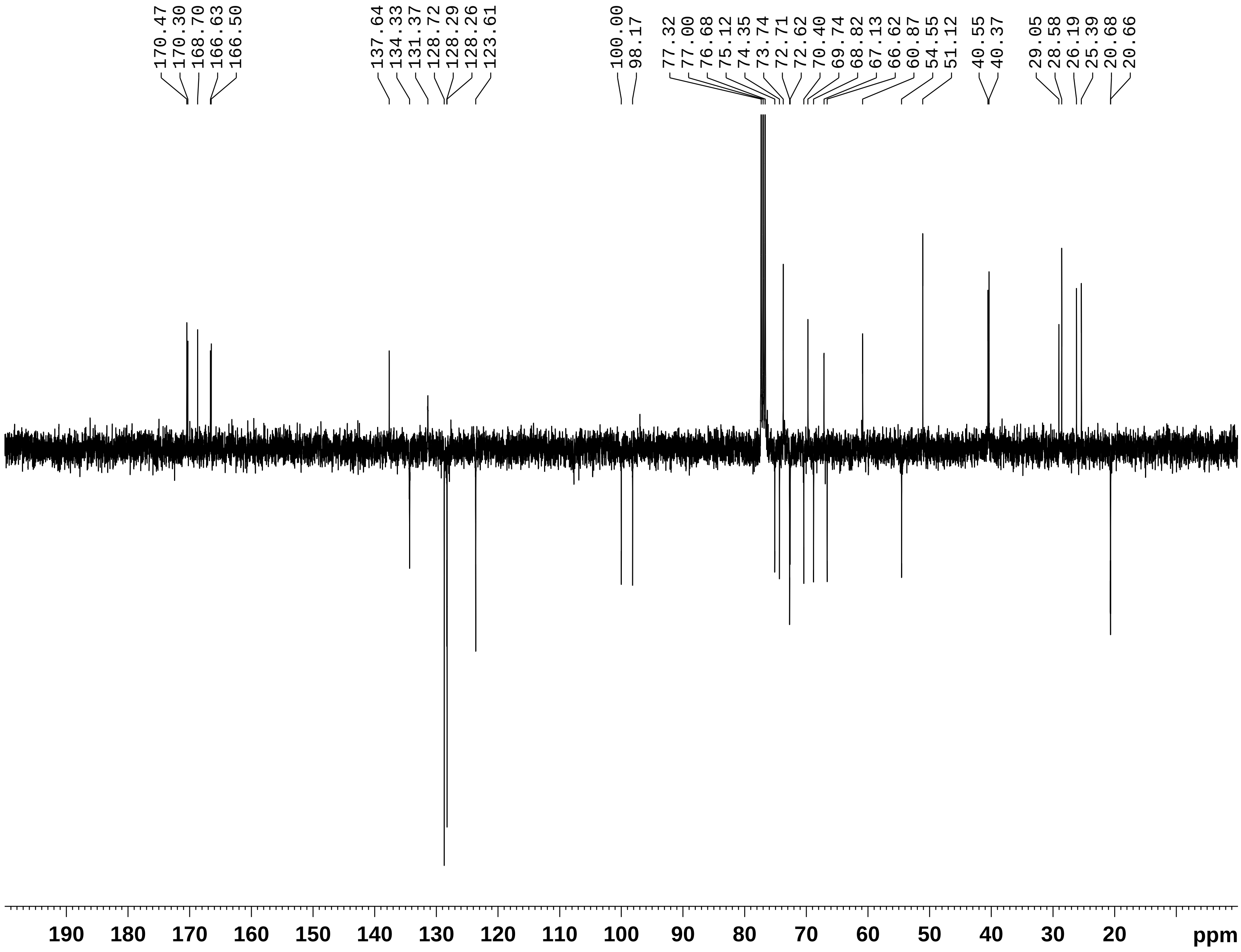

Compound **28**, 400 MHz, $CDCl_3$

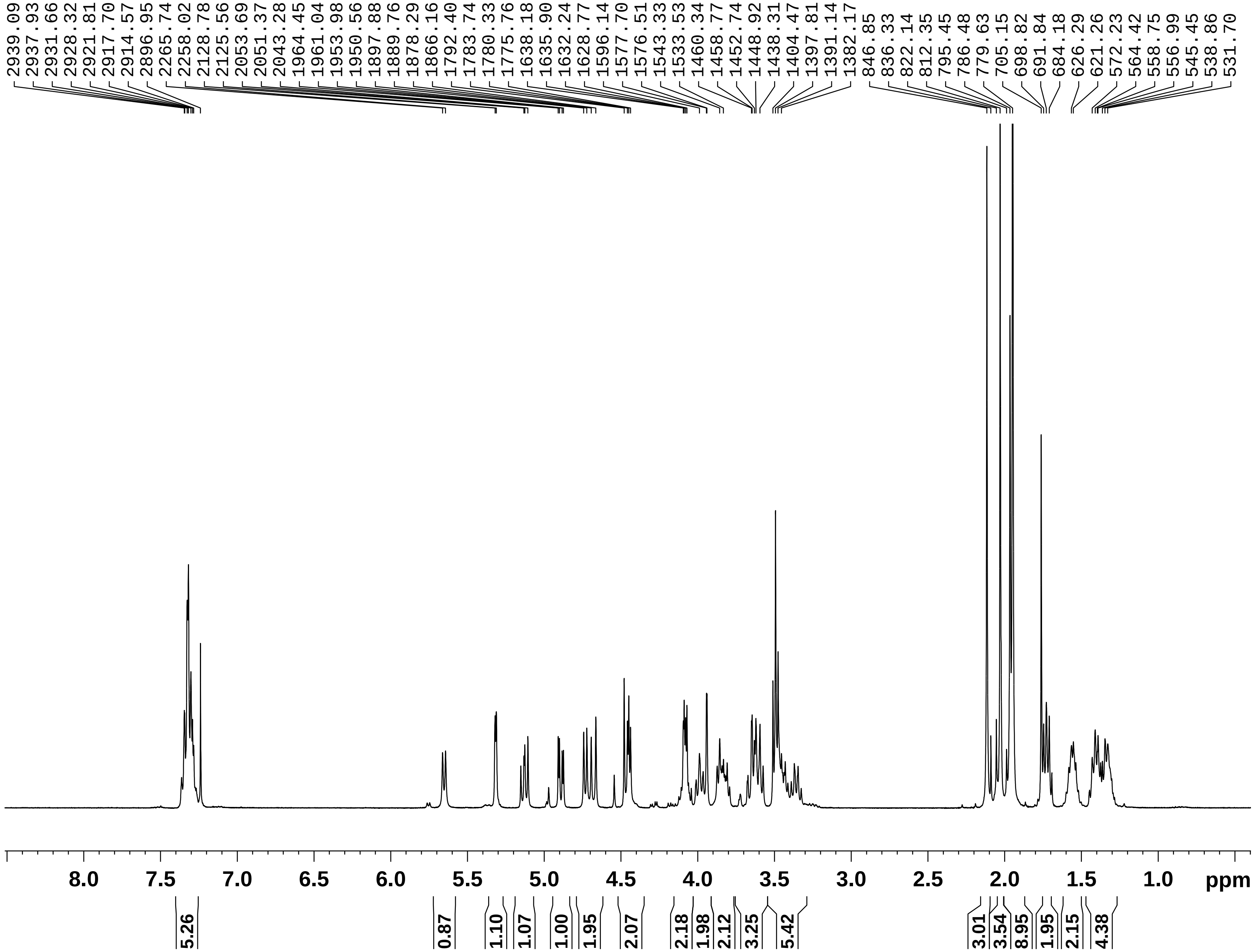

Compound **28**, 100 MHz, $CDCl_3$

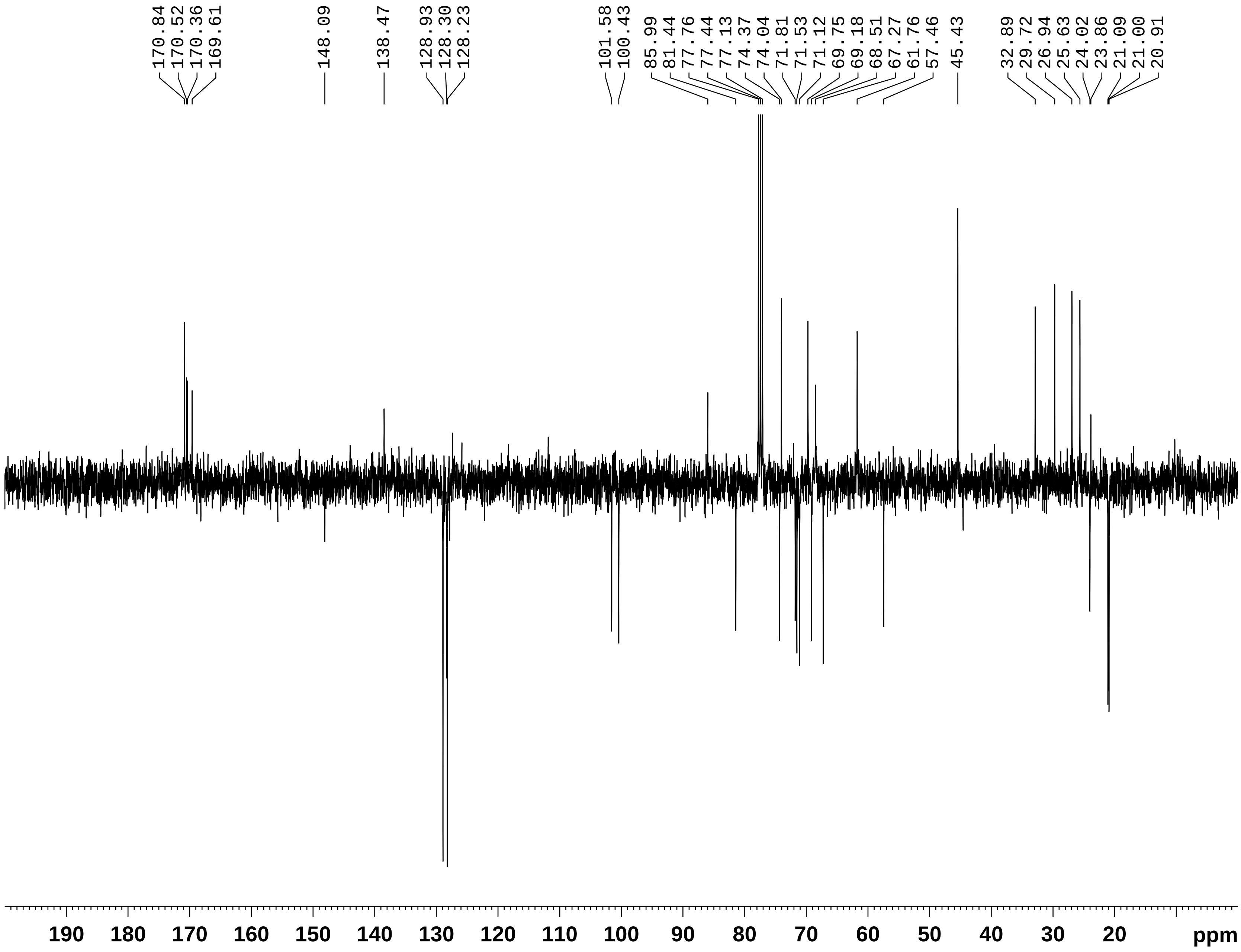

Compound **29**, 600 MHz, $CDCl_3$

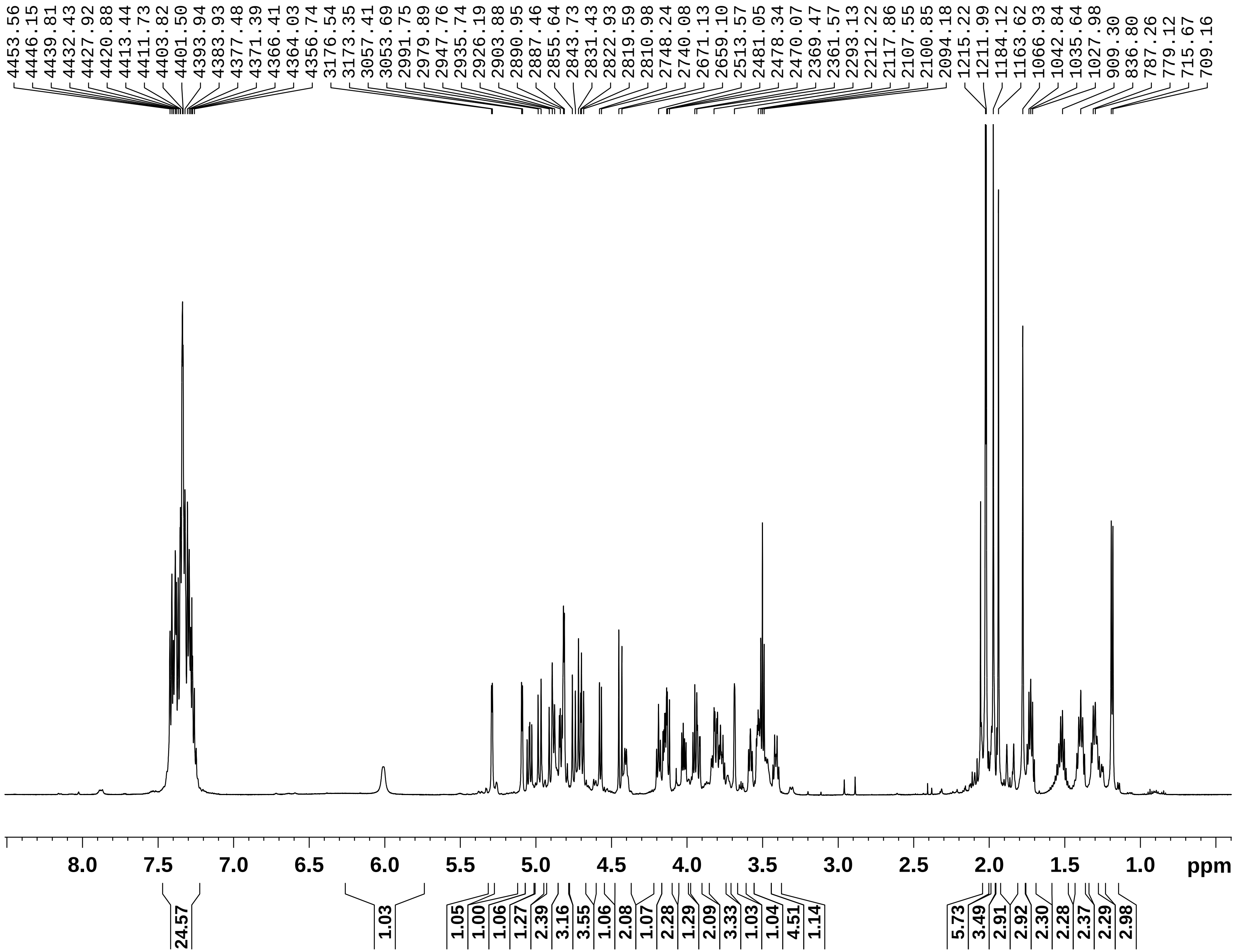

Compound **29**, 150 MHz, $CDCl_3$

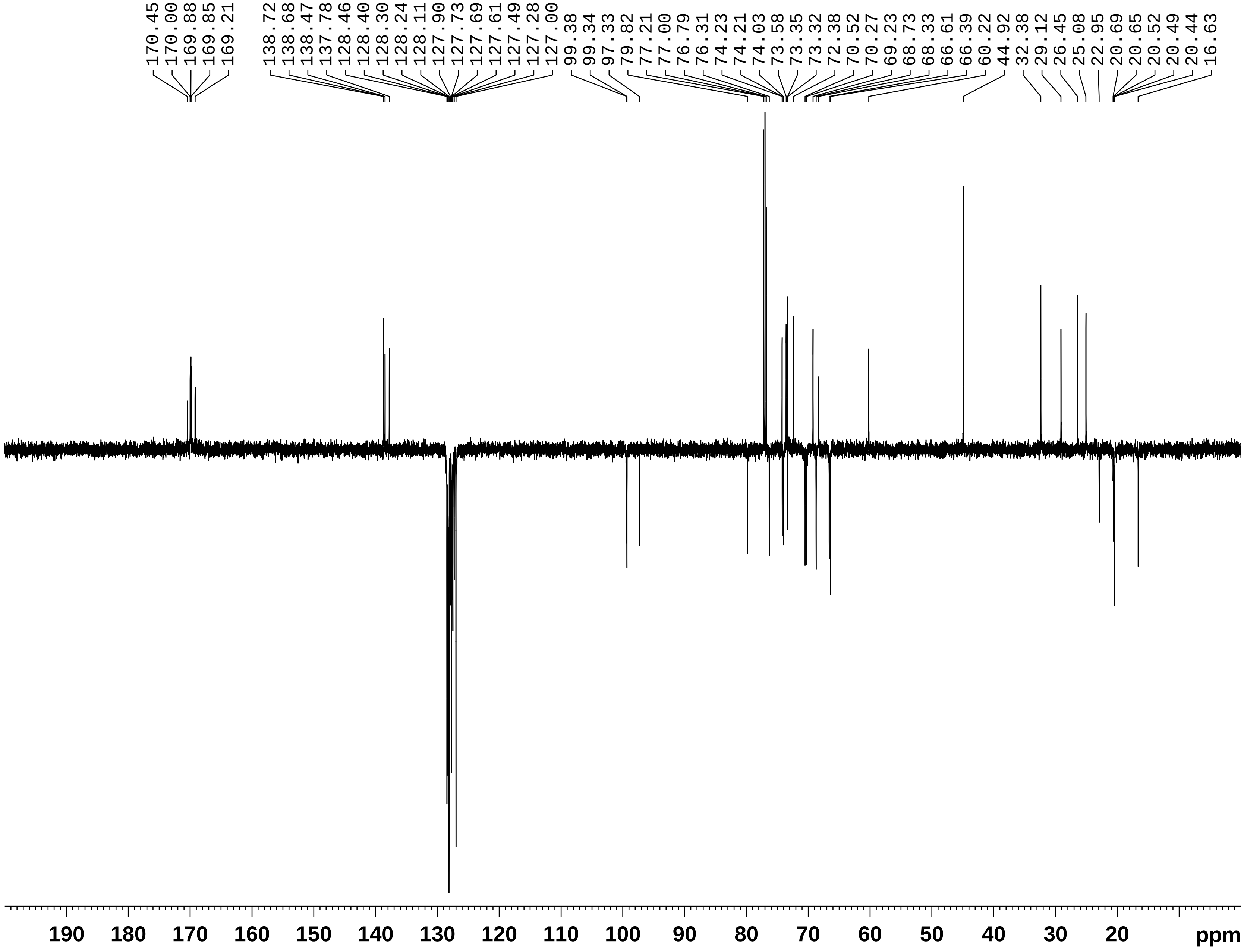

Compound **30**, 400 MHz, $CDCl_3$

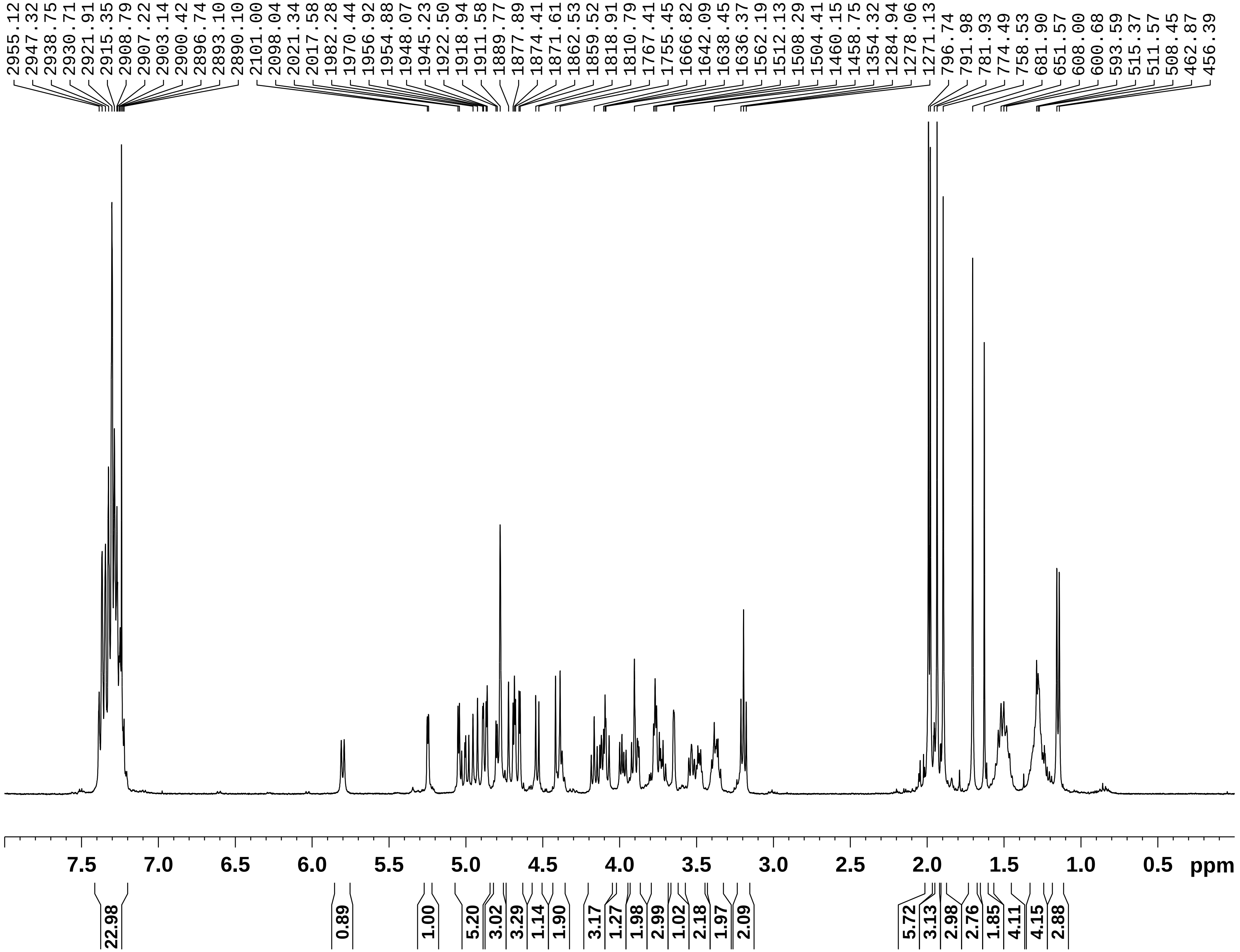

Compound **30**, 100 MHz, $CDCl_3$

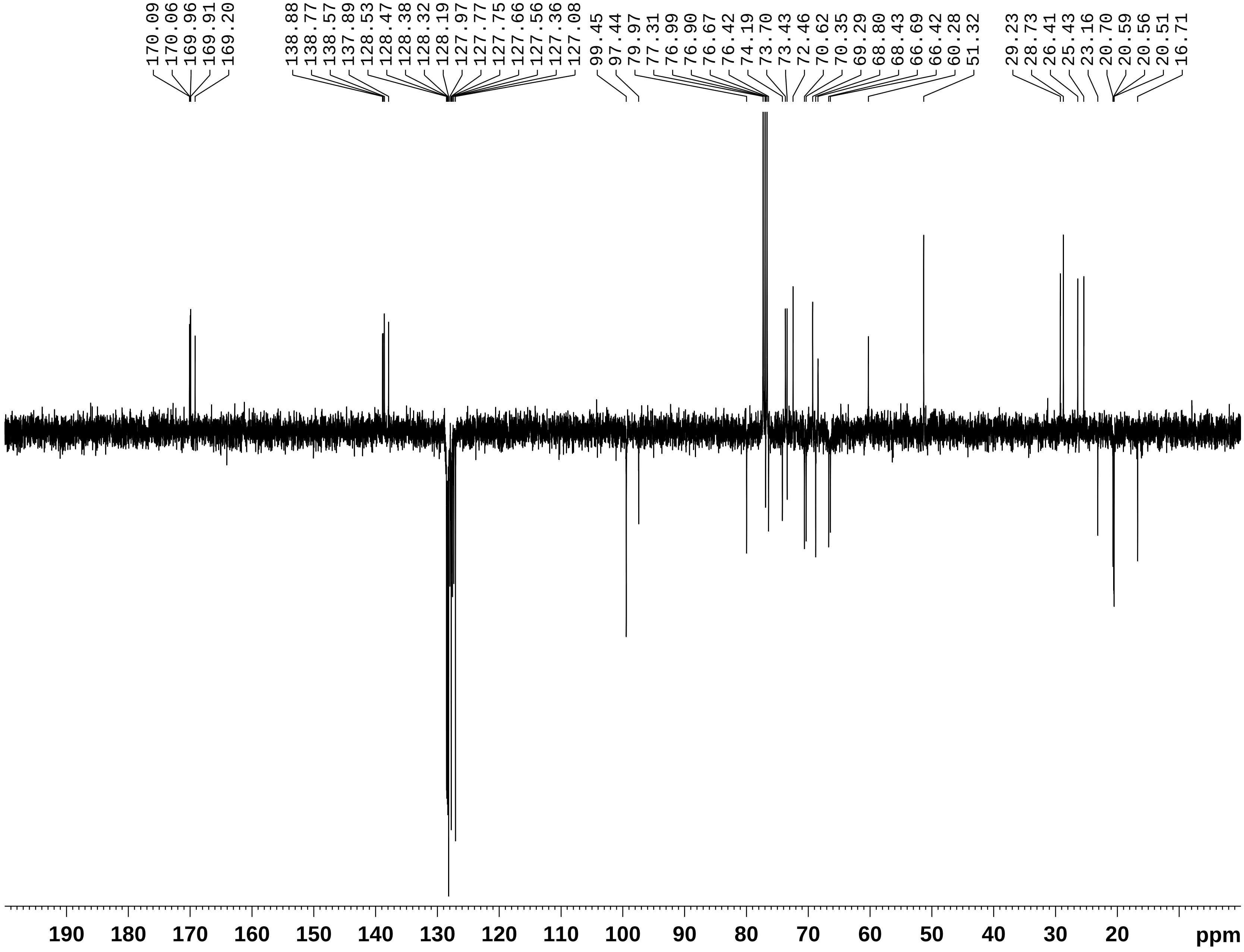

Compound **31**, 400 MHz, $CDCl_3$

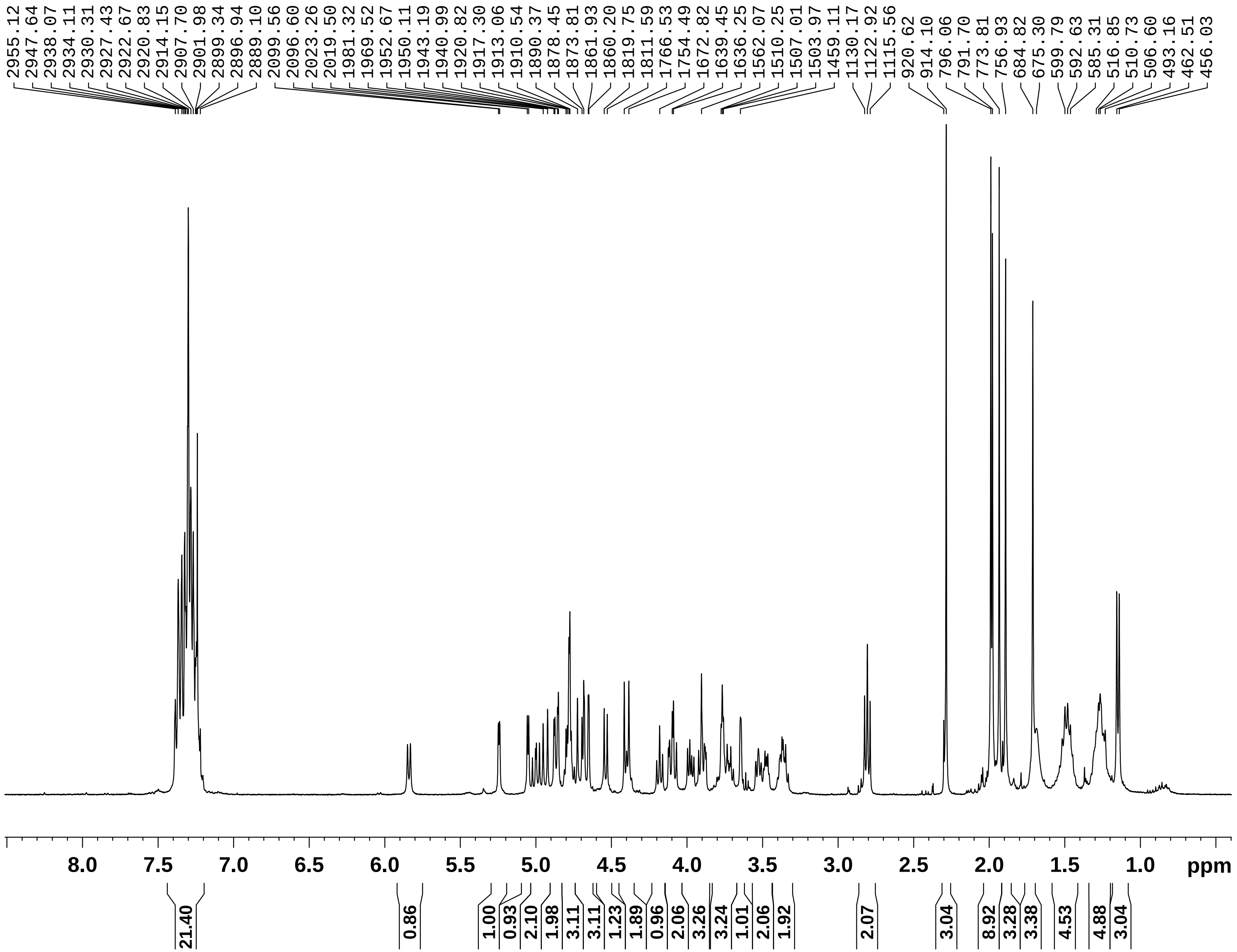

Compound **31**, 100 MHz, $CDCl_3$

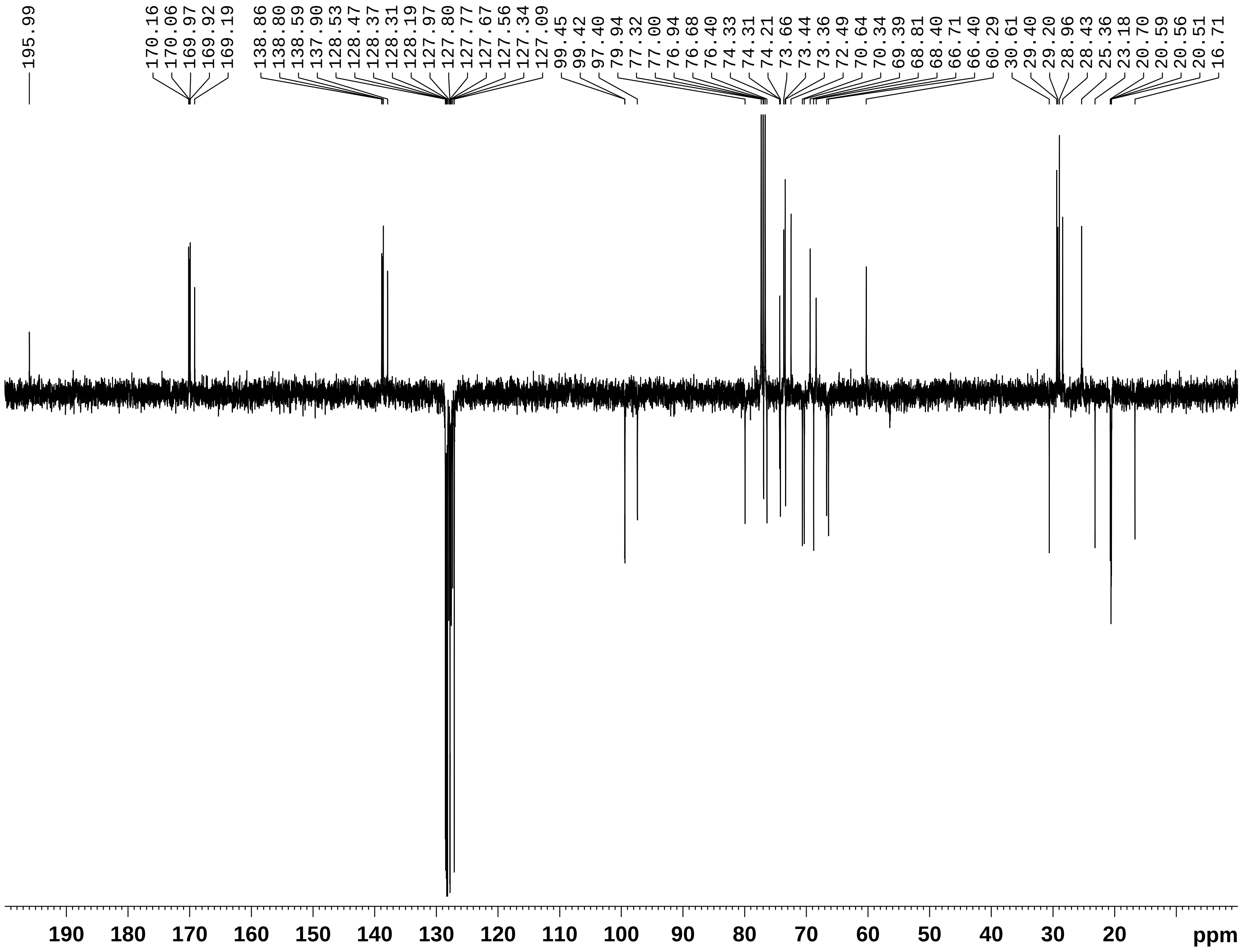

Compound **32**, 400 MHz, $CDCl_3$

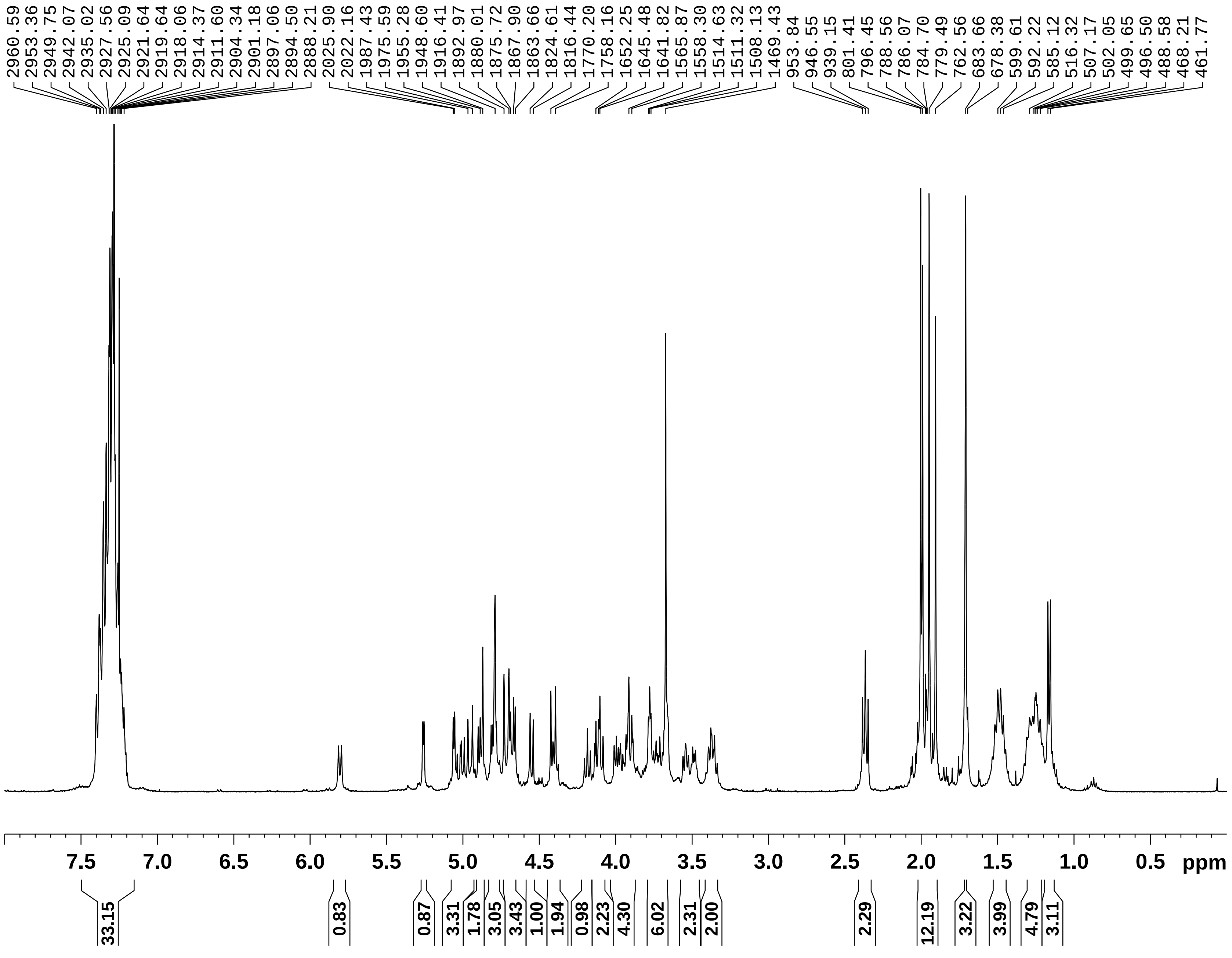

Compound **32**, 150 MHz, $CDCl_3$

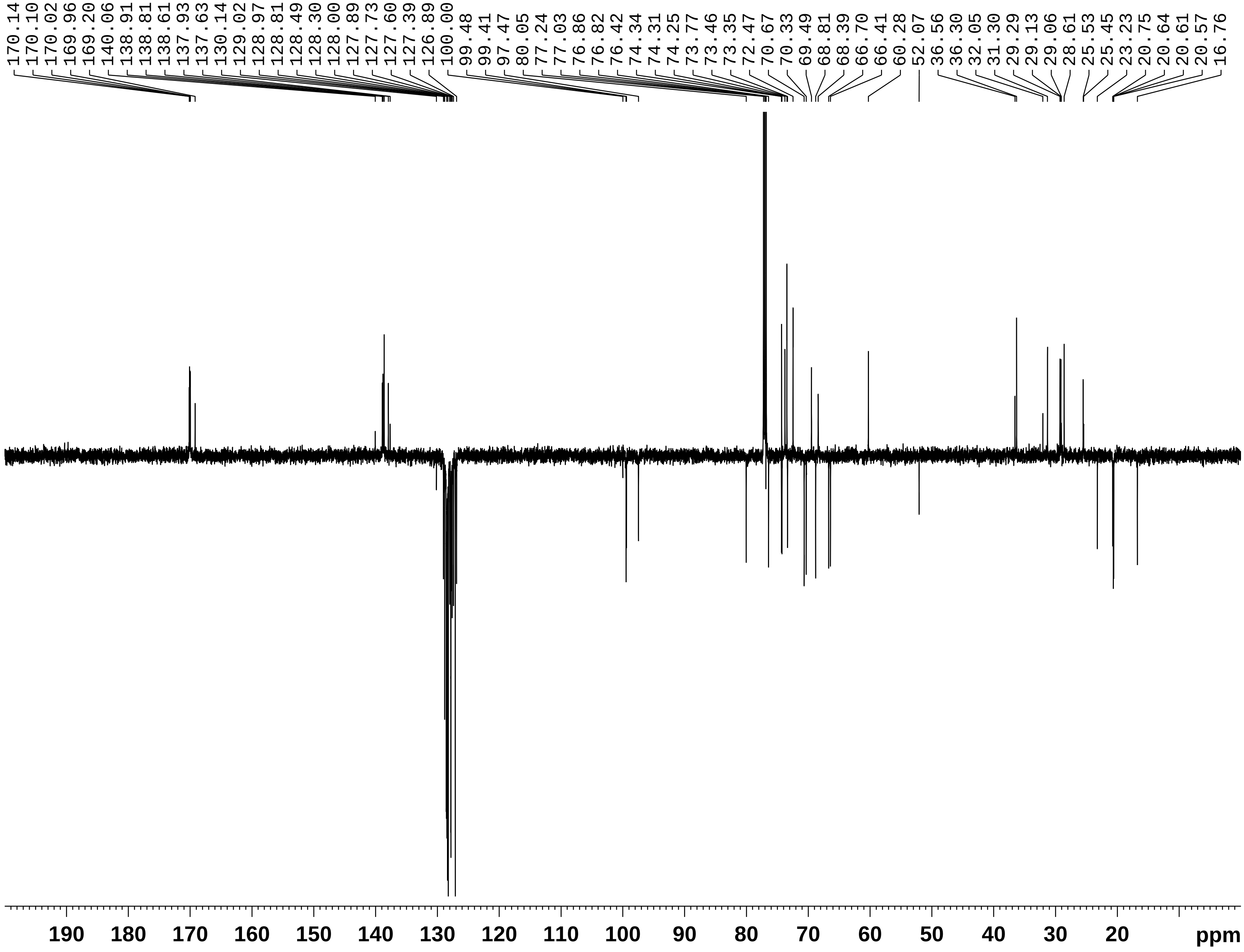

Supplement: File 2 — 1H and 13C NMR spectra for compounds 1–6, 8, 11, 12, 16–19, 21–32, 1H NMR data for known compounds 14, 15. [file Beilstein_J_Org_Chem-06-17-s002.pdf]
